# Correction: A dynamic genetic-hormonal regulatory network model explains multiple cellular behaviors of the root apical meristem of *Arabidopsis thaliana*

**DOI:** 10.1371/journal.pcbi.1007140

**Published:** 2019-06-12

**Authors:** The PLOS Computational Biology Staff

## Notice of republication

This article was republished on October 18, 2017, to correct errors that were introduced during the typesetting process. The publisher apologizes for the errors. Please download this article again to view the correct version.

## Supporting information

S1 File Republished, corrected article. (PDF)
