# Supplementary figures and images for: A dynamic genetic-hormonal regulatory network model explains multiple cellular behaviors of the root apical meristem of Arabidopsis thaliana
Source: PLoS Comput Biol. 2017 Apr 20;13(4):e1005488. doi: 10.1371/journal.pcbi.1005488 (PMC5417714; doi:10.1371/journal.pcbi.1005488)

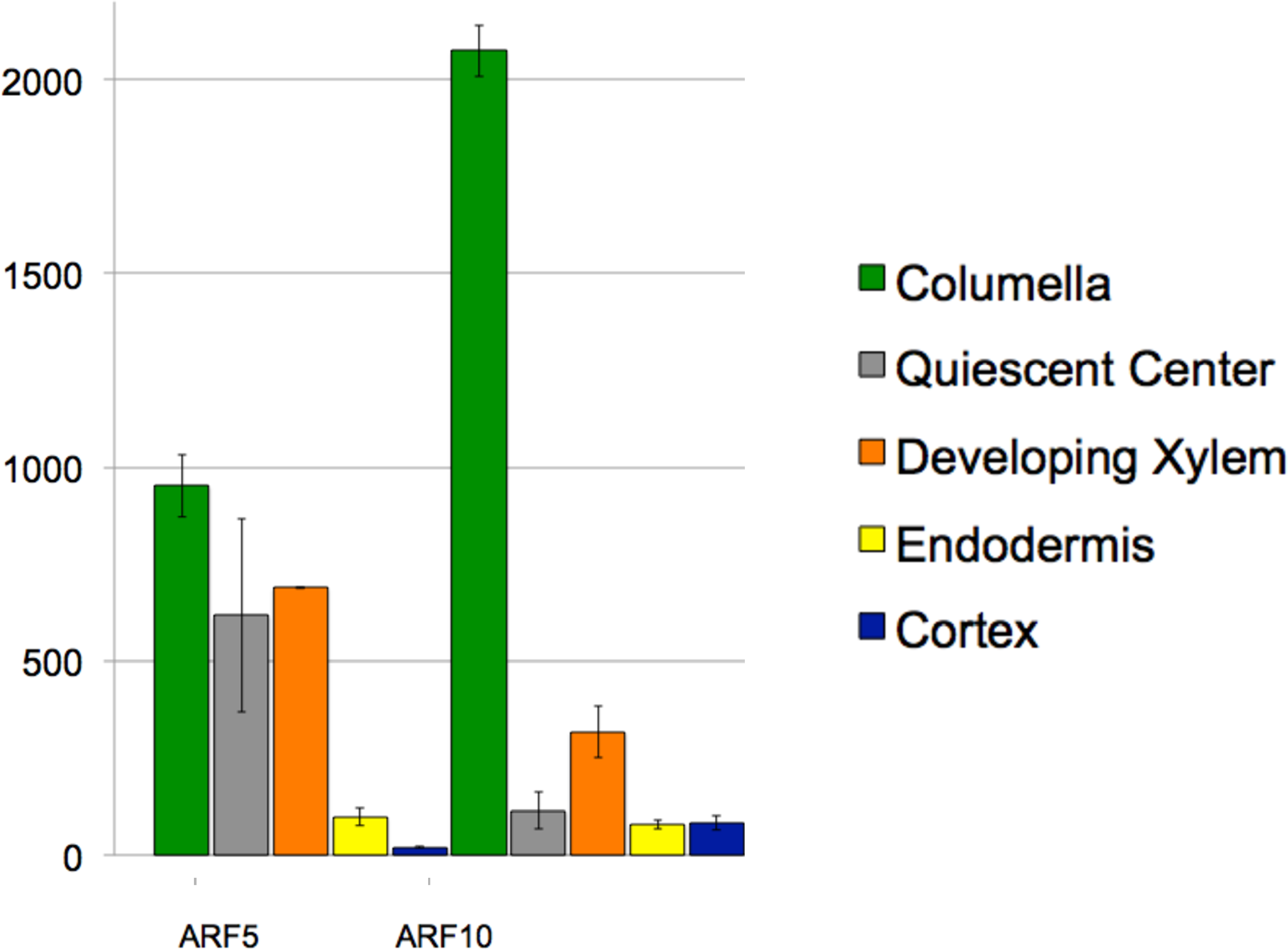

Supplement: S1 Fig — Expression of ARF5 and ARF10 in the columella, QC, developing xylem, endodermis and cortex cells (data taken from [89]). Bars represent the standard error. (TIF) [file pcbi.1005488.s001.tif]

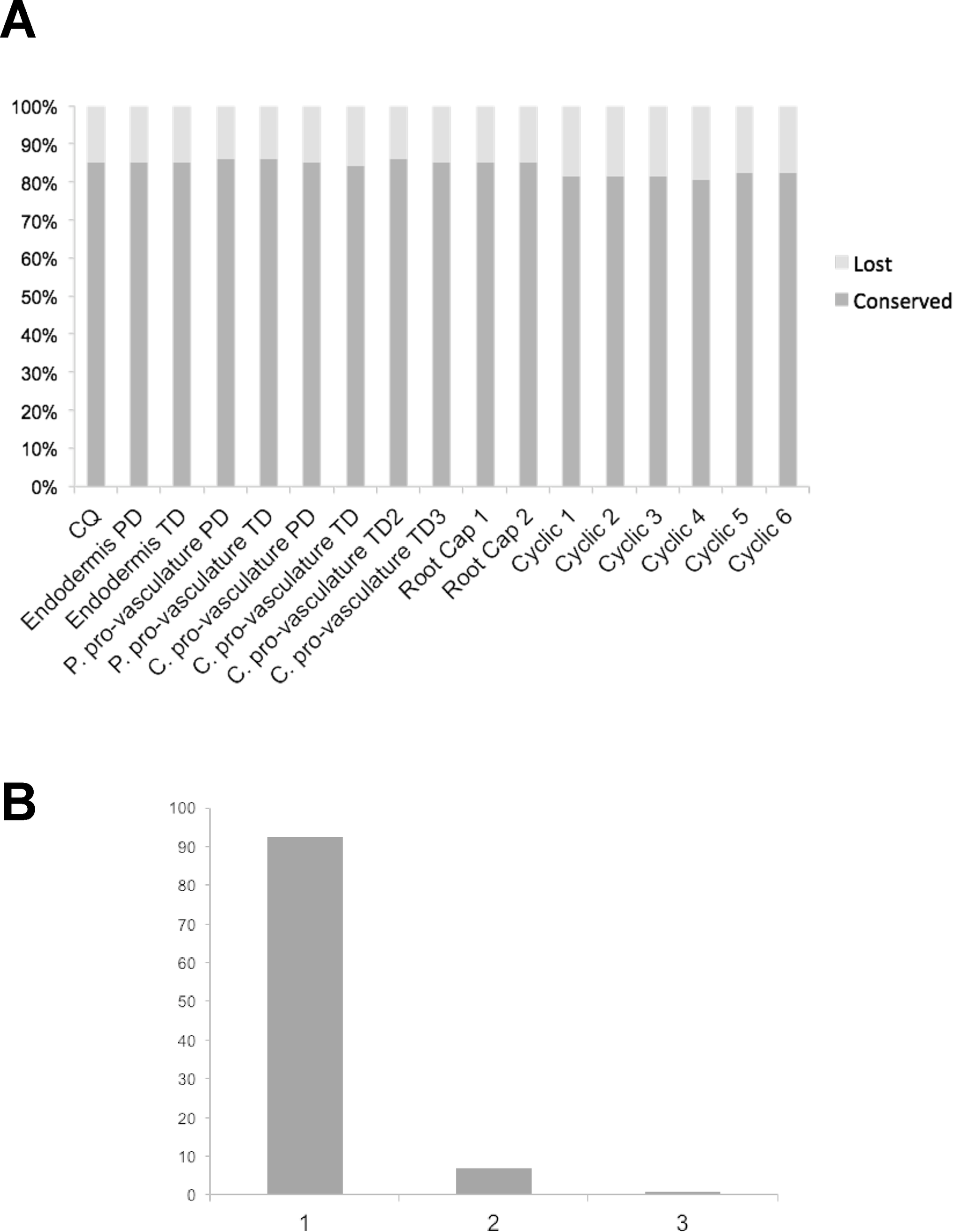

Supplement: S2 Fig — (A) The frequency at which each attractor was recovered or lost in the systematic perturbation of all Boolean functions is shown. (B) The graph shows the percentage of the simulations at which the new attractors are recovered. (TIF) [file pcbi.1005488.s002.tif]
