# Supplementary material for: A dynamic genetic-hormonal regulatory network model explains multiple cellular behaviors of the root apical meristem of Arabidopsis thaliana
Source: PLoS Comput Biol. 2017 Apr 20;13(4):e1005488. doi: 10.1371/journal.pcbi.1005488 (PMC5417714; doi:10.1371/journal.pcbi.1005488)
Supplement: S5 Appendix — The 11 attractors of the discrete GHRN1 model are maintained as steady states in the continuous extensions of the models. Moreover, the cyclic attractors and 100,000 random initial conditions converged only to one of these 11 steady states. The sets of random parameters used for the parameter analysis are also shown. (PDF) [file pcbi.1005488.s007.pdf]

S5 Appendix. Analysis of the continuous version of the GHRN1 model.

The 11 activity configurations of the fixed-point attractors recovered by Boolean networks are steady states in the continuous extension of the model.

For this analysis we used the same parameters for all the nodes of the model (*h=50* and *gamma=1*). We set as initial condition the activity configurations of the attractors recovered by the Boolean network in the synchronic updating scheme. The configuration at which the continuous model converges at time 20 is shown. The 11 fixed-point attractors are steady states in the continuous model. That is not the case for the cyclic attractors that instead converge to one of the 11 configurations.

GHRN1

Fixed-point attractors

**Initial condition**	QC	End. PD	P. Prov. PD	C. Prov. PD	End. TD	P. Prov. TD	C. Prov. TD1	C. Prov. TD2	C. Prov.TD3	RC-1	RC-2
**State at t=20**	QC	End. PD	P. Prov. PD	C. Prov. PD	End. TD	P. Prov. TD	C. Prov. TD1	C. Prov. TD2	C. Prov.TD3	RC-1	RC-2
**Steady state**	YES	YES	YES	YES	YES	YES	YES	YES	YES	YES	YES

Cyclic attractors (2 states)

CYC1.1	CYC1.1	CYC2.1	CYC2.2	CYC3.1	CYC3.2	CYC4.1	CYC4.2
C. Prov. TD2	C. Prov. TD2	C. Prov. TD3	C. Prov. TD3	C. Prov. TD1	P. Prov. TD	C. Prov. PD	C. Prov. PD
NO	NO	NO	NO	NO	NO	NO	NO

Cyclic attractors (3 states)

CYC5.1	CYC5.2	CYC5.3	CYC6.1	CYC6.2	CYC6.3
End. PD	End. PD	End. PD	QC	QC	QC
NO	NO	NO	NO	NO	NO

Configurations attained by solving the system from 100,000 random initial conditions.

For this analysis we used the same parameters for all the nodes of the model (*h=50* and *gamma=1*). The initial conditions for the continuous model were set at random, where each node acquires a value from the range 0-1. Then the system was solved until a steady state was attained. We repeated this process 100,000 and quantified that all initial conditions converge to one of the 11 attractors.

	QC	End. PD	End. TD	P. Prov. PD	P. Prov. TD	C. Prov. PD	C. Prov. TD1	C. Prov.TD2	C. Prov. TD3	RC-1	RC-2
**GHRN1**	2441	6109	4514	12057	6673	14051	8164	8667	14382	8772	14170

Parameter analysis.

To analyze the effect of the parameters on the behavior of the system, we used 100 sets of random parameters. In each set, the parameters h and γ of each node were chosen at random from the ranges 10-50 and 0.5-1, respectively. Then, we used 10,000 random initial conditions and solved each system. In all the initial conditions and for all sets, the system converged to one of the 11 attractors described before. Below we show the parameters used in this analysis in the following order: h_CK, h_ARR1, h_SHY2, h_AUXIAA, h_ARF, h_ARF10, h_ARF5, h_AUX, h_SCR, h_SHR, h_MIR166, h_PHB, h_JKD, h_MGP, h_WOX5, h_CLE40, γ_CK, γ_ARR1, γ_SHY2, γ_AUXIAA, γ_ARF, γ_ARF10, γ_ARF5, γ_AUX, γ_SCR, γ_SHR, γ_MIR166, γ_PHB, γ_JKD, γ_MGP, γ_WOX5 and γ_CLE40.

1)
35.18243 28.6212 44.53779 30.67355 20.53203 21.5288 20.21638 18.82405 42.47934 35.85558 16.73683 34.24395 48.5244 42.26199 25.17075 31.17151 0.9614 0.8411895 0.6466153 0.7888253 0.9918947 0.7240419 0.668054 0.9679306 0.6393961 0.9227553 0.5166659 0.5714269 0.7458636 0.6350214 0.6579597 0.6817154

2)
20.01719 39.99074 11.37025 18.28144 47.60679 26.52541 15.81833 23.77864 22.52936 31.42436 32.71808 36.55937 23.77938 46.99955 29.01594 37.32195 0.7462064 0.6996312 0.7847705 0.5129161 0.9859259 0.7672655 0.8410881 0.946502 0.8358661 0.91808 0.8569899 0.5668861 0.6109092 0.8932143 0.8549362 0.9930145

3)
18.75951 33.18944 29.83597 16.40844 37.02842 17.78696 17.79996 33.89898 45.5724 44.75287 44.35694 23.48663 27.27511 32.2263 35.90904 39.65081 0.6460875 0.5878816 0.9297603 0.5938902 0.8795962 0.5909027 0.7537036 0.543482 0.5859638 0.9194988 0.6449186 0.7482866 0.5670024 0.8657622 0.579609 0.9334399

4)
14.34083 48.58203 40.61339 18.80117 46.69692 25.43189 22.92779 41.32241 22.61342 27.25732 46.43786 35.76021 14.58285 12.36326 16.03979 45.29205 0.6299087 0.9665736 0.9927285 0.8590833 0.8701846 0.9266256 0.749806 0.9412201 0.9263272 0.5581392 0.5425456 0.5607782 0.6526288 0.6920228 0.7154674 0.8148421

5)
31.95822 15.56088 48.80095 38.44659 48.20482 49.07861 41.86762 22.50065 13.10007 13.41651 26.11791 21.83944 19.20107 36.59415 18.38128 39.97359 0.9529242 0.7558229 0.7167592 0.7832859 0.8142453 0.7582696 0.9096409 0.7369844 0.6889065 0.7278032 0.7732789 0.6965199 0.8917725 0.611107 0.6776457 0.8280123

6)
43.2794 15.12419 37.09577 22.57951 19.67569 11.79241 18.1931 43.11282 33.36871 17.32182 29.82405 45.6824 34.85694 44.66697 12.71785 24.70715 0.8256634 0.5733339 0.8138695 0.6854001 0.5369492 0.9397137 0.5629875 0.5354816 0.6312365 0.538762 0.5492169 0.8349916 0.5039048 0.7495775 0.8628041 0.7260145

7)

40.20914 48.29772 42.67389 28.41874 49.75187 29.24839 45.32013 40.60061 32.53622 22.60504 35.37551 23.53068 19.8829 38.52881 38.55523 43.97442 0.8204974 0.630504 0.7322665 0.6842383 0.5379529 0.6992966 0.7919748 0.8634291 0.5269967 0.6591687 0.7589333 0.7598392 0.7894468 0.9494256 0.7440405 0.6164288

8)

21.76807 26.04966 38.97237 19.52369 14.55747 33.98326 33.82804 26.94768 29.63008 10.14928 20.14039 18.12151 49.11798 37.73507 46.76513 17.91315 0.6596016 0.779651 0.8668145 0.5768046 0.8355703 0.9532039 0.9462308 0.5601806 0.7317228 0.6299699 0.6611069 0.7962159 0.924132 0.7960621 0.57481 0.6439831

9)

17.00815 38.48134 41.14312 22.09265 41.91067 18.61991 26.86649 28.7876 36.14881 37.88616 39.99856 46.30123 49.35537 38.61717 42.54893 47.62928 0.9410367 0.7611118 0.8327904 0.9486616 0.5801631 0.8648162 0.8595266 0.8165601 0.8730891 0.9366052 0.9236831 0.9614452 0.5474945 0.9329275 0.820807 0.5370286

10)

12.48878 48.89055 16.95956 23.88807 12.44636 34.56693 31.21684 18.29494 35.77172 28.57021 30.36387 14.79176 44.79181 30.46734 41.65196 15.86564 0.8221163 0.7455095 0.9000505 0.8872468 0.5454855 0.547684 0.6176571 0.5357204 0.8699646 0.5922733 0.7099736 0.5087991 0.5544934 0.6907496 0.8025944 0.7365129

11)

16.48732 23.02945 42.50293 44.20849 41.31275 20.21161 21.43926 43.98627 19.11331 15.93835 17.21495 25.15271 24.1123 31.63809 10.57406 41.46304 0.5819844 0.6297176 0.7754736 0.7492883 0.6314356 0.6350067 0.6468135 0.5230973 0.863567 0.6427461 0.5763568 0.5982723 0.756119 0.9483383 0.9906396 0.5979487

12)

35.48339 22.17954 29.72217 33.33656 22.52743 31.69087 44.57531 27.12995 21.69769 35.44539 13.9067 41.63339 49.68891 22.18119 22.53315 19.72276 0.6952034 0.808618 0.9214689 0.7641328 0.5950942 0.6053406 0.8132275 0.7968097 0.5692079 0.7451573 0.872075 0.502856 0.592572 0.8439453 0.9942816 0.7751608

13)

19.5769 29.2203 29.18452 26.65784 26.79229 29.07723 19.04742 13.86364 46.11451 26.49307 36.66266 43.92967 49.37217 37.90812 17.92021 11.60263 0.8656303 0.9072806 0.9998409 0.6650497 0.5229231 0.5360589 0.6181536 0.5055888 0.8151755 0.6984007 0.5869729 0.9787936 0.6266585 0.9769433 0.6892687 0.8954659

14)

42.27028 12.71073 33.13817 12.28096 31.56684 45.09306 25.71692 37.8349 18.49125 38.43119 13.44068 34.71678 20.67234 40.40132 15.65056 15.97723 0.8628472 0.7325399 0.5391634 0.8624014 0.5708989 0.7657706 0.7484157 0.5350452 0.8522483 0.916215 0.71997 0.6379 0.6355132 0.6530689 0.8767782 0.532846

15)

48.51145 40.11645 43.49584 14.03565 25.00109 25.27918 14.24368 34.28117 37.58598 21.30664 18.77905 48.80385 41.94899 29.75887 45.5549 20.27525 0.7478807 0.8644226 0.6518688 0.8665342 0.8069936 0.8520582 0.6076504 0.5482693 0.9866778 0.654561 0.6257155 0.822743 0.6432512 0.6473453 0.7946511 0.888991

16)

31.92717 29.31001 29.40506 25.13833 25.77699 19.7985 47.06028 10.32122 10.9852 14.26175 43.20497 47.05024 48.64126 43.0839 12.88891 45.4252 0.8064472 0.8687051 0.7170686 0.9918804 0.6154992 0.6953095 0.6430509 0.5000742 0.7263988 0.8740923 0.7731133 0.8475999 0.6892978 0.9124119 0.6841255 0.5894273

17)

43.91428 46.20529 44.27122 46.12744 18.16136 20.12707 23.88852 38.27538 42.70481 18.37025 21.09332 32.58484 17.05885 14.30755 31.7529 28.64293
0.9465055 0.8033721 0.8219211 0.7784622 0.52663 0.8086784 0.7920373 0.7592129 0.7194779 0.7306702 0.9266445 0.5281747 0.6119032 0.6190608
0.842057 0.7074496

18)

39.35849 11.20802 41.0165 49.92723 13.33145 31.71447 46.24296 33.50798 40.51416 43.24011 31.67195 16.79172 36.37782 31.3208 42.0236 46.06914
0.9352148 0.9315633 0.7233008 0.8218228 0.6340438 0.5528029 0.7435674 0.6059712 0.7492459 0.9945173 0.6907806 0.5527575 0.7482856 0.8267264
0.7785576 0.9384948

19)

23.16293 39.39918 34.77874 43.63641 45.15961 18.54358 22.13064 34.36033 17.37488 44.23962 36.4478 17.50118 47.68384 31.66546 49.76626 17.13941
0.5163387 0.5348142 0.7635253 0.9793648 0.8685249 0.5742571 0.7568239 0.799037 0.7045385 0.8377283 0.8434033 0.7405592 0.7243063 0.6384996
0.7116838 0.5765089

20)

23.42745 31.93936 15.0972 44.06519 43.7313 12.19698 43.52817 34.39004 10.48199 43.87804 35.2539 14.18857 38.16621 31.20202 28.97724 12.81815
0.7281325 0.5911312 0.5570694 0.7926214 0.5280663 0.6120668 0.5590911 0.6604162 0.802307 0.9550877 0.7511925 0.782038 0.8608069 0.7395649
0.5789804 0.6289711

21)

10.31541 21.45006 26.01367 13.86582 17.24129 46.33665 22.59011 14.12809 44.17791 33.52991 17.27045 20.42534 20.46116 16.90045 20.95993 49.48695
0.87438 0.981114 0.9647803 0.5587631 0.5452538 0.8009532 0.5539111 0.7860791 0.5626913 0.6763965 0.9533277 0.6119735 0.5343561 0.6050492
0.6925627 0.6567728

22)

15.87901 20.77366 47.14861 29.19565 49.13593 37.15981 36.58901 49.95608 34.30018 40.51924 14.24822 31.38351 44.169 20.71511 20.26348 33.70892
0.6379023 0.7145704 0.9681437 0.8402326 0.809757 0.7327735 0.5384226 0.6396841 0.5299451 0.6997986 0.6018512 0.6314908 0.7659295 0.9544196
0.8132296 0.8088744

23)

34.33545 17.29751 10.29133 34.21428 24.55859 35.57324 34.1265 49.28603 26.36496 24.59881 26.63741 47.6335 21.40095 33.0232 36.39998 19.77844
0.8796318 0.5106467 0.9618779 0.7273378 0.9727459 0.9522805 0.8973356 0.5036904 0.5427889 0.9468881 0.9373522 0.7898376 0.990784 0.6125169
0.785191 0.7824552

24)

39.45444 32.35454 40.74293 21.92498 21.82184 34.61209 36.79698 16.38207 20.94792 36.20996 32.6456 40.4592 11.08543 21.98861 15.39973 16.04231
0.5034924 0.5717861 0.9012014 0.906072 0.9941891 0.6174508 0.7006667 0.8867187 0.5979936 0.5467003 0.8324148 0.5034146 0.5786309 0.6920647
0.5322422 0.6629683

25)

28.91944 32.67366 14.49833 17.0294 38.84698 46.93139 17.71075 32.25764 17.83861 27.91766 14.30577 36.90912 29.74664 34.31866 48.56875 21.65921
0.837781 0.8002075 0.8523725 0.8097319 0.5355761 0.6987807 0.5274113 0.6815724 0.5788458 0.8611655 0.7340576 0.6955853 0.5828829 0.9473128
0.6240851 0.7816981

26)

32.55853 17.00856 10.45131 47.37466 47.10144 41.15581 31.93434 27.60595 43.73298 40.57057 29.32615 43.16544 29.07133 34.52667 14.69546 21.18392
0.6998416 0.6198975 0.6310687 0.5102565 0.7041348 0.5473704 0.6848349 0.8222241 0.6079771 0.5834364 0.6358637 0.7039027 0.6859501 0.9422843
0.915017 0.5893743

27)

29.09615 24.64019 42.8074 44.1741 26.97509 48.48514 47.79187 30.24825 27.9725 47.61993 43.26022 18.92497 44.5832 25.83713 38.56165 33.95153 0.6179525 0.9240806 0.8408901 0.6963126 0.7411133 0.9018407 0.9050799 0.5676423 0.6189207 0.7575992 0.7529372 0.6957422 0.8528282 0.6820776 0.711774 0.6982398

28)

45.24197 18.18453 11.51282 32.94463 27.2892 12.49598 35.43319 12.3955 32.43439 29.02536 25.22561 22.6998 24.1327 14.48224 42.89996 33.70468 0.5292615 0.7397917 0.6311789 0.7614225 0.8628373 0.5330567 0.9387081 0.9603022 0.7189768 0.7449184 0.5619638 0.8775639 0.5780033 0.7285832 0.5033565 0.7903794

29)

36.68889 19.09348 29.19913 37.04611 40.68297 14.64159 33.1725 46.11815 36.47857 42.89183 17.71883 21.70676 39.44981 26.80847 15.13733 44.75918 0.9366529 0.6533354 0.8840716 0.6302512 0.5115298 0.5981394 0.9242802 0.7217847 0.6780085 0.5246086 0.5625067 0.7947887 0.5968696 0.7999872 0.547262 0.7137631

30)

30.69315 10.98451 31.64514 49.78064 14.6176 42.47844 19.36043 49.69554 28.51993 29.35765 27.42696 36.98607 41.24112 15.86608 46.68091 38.69165 0.5969549 0.8895724 0.6207106 0.989636 0.9171115 0.7697206 0.9212682 0.5928987 0.6696632 0.824633 0.8928658 0.7040942 0.6124497 0.58668 0.7932112 0.6038931

31)

41.69654 41.60682 33.77603 33.33401 41.04694 48.58022 15.33327 19.25023 44.2129 41.21156 30.90688 34.72811 14.81924 17.8653 31.05669 46.91426 0.8799128 0.5203476 0.5324005 0.7396613 0.9293286 0.9463765 0.964022 0.8216423 0.5105678 0.9524812 0.5821778 0.7061397 0.7408783 0.6982072 0.7441569 0.8775775

32)

43.57567 26.7097 20.21492 21.24838 32.1742 12.29343 31.74239 29.05769 47.45154 41.13525 45.95812 30.80874 43.58532 32.20182 45.17253 43.77665 0.5463842 0.5212284 0.5447101 0.9083375 0.5709351 0.850792 0.8797922 0.6894945 0.9899805 0.7591153 0.6760336 0.9970837 0.7611135 0.6738918 0.5274039 0.9898772

33)

22.56992 13.61118 39.16057 35.96579 49.98916 26.70908 18.10712 39.40084 19.04816 27.41588 18.17707 11.95357 43.25532 14.71729 36.15458 29.36729 0.9545272 0.517822 0.5634322 0.9844408 0.8234098 0.7365675 0.7089997 0.7771373 0.6274908 0.9778638 0.7517249 0.809732 0.7195609 0.6290577 0.7643606 0.8486269

34)

33.06723 22.39515 21.50378 43.05726 43.85041 17.89191 42.79488 38.95424 40.21466 34.30559 13.22947 29.00456 28.27157 31.63203 25.61928 30.00284 0.5421506 0.5456042 0.5432533 0.5476501 0.7096473 0.6382301 0.8963025 0.6872203 0.9295789 0.7092901 0.9546197 0.5956792 0.7299279 0.6189994 0.5243904 0.6797865

35)

25.43854 25.05905 47.59008 25.57397 44.40533 14.57178 38.42892 19.71865 43.9335 47.64907 11.09484 23.63294 14.69892 22.64936 44.85033 42.01878 0.6739967 0.6901009 0.5808639 0.7631985 0.9174577 0.7582767 0.5438038 0.9707068 0.7169985 0.6538349 0.6866951 0.609216 0.9301835 0.7834597 0.636641 0.8086392

36)

37.63715 11.51369 47.42199 22.84497 14.03638 45.18709 42.78936 19.8462 16.42787 42.36511 46.68711 49.8091 33.86499 32.52714 29.80642 48.58371 0.9851504 0.9641759 0.5499594 0.5991525 0.8447177 0.9206261 0.6063167 0.7431371 0.5416787 0.8851883 0.907692 0.6202636 0.9143891 0.6658726 0.7227292 0.6779168

37)

44.80646 24.45895 12.53182 35.73239 48.95111 47.55037 28.87658 46.39875 45.18077 25.80867 15.9705 34.79525 48.32518 31.77455 38.70068 24.05832 0.6157995 0.6291629 0.7288714 0.8668596 0.5441407 0.603026 0.8700112 0.7509087 0.6610759 0.9184096 0.8868034 0.6457941 0.620978 0.6220995 0.8359644 0.9677592

38)

49.64135 17.49156 28.92171 20.88432 41.22596 45.44699 47.09192 12.16769 27.48341 49.81468 23.97739 13.32264 36.88104 36.8204 15.34956 27.58304 0.8714615 0.829081 0.973423 0.9007244 0.5959615 0.6700457 0.9915686 0.7323975 0.6507768 0.5224407 0.8408601 0.8383869 0.9282706 0.6257 0.8054186 0.6290499

39)

45.67376 18.97512 31.14117 16.95821 36.30369 46.08898 46.7928 21.49919 33.61261 47.87541 36.36612 13.80446 49.31388 15.62375 44.70939 34.17735 0.6954008 0.7614093 0.9825547 0.9875383 0.5255635 0.5330055 0.9108992 0.5074143 0.9747991 0.9673657 0.7514382 0.7616525 0.8400761 0.6047167 0.5117134 0.6859583

40)

25.72203 44.37491 16.24981 28.97331 34.29963 18.515 30.34625 17.42804 48.21389 45.25554 48.98339 10.77487 42.04175 10.70969 44.77062 40.58727 0.5001101 0.5337827 0.6718373 0.5015022 0.9501279 0.7576178 0.9882494 0.7906319 0.9228643 0.5393882 0.9987688 0.8227205 0.9203906 0.8216715 0.5626391 0.552829

41)

46.32534 38.02325 40.81893 12.43422 30.35174 35.12179 28.75193 19.77524 29.95599 41.07239 44.09234 13.76428 44.92924 41.90173 45.52788 29.53898 0.9405159 0.8411744 0.8093911 0.7473048 0.5683232 0.8639263 0.9334302 0.9090189 0.903439 0.7474415 0.5843988 0.5291347 0.8390295 0.8519313 0.6145645 0.6455631

42)

10.8501 39.60009 19.52996 27.89143 27.03274 34.2325 11.74859 25.78451 21.72289 25.87332 12.41422 22.20377 17.58133 28.89988 37.21646 27.01137 0.5189598 0.6901443 0.8727628 0.6640745 0.8577176 0.8992716 0.7867803 0.9982445 0.5293577 0.953045 0.8427371 0.5619164 0.7684893 0.5497498 0.9569482 0.5763176

43)

24.65574 33.09431 25.8283 48.92076 21.04504 13.22196 20.61993 10.42427 24.68796 30.34084 25.34318 10.20184 46.45989 11.48194 13.6913 23.42711 0.7883723 0.6805112 0.8539113 0.5978317 0.9021067 0.9881238 0.8959324 0.5807181 0.8751503 0.8060274 0.735152 0.7319688 0.84742 0.529042 0.7777825 0.7237484

44)

23.46495 45.04974 39.55655 12.98744 40.93292 40.16769 46.03151 36.82612 32.40217 14.93951 10.77371 26.18584 25.60819 29.97731 40.02018 16.07881 0.5068251 0.6817293 0.7007242 0.5771084 0.7569935 0.7909667 0.8483459 0.9926473 0.8863885 0.7228102 0.848625 0.5104098 0.7141019 0.7584565 0.9690328 0.8741569

45)

43.77338 12.37351 29.57354 41.57376 48.23374 25.4089 23.92182 33.32336 16.68948 25.30582 19.39225 28.97394 15.01964 26.76083 45.24768 13.09083 0.7163321 0.6485602 0.9255366 0.7374879 0.761533 0.9505493 0.6692588 0.8529484 0.8696847 0.8345232 0.5137495 0.8738778 0.6045304 0.7227231 0.6666329 0.9677536

46)

21.98356 29.14666 27.10755 34.73644 16.89032 22.05125 23.59049 30.13159 35.63194 36.02363 33.35128 23.99903 26.30136 39.03724 22.58769 25.123 0.8890319 0.8957035 0.9447892 0.8663603 0.7399045 0.9623251 0.9340667 0.9061968 0.6971836 0.7418118 0.9344095 0.94221 0.7808426 0.9061938 0.776998 0.8649347

47)

42.70454 39.62756 12.61152 29.42141 29.87595 34.79776 20.06714 32.45282 27.60482 28.78659 35.5518 25.53364 11.09322 17.04606 41.06001 10.08174 0.6385808 0.6127762 0.5633396 0.9546395 0.6014477 0.772882 0.8531962 0.9194832 0.9226063 0.8972413 0.8896321 0.9163588 0.6419127 0.9349738 0.5994953 0.8676803

48)

19.00429 48.23389 27.32916 16.78926 38.80673 42.51293 47.6025 11.90405 21.2618 39.53983 25.25343 41.73452 43.28487 22.69098 26.83318 40.56884 0.808943 0.842748 0.705724 0.9522958 0.8982594 0.7122285 0.6450586 0.516083 0.9980982 0.7276688 0.7476568 0.5931801 0.9204486 0.7573305 0.8689015 0.7949674

49)

32.20015 10.20273 33.60949 15.88733 44.49451 15.72617 49.53787 47.69723 22.72501 40.74966 23.47305 25.85224 33.19132 49.23048 40.21918 49.77214 0.5939938 0.5997929 0.5377015 0.6935363 0.5301852 0.9523379 0.5360594 0.7617296 0.6304053 0.6011599 0.5955315 0.9199999 0.8426006 0.8474486 0.5857581 0.7145504

50)

45.46326 16.38938 22.72763 29.13365 25.43196 20.28402 15.73568 11.24463 46.20182 14.74915 25.60857 25.12483 42.52536 15.72223 15.27336 24.04262 0.7712779 0.5295012 0.9937146 0.8796939 0.5212547 0.6559437 0.5560348 0.7330896 0.8154168 0.5276241 0.5879406 0.9149772 0.9188714 0.9721194 0.7339513 0.9508611

51)

31.62615 10.96891 27.69042 33.47123 18.23234 40.05195 12.41744 14.67001 15.82889 45.09024 17.05645 16.16747 36.97852 38.63727 15.59852 20.10983 0.6504916 0.7089724 0.8229262 0.6310513 0.7882857 0.5525987 0.5763814 0.6270679 0.8489454 0.7600364 0.8969075 0.8807863 0.7112181 0.7639243 0.7233768 0.8703575

52)

10.97702 33.63847 34.76043 40.56806 15.50572 14.24032 23.40574 10.7973 16.54329 42.9953 17.11938 37.0563 18.65165 49.18867 19.57312 15.05339 0.8212417 0.8131968 0.7800761 0.6741046 0.5158903 0.5172695 0.8677922 0.6463435 0.9515041 0.6356172 0.6540471 0.9961711 0.7517063 0.9302472 0.5685535 0.5125964

53)

45.59533 45.71696 13.97993 45.46715 32.93335 41.65231 35.73112 38.12992 43.45442 31.86684 41.2995 22.27812 15.85914 35.11109 12.7783 45.49219 0.938415 0.8522777 0.8386866 0.8018854 0.9170257 0.8941112 0.6717369 0.7125203 0.9387618 0.9916429 0.9379534 0.8237351 0.8900138 0.5307044 0.9329478 0.9908269

54)

27.86365 19.47052 34.13889 24.31977 33.97478 34.60055 35.66964 18.59739 42.03017 29.96403 24.72108 35.7416 41.84498 23.30954 30.9365 40.06213 0.710748 0.8084682 0.611016 0.9559867 0.5287481 0.6368121 0.6587196 0.8016531 0.8371873 0.5410962 0.6640732 0.9566357 0.6153959 0.8879091 0.632885 0.7192908

55)

41.38557 17.86346 42.12691 14.28698 41.56923 37.0636 39.5708 13.36031 38.50294 25.75067 35.36925 15.67737 32.43974 27.66388 11.89662 20.02133 0.9300838 0.8254951 0.8810919 0.908967 0.5335518 0.5446284 0.6983105 0.9647164 0.5752154 0.7484863 0.7634138 0.9459453 0.68709 0.644966 0.6157369 0.68026

56)

22.33459 49.22007 48.75206 39.74548 30.5447 23.01899 18.69579 39.16102 34.44087 36.35064 35.77361 44.93283 41.15768 35.47449 42.19724 45.9696 0.6068863 0.7663427 0.7502559 0.9429782 0.5466083 0.8816667 0.8023296 0.7183582 0.6925134 0.6910505 0.9976873 0.5905271 0.9343731 0.6209929 0.8356678 0.5449516

57)

38.95891 12.09071 15.35723 12.31162 20.1528 21.38242 34.95343 32.18533 41.7129 22.0943 38.895 16.18959 27.3322 34.04801 10.68914 13.61728
0.6444146 0.8860303 0.9269107 0.8653203 0.8683166 0.7898377 0.6729807 0.9607133 0.7197462 0.6441415 0.9054292 0.8525301 0.788612 0.6752718
0.8509953 0.9284108

58)

14.48674 23.88716 19.20878 29.8094 20.09294 20.23099 41.78088 10.40546 30.8238 32.31857 43.99394 35.8228 41.47194 37.86374 19.0275 17.70976
0.916258 0.6107158 0.645007 0.8506446 0.9484779 0.8880866 0.8867054 0.6311513 0.5580459 0.9129325 0.6029186 0.7409849 0.6901932 0.9652597
0.7959648 0.6373118

59)

33.64457 29.31408 19.46344 43.76551 20.79979 49.02736 13.69224 32.00745 35.73046 45.55489 29.54465 38.98718 36.23955 27.00011 42.66035 30.67797
0.8358571 0.5343474 0.5191531 0.5943568 0.9420822 0.9437486 0.9025337 0.6765161 0.6130107 0.7332942 0.6018678 0.5069044 0.7923247 0.5564422
0.6536994 0.8362975

60)

33.56377 23.65965 17.70912 33.49759 43.1277 28.30715 22.90947 19.84155 11.34465 28.23455 30.52902 16.38256 41.89557 20.66941 28.95777 19.01449
0.6980605 0.9398986 0.548557 0.9391797 0.5009817 0.5021708 0.6020806 0.7460189 0.5330024 0.8242258 0.7248903 0.9972132 0.8289537 0.7377999
0.8966688 0.5111331

61)

14.95631 47.76074 31.46377 35.93894 34.36487 19.99782 11.6707 40.83215 49.02522 41.07062 28.06187 21.19951 11.4114 23.02206 43.28309 25.20657
0.9293327 0.8311944 0.7144592 0.8015689 0.9850186 0.907868 0.567387 0.890277 0.6080734 0.7261409 0.7963781 0.7330215 0.6693459 0.5081449
0.928727 0.6284742

62)

23.29945 20.46298 35.90125 21.39728 19.25409 39.66887 32.24582 49.95919 43.47535 20.05433 33.07508 19.2476 16.99884 32.31112 10.02133 39.00501
0.5983248 0.8591125 0.6013176 0.6694389 0.6926818 0.5063505 0.9964611 0.8412932 0.511474 0.6954802 0.7559023 0.9060369 0.7749551 0.568588
0.5972864 0.5089012

63)

14.19403 17.88446 42.64334 11.78211 24.49072 44.56225 24.65111 19.78009 39.50296 38.76071 21.43013 39.23228 31.26163 13.68745 13.50654 34.80678
0.6646472 0.7019469 0.6826916 0.8750216 0.8461839 0.8703599 0.510159 0.992452 0.7665395 0.9871076 0.7348136 0.5847614 0.6172944 0.6361463
0.8604004 0.8472719

64)

13.18989 35.93352 10.0017 48.949 37.46558 29.02875 35.03868 41.4495 47.33017 14.5626 24.47055 30.51719 45.25914 38.64737 21.70359 47.82298
0.9601712 0.8481779 0.8771314 0.5411251 0.8187277 0.6698253 0.6681817 0.8040544 0.805625 0.5942327 0.6766322 0.6339689 0.9397223 0.5674927
0.9108136 0.6422806

65)

18.46227 22.04518 35.38509 42.2075 19.65526 19.52339 22.07781 23.00384 42.01188 25.82202 42.26981 46.1865 31.34664 33.63479 33.56905 47.67525
0.6477033 0.5894312 0.8065866 0.7805287 0.7166061 0.7284487 0.5491003 0.9212715 0.6753611 0.6157912 0.9433252 0.9129171 0.7793559 0.8049555
0.852555 0.5629459

66)

28.02118 22.38709 30.2268 40.92474 47.28207 29.31342 48.48022 21.21748 40.59817 48.30336 31.21986 44.56781 18.75001 17.53694 24.8441 21.91266
0.5588151 0.583006 0.55427 0.7338654 0.9337599 0.938645 0.5006813 0.6867496 0.7110509 0.6342557 0.9580724 0.5005488 0.9178474 0.708295 0.8168494
0.9110172

67)

41.20009 19.83303 16.44225 25.86317 16.89069 41.73823 44.65976 43.29722 33.1409 36.49275 17.27161 17.96662 48.3894 25.2772 43.06773 22.28565 0.5584845 0.8556211 0.647784 0.7790481 0.7391126 0.9725712 0.7404854 0.6521671 0.7281902 0.9843734 0.6359831 0.9441012 0.5145799 0.8849659 0.6874434 0.6226856

68)

12.8324 38.20142 44.68743 32.88178 14.30194 48.06028 26.114 43.91737 47.81455 13.03794 38.04272 38.98941 26.64259 36.8171 22.91095 34.31355 0.8303006 0.573624 0.5874341 0.622746 0.8298001 0.7467185 0.5207908 0.808588 0.8461822 0.6597296 0.7045528 0.6312619 0.6918906 0.8339281 0.5506002 0.5692638

69)

34.20765 47.54148 30.61078 12.62576 26.92123 49.61044 46.6902 30.27702 29.55573 13.36027 33.194 10.23519 33.62893 38.50974 43.83783 20.47272 0.9933965 0.6483887 0.7746624 0.8064619 0.5617858 0.5609 0.7651806 0.9359235 0.8775614 0.6591989 0.7614727 0.8306087 0.9781104 0.7870137 0.7544584 0.5732155

70)

31.09147 24.77158 14.82979 32.25253 38.32176 18.48395 33.88235 37.94392 37.54593 34.17089 47.46222 47.52436 22.75659 20.71005 12.83722 14.48747 0.961034 0.7462031 0.8440234 0.5051406 0.9612585 0.9783607 0.9154048 0.8413708 0.6281372 0.6999387 0.785524 0.8622084 0.7084827 0.5094538 0.5106794 0.9400888

71)

48.99336 33.88924 23.50031 49.32642 30.47614 15.22735 28.09595 16.24405 26.51989 42.94436 36.51586 34.14052 37.04501 21.39391 11.78879 21.02098 0.8720985 0.9979062 0.9145257 0.75957 0.9938427 0.9100342 0.8476164 0.961444 0.6165642 0.995177 0.9528986 0.587553 0.9284446 0.7694911 0.9940191 0.7572251

72)

44.6442 30.74043 21.48284 31.12699 27.43631 12.1086 47.19227 10.16625 43.17236 26.71591 37.41231 38.69491 33.74807 13.81237 41.80535 10.60466 0.6881838 0.9690476 0.9639191 0.8368572 0.8843502 0.6798803 0.6954236 0.9296065 0.9199959 0.7578736 0.5809862 0.5228993 0.9639691 0.9091847 0.5867151 0.8112913

73)

39.53778 30.64164 18.16805 26.99879 11.11941 29.55282 14.58698 44.34708 48.19249 37.51566 11.15026 44.49883 15.72036 46.0768 16.78914 11.56152 0.8247486 0.8804494 0.7714652 0.7361904 0.6163424 0.9384181 0.9384987 0.9632827 0.6537913 0.8898701 0.5766275 0.8048479 0.8986166 0.9936455 0.9975835 0.5262968

74)

43.66813 22.35592 34.06852 49.4202 30.98425 29.89198 48.98632 23.06597 35.32693 21.18214 19.6923 25.56017 23.99909 40.13301 21.70347 27.85597 0.5923379 0.5191873 0.5990467 0.530011 0.904569 0.5957587 0.9071293 0.7128293 0.9872388 0.96392 0.7637307 0.6049074 0.8719749 0.6914582 0.7308528 0.7089544

75)

45.73081 16.51729 14.42369 45.11296 16.61684 35.18085 31.20803 21.46094 37.13814 11.68319 29.61319 31.26625 41.8109 37.72905 32.65218 34.96846 0.7906353 0.5755643 0.7294998 0.6043127 0.896517 0.8709549 0.999733 0.5407895 0.9497057 0.7167165 0.9493671 0.7219869 0.5993263 0.8616675 0.721411 0.9992698

76)

39.62771 18.29782 13.46202 44.47188 27.05342 34.73404 29.13816 34.12636 11.95223 13.87534 38.95647 45.09287 13.35897 27.39295 21.88136 36.73024 0.8106428 0.6244406 0.5924138 0.9091637 0.6424703 0.5355684 0.7409962 0.6578866 0.8349951 0.8740377 0.7776393 0.9338566 0.6614109 0.6919965 0.9411592 0.6934011

77)

32.83569 49.51703 16.86276 10.28611 41.39498 28.36479 27.24735 44.53319 49.9422 46.49711 18.81813 15.69257 36.42051 22.36784 18.10888 49.18926 0.5788766 0.5922632 0.7013487 0.6389825 0.6041028 0.652779 0.9351361 0.7840155 0.6665438 0.794472 0.5053434 0.7163168 0.5148399 0.9033519 0.8534371 0.6578186

78)

45.6773 36.50561 26.17752 39.79184 10.77673 33.68723 46.70899 36.88902 39.30005 12.48897 46.52075 23.11974 46.94349 45.09705 41.27458 22.58599 0.9303233 0.6015923 0.6354021 0.9938365 0.5510896 0.5822926 0.9117434 0.6229696 0.7954773 0.8955147 0.7753816 0.6536831 0.855103 0.877019 0.8083427 0.7277549

79)

33.28007 34.02524 42.58812 47.80362 10.87164 15.53613 21.79624 27.95757 15.09605 36.79032 40.26597 35.53226 38.94188 31.22724 15.21807 31.08563 0.880489 0.6026287 0.8394545 0.9558195 0.8001777 0.5393113 0.6220245 0.5548256 0.532369 0.5926111 0.6853103 0.6684427 0.5957429 0.6861308 0.5808719 0.6227194

80)

24.88594 40.06758 42.81445 26.94265 22.04319 27.13176 49.95997 18.29802 36.02599 41.11442 18.57848 30.33692 47.59737 19.97357 26.56292 42.15693 0.8936152 0.5001447 0.504022 0.5781852 0.6336023 0.6221539 0.619489 0.8087569 0.8438856 0.8070063 0.8475395 0.956132 0.7585406 0.9163492 0.6275989 0.538804

81)

21.73712 39.05998 18.00186 12.62755 19.61786 49.52721 47.69489 13.82465 20.07425 28.85275 12.00437 15.05579 18.39772 17.35972 21.80428 37.31765 0.5281174 0.7054277 0.5894142 0.8070846 0.8307655 0.7079844 0.991033 0.7535971 0.7504437 0.9852602 0.6319258 0.5722691 0.9744839 0.5804519 0.9756096 0.7278759

82)

24.69064 11.76231 11.60029 15.89314 38.92823 16.489 15.33709 19.6177 45.01732 19.83421 22.33109 19.54979 42.03629 43.43625 37.52378 11.70835 0.8055727 0.9738897 0.7410384 0.7025335 0.9680164 0.6503298 0.7684316 0.8119928 0.6915098 0.9919755 0.5638197 0.9055487 0.5626034 0.7853825 0.8778949 0.8277998

83)

29.23953 13.95159 12.90612 40.39065 18.17768 43.09889 25.80323 10.67496 30.68981 16.00423 48.48891 23.44351 48.949 33.39013 19.73742 28.2046 0.9797092 0.6042214 0.6149909 0.9716255 0.8698723 0.6673339 0.6919421 0.9133411 0.7417997 0.7837492 0.5682154 0.8407648 0.7517504 0.6045736 0.6132459 0.5174655

84)

23.61476 35.97896 46.95533 43.60676 38.57718 19.24041 45.36738 47.76154 15.42241 15.06178 16.32255 21.94805 16.48614 15.48357 43.20305 21.83338 0.8764954 0.5541595 0.5188396 0.6097583 0.7318883 0.5710762 0.6210883 0.6371017 0.789048 0.8085191 0.8723245 0.7802298 0.6859617 0.776883 0.9114072 0.9384425

85)

20.80569 12.24175 37.27574 38.22861 22.02711 17.81892 37.06247 41.5267 43.95944 26.00486 10.52394 39.74778 46.32233 23.43847 37.42049 13.32625 0.8017726 0.9271426 0.8055842 0.9320893 0.507568 0.5769155 0.8668697 0.871326 0.9312947 0.9732875 0.5484967 0.9475269 0.7248584 0.8583845 0.6855841 0.6878722

86)

34.46375 12.75732 13.19058 39.77448 22.77931 27.51101 29.38822 27.40196 43.82184 45.80684 17.85182 39.22142 46.00263 46.82025 35.61245 38.88741 0.9658174 0.7185111 0.7702178 0.6230856 0.5863169 0.8349708 0.5011837 0.6508289 0.7348766 0.7649556 0.7896462 0.908511 0.5206057 0.5339878 0.9791959 0.5088752

87)

22.02121 15.57295 39.017 11.04874 48.58255 11.08378 49.21943 33.45262 27.21776 42.11992 29.70525 28.60678 14.07273 41.57773 47.27593 35.42989
0.6930436 0.5034031 0.7713193 0.7081679 0.9497308 0.9033936 0.7225437 0.8649358 0.6845772 0.5309748 0.5838889 0.5290151 0.6887021 0.9768636 0.891111 0.8193634
88)
47.93942 19.95421 49.37804 31.45366 38.48302 15.15744 45.7696 27.79987 25.61582 38.42762 24.13651 30.61643 16.86858 27.8292 21.5403 19.25341
0.6922416 0.8877331 0.7643371 0.8690181 0.7265501 0.9455225 0.7059179 0.6138086 0.6201813 0.5502948 0.7816646 0.564516 0.6516672 0.6138264 0.8838349 0.6707835
89)
48.34826 14.23604 19.58678 19.29241 15.91547 49.64374 49.94698 33.68265 20.82158 19.49838 16.17676 15.84135 16.91791 38.75961 21.02364 17.03849
0.6013798 0.7955575 0.5252727 0.9651614 0.5081467 0.8143938 0.7529831 0.9513974 0.6832206 0.6033037 0.6806769 0.5017675 0.6273551 0.5252021 0.6257447 0.5861797
90)
41.64985 12.67646 40.91333 12.24018 42.13297 48.65448 17.17655 45.31966 43.62873 20.98132 15.0483 38.63197 26.23171 15.00637 36.28612 45.06598
0.7208038 0.7849447 0.8047709 0.7386937 0.5937897 0.9011727 0.6423408 0.8914507 0.9974328 0.5942242 0.7889546 0.5256852 0.9067976 0.5678725 0.5319463 0.8856215
91)
20.94448 23.09214 38.51683 19.56391 14.65195 17.92215 11.71576 24.74888 44.07786 32.24045 31.92383 10.1818 34.91752 20.53147 47.76122 33.58187
0.6607273 0.6214373 0.6687161 0.9631277 0.8395401 0.8211499 0.8607046 0.8972484 0.9357146 0.6253743 0.5024015 0.9818217 0.7966638 0.512521 0.7773369 0.9309034
92)
46.11771 14.15867 25.8359 40.61885 14.33323 11.58391 42.17457 40.71652 39.12797 15.55372 40.01141 38.8174 21.83278 40.09581 49.18467 21.32637
0.7995046 0.7366958 0.9909869 0.5047077 0.7935583 0.723802 0.835041 0.8024961 0.9166678 0.6808453 0.6470696 0.5050953 0.9186737 0.6886347 0.5301979 0.7613789
93)
23.73005 13.30664 35.82293 15.45775 42.45664 20.03503 46.43248 23.63871 10.89884 45.50906 22.04927 15.49954 41.9011 38.65527 40.6581 34.51096
0.6160631 0.8427671 0.8148922 0.9897408 0.9627441 0.735618 0.7455328 0.8737906 0.5677363 0.611328 0.9398864 0.6480813 0.5251758 0.6952909 0.8660438 0.6405757
94)
40.60926 10.31494 15.29404 46.4552 38.17438 13.08448 29.40895 36.85665 38.60764 30.30766 26.47301 49.78764 49.05463 12.51767 36.22767 19.39854
0.8299003 0.7451764 0.5694132 0.9295002 0.5495949 0.9301672 0.6843868 0.9428409 0.5214461 0.5370027 0.538334 0.5365915 0.8859211 0.5797324 0.5017705 0.7545442
95)
44.60514 34.43405 37.92445 28.28608 36.41603 28.61288 15.94919 22.22 10.03928 19.2578 32.57958 23.11336 48.044 31.51694 44.67683 45.52019
0.5301643 0.8876788 0.8738286 0.6612895 0.9854071 0.5647075 0.7243827 0.6608067 0.5788682 0.7627433 0.5749942 0.7125587 0.949271 0.8179393 0.5224045 0.6790969
96)
33.4683 48.61941 16.07105 30.69203 27.48472 21.88528 18.23169 23.18451 19.07202 25.49618 49.88149 13.88264 18.37971 19.49013 27.36851 41.40207
0.7022017 0.56753 0.7151279 0.8958664 0.7710265 0.7458548 0.8898899 0.6731781 0.874035 0.7286102 0.981832 0.9342597 0.8761499 0.7351011 0.5031326 0.66993
97)

18.06151 28.39577 15.64172 29.82354 17.02843 42.2683 21.66516 41.36379 22.59656 21.86976 43.5401 36.98934 25.88661 35.78908 29.98062 14.98605 0.6360843 0.6396581 0.8297815 0.8574212 0.7266449 0.5697803 0.8798979 0.565424 0.5072787 0.6519644 0.6714361 0.9191798 0.8406985 0.6669738 0.6634784 0.5676936

98)

26.42723 32.19513 46.53243 46.00022 46.06598 46.39148 39.93575 21.87787 33.18636 30.43009 15.65138 36.02329 29.09727 11.31969 21.86084 40.6666 0.6916345 0.9147499 0.8821905 0.8410955 0.7727651 0.9392502 0.8142934 0.8307781 0.9364929 0.5363062 0.7798856 0.582419 0.6462302 0.5367534 0.7123471 0.6562562

99)

29.28777 39.78689 48.0875 46.28942 39.8318 42.99654 40.37657 19.8496 48.10849 42.69827 37.54421 47.29757 14.73773 28.25211 12.44071 32.28007 0.774107 0.8468665 0.625913 0.5223951 0.9934678 0.6362528 0.953808 0.5045094 0.9243488 0.7945948 0.8159357 0.7165684 0.7626661 0.8627953 0.5673495 0.9678962

100)

46.81757 39.07881 14.02086 39.39016 35.21909 33.1974 29.49629 48.35926 47.99635 49.20271 25.9767 29.58738 29.84103 30.44684 30.96084 13.54028 0.8324174 0.8770647 0.8721701 0.6824324 0.5541537 0.7429838 0.7565253 0.8288134 0.6885897 0.8186059 0.6696411 0.7798545 0.6650462 0.6719657 0.9315223 0.6692669
