# Supplementary material for: A dynamic genetic-hormonal regulatory network model explains multiple cellular behaviors of the root apical meristem of Arabidopsis thaliana
Source: PLoS Comput Biol. 2017 Apr 20;13(4):e1005488. doi: 10.1371/journal.pcbi.1005488 (PMC5417714; doi:10.1371/journal.pcbi.1005488)
Supplement: S7 Appendix — Tables 1 and 2 show the comparison for the GOF and LOF simulations, respectively. (PDF) [file pcbi.1005488.s009.pdf]

Table 1. Comparison of the GOF simulations with experimental evidence

GOF	*In silico* phenotype	Experimental phenotype	Recovery	Reference
CK	ARR1 is ectopically active in some of the attractors and SHY2 is ectopically active only in the pro-vascular TD attractors.	The expression of *SHY2* is enhanced after trans-zeatin treatment, but is still confined to the pro-vascular tissues of the TD. The size of the PD domain is reduced.	PR	[1,2]
ARR1	ARR1 is active in all attractors.	No such line has been analyzed in the RAM.	NC	--
SHY2	No QC, No ARF5 activity in the PD attractors.	A SHY2 GOF line has a smaller PD than wild-type plants. SHY2 represses ARF5 activity in the PD of the RAM.	PR	[2,3]
AUXIAA	No QC, No ARF activity in the PD attractors.	Treatment with auxin antagonists PEO-IAA reduces the size of the PD of RAM.	PR	[3]
ARF	No CK activity in the central pro-vascular TD 1 attractor.	Auxin has been reported to inhibit rapidly CK biosynthesis through its signaling pathway, consistent with the results of this simulation.	NC	[4]
ARF10	No QC	Roots have significantly less WOX5 expression as evaluated by quantitative PCR analysis.	R	[5]
ARF5	No peripheral (1) and central pro-vascular TD 1 attractors.	The overexpression of ARF5 in the RAM has not been reported, but ARF5 has been implicated in the regulation of proliferation at the RAM. The loss of various TD attractors agrees with this reported role of ARF5.	PR	[3]
AUX	None of the TD attractors, No Root Cap 1 attractor.	Roots treated with auxin have decreased levels of endocycling cells in the RAM, and more differentiation of the root cap.	PR	[5,6]
SCR	No Central and Peripheral attractors.	No such line has been analyzed in the RAM.	NC	--
SHR	No Root Cap attractors. The central pro-vascular TD 2 and TD 3 attractors are lost. The central pro-vascular TD1 attractor with the activity of the CK signaling pathway was still recovered.	SHR repression of CK levels is not involved in the regulation of proliferation/differentiation in the RAM. The result of the simulation is consistent with this as the central pro-vascular TD1 attractor was recovered correctly.	R	[7,8]
MIR166	No Central pro-vascular attractors.	An mir165 inducible line suppresses metaxylem formation.	R	[9]
PHB	No QC, Endodermis, Peripheral pro-vascular and Columella attractors. ARF10 is active in all the PD attractors of this simulation.	During embryonic development, restriction of PHB is necessary for WOX5 expression. In GOF PHB lines there is a decrease of WOX5 mRNA as analyzed by *in situ* experiments, ectopic metaxylem specification in the pro-vascular tissues, and defects in the specification of the pericycle and the cortex, accompanied by a reduction in the expression of JKD.	R	[9-12]
JKD	No Central and Peripheral pro-vascular attractors. ARF10 is not active in any attractor.	No such line has been analyzed in the RAM.	NC	--
MGP	No QC attractor. ARF5 is not active in any attractor.	No such line has been analyzed in the RAM.	NC	--
WOX5	No Central and Peripheral TD attractors, neither Root Cap and Endodermis attractors.	Roots have a severe delay in the differentiation of the columella and display reduced gravitropism.	PR	[13]
CLE40	No QC.	WOX5 expression dramatically decreases in *A. thaliana* roots treated with the CLE40 rice homologue.	R	[14,15]

Summary of the comparison between the recovered attractors in the GOF simulations with the experimental mutant phenotypes. R, recovered; PR, partially recovered; NC, not comparable (nonexistent mutant lines or chemical fields implicated).

Table 2. Comparison of the LOF simulations with experimental evidence

LOF	*In silico* phenotype	Experimental Phenotype	Recovery	Reference
CK	CK and its downstream effectors, ARR1 and SHY2, are not active in any attractor.	The PD domain is larger in roots where CK is degraded in the TD or in the pro-vascular. This is a multicellular phenotype that is not comparable with our single-cell simulation results.	NC	[1]
ARR1	ARR1 and SHY2 are not active in any attractor.	*arr1* mutants have longer meristems than wild-type plants. This is a multicellular phenotype that is not comparable with our results.	NC	[1]
SHY2	SHY2 is not active in some attractors.	*shy2-31* loss of function mutants have a larger PD of the RAM than wild-type roots. This is a multicellular phenotype not comparable with our results.	NC	[2]
AUXIAA	The components of CK signaling pathway are not active in any of the attractors, and the components of auxin signaling are constitutively active.	There is a high degree of AUXIAA redundancy in the RAM, making it impossible to compare this results with experimental data.	NC	--
ARF	CK and ARR1 are ectopically active in the central pro-vascular PD attractor.	There is a high degree of redundancy of the ARFs in the RAM, making it impossible to compare this results with experimental data.	NC	--
ARF10	WOX5 is ectopically active in the central and peripheral pro-vascular PD attractors.	Roots are agravitropic because columella cells do not differentiate correctly. WOX5 is still confined in its regular position, but QC25 expression domain is slightly expanded in a MIR160 overexpression line. Quantitative variations in auxin levels along the RAM might be reason this phenotype is not comparable with our results.	NC	[16,17]
ARF5	No QC attractor.	*mp* mutants do not express *WOX5* since embryonic development, and endoreduplication starts early in the RAM.	PR	[6,13]
AUX	No QC and no PD attractors.	Disruption of auxin signaling in the RAM anticipate endocycle onset.	PR	[6]
SCR	No QC, endodermis and peripheral pro-vascular (PD and TD) attractors.	The QC, the cortex/endodermis, the pro-vascular tissues are mis-specified in the *scr* mutant.	R	[11,13,18, 19]
SHR	Most of the attractors are lost, with the exception of two of the Central pro-vascular, and the Root Cap ones.	*shr* roots have defects in the QC specification, endodermis specification, and in the pro-vascular pattern. Ectopic metaxylem forms in place of protoxylem in the pro-vascular.	R	[11,13,20]
MIR166	No QC, peripheral pro-vascular and Root Cap attractors. ARF10 is ectopically active in the endodermis.	In *phb-mu* resistant line, PHB is not degraded by MIR165 and it is expressed ubiquitously in the RAM specifying all the pro-vascular cells as metaxylem. The loss of the QC cell type has not been described experimentally and constitutes a novel prediction of our model.	PR	[9]
PHB	No Central pro-vascular attractors.	In *phb* loss of function mutants there is ectopic specification of the protoxylem in the pro-vascular, and the synthesis of CK in the TD is compromised.	R	[9,11,21]
JKD	No QC or endodermis attractors. ARF10 is active in all the PD attractors.	*jkd* mutants show defects in QC specification. *JKD* and *BIB* are redundant in the RAM, and along with *MGP, NUC, SCR* and *SHR* are all necessary for the specification of the endodermis.	R	[22,23]
MGP	No Endodermis PD attractor.	*mgp* mutants show no defects. The auxin chemical field is involved in the differentiation of the QC and the endodermis [17], might be the reason this simulation result is not comparable with experimental data. Quantitative changes in auxin concentration make this phenotype not comparable with our results.	NC	[22]
WOX5	No QC attractor.	*wox5* mutants do not express several QC markers, and have a disorganized SCN with large cells at the QC position.	R	[13]
CLE40	CLE40 is not active in any attractor.	CLE40 mutants show delayed differentiation of the root cap.	R	[14]

Summary of the comparison of the recovered attractors in the LOF simulations with the experimental mutant phenotypes. Abbreviations are as in Table 1.

1. Ioio RD, Linhares FS, Scacchi E, Casamitjana-Martinez E, Heidstra R, Costantino P, Sabatini S. Cytokinins determine Arabidopsis root-meristem size by controlling cell differentiation. Current Biology. 2007 Apr 17;17(8):678-82.
2. Ioio RD, Nakamura K, Moubayidin L, Perilli S, Taniguchi M, Morita MT, Aoyama T, Costantino P, Sabatini S. A genetic framework for the control of cell division and differentiation in the root meristem. Science. 2008 Nov 28;322(5906):1380-4.
3. Scacchi E, Salinas P, Gujas B, Santuari L, Krogan N, Ragni L, Berleth T, Hardtke CS. Spatio-temporal sequence of cross-regulatory events in root meristem growth. Proceedings of the National Academy of Sciences. 2010 Dec 28;107(52):22734-9.
4. Nordström A, Tarkowski P, Tarkowska D, Norbaek R, Åstot C, Dolezal K, Sandberg G. Auxin regulation of cytokinin biosynthesis in Arabidopsis thaliana: a factor of potential importance for auxin–cytokinin-regulated development. Proceedings of the National Academy of Sciences of the United States of America. 2004 May 25;101(21):8039-44.
5. Ding Z, Friml J. Auxin regulates distal stem cell differentiation in Arabidopsis roots. Proceedings of the National Academy of Sciences. 2010 Jun 29;107(26):12046-51.
6. Ishida T, Adachi S, Yoshimura M, Shimizu K, Umeda M, Sugimoto K. Auxin modulates the transition from the mitotic cycle to the endocycle in Arabidopsis. Development. 2010 Jan 1;137(1):63-71.
7. Cui H, Hao Y, Kovtun M, Stolc V, Deng XW, Sakakibara H, Kojima M. Genome-wide direct target analysis reveals a role for SHORT-ROOT in root vascular patterning through cytokinin homeostasis. Plant Physiology. 2011 Nov 1;157(3):1221-31.
8. Hao Y, Cui H. SHORT-ROOT regulates vascular patterning, but not apical meristematic activity in the Arabidopsis root through cytokinin homeostasis. Plant signaling & behavior. 2012 Mar 1;7(3):314-7.
9. Miyashima S, Koi S, Hashimoto T, Nakajima K. Non-cell-autonomous microRNA165 acts in a dose-dependent manner to regulate multiple differentiation status in the Arabidopsis root. Development. 2011 Jun 1;138(11):2303-13.
10. Grigg SP, Galinha C, Kornet N, Canales C, Scheres B, Tsiantis M. Repression of apical homeobox genes is required for embryonic root development in Arabidopsis. Current Biology. 2009 Sep 15;19(17):1485-90.
11. Carlsbecker A, Lee JY, Roberts CJ, Dettmer J, Lehesranta S, Zhou J, Lindgren O, Moreno-Risueno MA, Vatén A, Thitamadee S, Campilho A. Cell signalling by microRNA165/6 directs gene dose-dependent root cell fate. Nature. 2010 May 20;465(7296):316-21.
12. Sebastian J, Ryu KH, Zhou J, Tarkowská D, Tarkowski P, Cho YH, Yoo SD, Kim ES, Lee JY. PHABULOSA controls the quiescent center-independent root meristem activities in Arabidopsis thaliana. PLoS Genet. 2015 Mar 2;11(3):e1004973.
13. Sarkar AK, Luijten M, Miyashima S, Lenhard M, Hashimoto T, Nakajima K, Scheres B, Heidstra R, Laux T. Conserved factors regulate signalling in Arabidopsis thaliana shoot and root stem cell organizers. Nature. 2007 Apr 12;446(7137):811-4.
14. Stahl Y, Wink RH, Ingram GC, Simon R. A signaling module controlling the stem cell niche in Arabidopsis root meristems. Current Biology. 2009 Jun 9;19(11):909-14.
15. Chu H, Liang W, Li J, Hong F, Wu Y, Wang L, Wang J, Wu P, Liu C, Zhang Q, Xu J. A CLE–WOX signalling module regulates root meristem maintenance and vascular tissue development in rice. Journal of experimental botany. 2013 Dec 1;64(17):5359-69.
16. Wang JW, Wang LJ, Mao YB, Cai WJ, Xue HW, Chen XY. Control of root cap formation by microRNA-targeted auxin response factors in Arabidopsis. The Plant Cell. 2005 Aug 1;17(8):2204-16.
17. Bennett T, van den Toorn A, Willemsen V, Scheres B. Precise control of plant stem cell activity through parallel regulatory inputs. Development. 2014 Nov 1;141(21):4055-64.
18. Sabatini S, Heidstra R, Wildwater M, Scheres B. SCARECROW is involved in positioning the stem cell niche in the Arabidopsis root meristem. Genes & development. 2003 Feb 1;17(3):354-8.
19. Di Laurenzio L, Wysocka-Diller J, Malamy JE, Pysh L, Helariutta Y, Freshour G, Hahn MG, Feldmann KA, Benfey PN. The SCARECROW gene regulates an asymmetric cell division that is

essential for generating the radial organization of the Arabidopsis root. Cell. 1996 Aug 9;86(3):423-33.

20. Helariutta Y, Fukaki H, Wysocka-Diller J, Nakajima K, Jung J, Sena G, Hauser MT, Benfey PN. The SHORT-ROOT gene controls radial patterning of the Arabidopsis root through radial signaling. Cell. 2000 May 26;101(5):555-67.
21. Ioio RD, Galinha C, Fletcher AG, Grigg SP, Molnar A, Willemsen V, Scheres B, Sabatini S, Baulcombe D, Maini PK, Tsiantis M. A PHABULOSA/cytokinin feedback loop controls root growth in Arabidopsis. Current Biology. 2012 Sep 25;22(18):1699-704.
22. Welch D, Hassan H, Blilou I, Immink R, Heidstra R, Scheres B. Arabidopsis JACKDAW and MAGPIE zinc finger proteins delimit asymmetric cell division and stabilize tissue boundaries by restricting SHORT-ROOT action. Genes & Development. 2007 Sep 1;21(17):2196-204.
23. Long Y, Smet W, Cruz-Ramírez A, Castelijns B, de Jonge W, Mähönen AP, Bouchet BP, Perez GS, Akhmanova A, Scheres B, Blilou I. Arabidopsis BIRD zinc finger proteins jointly stabilize tissue boundaries by confining the cell fate regulator SHORT-ROOT and contributing to fate specification. The Plant Cell. 2015 Apr 1;27(4):1185-99.
