# Supplementary material for: Correction: A dynamic genetic-hormonal regulatory network model explains multiple cellular behaviors of the root apical meristem of Arabidopsis thaliana
Source: PLoS Comput Biol. 2019 Jun 12;15(6):e1007140. doi: 10.1371/journal.pcbi.1007140 (PMC6561529; doi:10.1371/journal.pcbi.1007140)
Supplement: S1 File — (PDF) [file pcbi.1007140.s001.pdf]

PLOS COMPUTATIONAL BIOLOGY

RESEARCH ARTICLE

A dynamic genetic-hormonal regulatory network model explains multiple cellular behaviors of the root apical meristem of *Arabidopsis thaliana*

Mónica L. García-Gómez[1,2], Eugenio Azpeitia[3], Elena R. Álvarez-Buylla[1,2]*

1 Departamento de Ecología Funcional, Instituto de Ecología, Universidad Nacional Autónoma de México, Coyoacán, Ciudad de México, México, **2** Centro de Ciencias de la Complejidad, Universidad Nacional Autónoma de México, Coyoacán, Ciudad de México, México, **3** INRIA project-team Virtual Plants, joint with CIRAD and INRA, Montpellier, France

* eabuylla@gmail.com

Check for updates

OPEN ACCESS

Citation: García-Gómez ML, Azpeitia E, Álvarez-Buylla ER (2017) A dynamic genetic-hormonal regulatory network model explains multiple cellular behaviors of the root apical meristem of *Arabidopsis thaliana*. PLoS Comput Biol 13(4): e1005488. https://doi.org/10.1371/journal.pcbi.1005488

Editor: Rosangela Sozzani, Duke University, UNITED STATES

Received: August 26, 2016

Accepted: March 30, 2017

Published: April 20, 2017

Copyright: © 2017 García-Gómez et al. This is an open access article distributed under the terms of the Creative Commons Attribution License, which permits unrestricted use, distribution, and reproduction in any medium, provided the original author and source are credited.

Data Availability Statement: All relevant data are within the paper and its Supporting Information files.

Funding: This study was financed with the following grants: Consejo Nacional de Ciencia y Tecnología México (CONACYT www.conacyt.mx): 240180, 180380 and 152649; and Programa de Apoyo a Proyectos de Investigación e Innovación Tecnológica Universidad Nacional Autónoma de México (UNAM-DGAPA-PAPIIT http://dgapa.unam.

Abstract

The study of the concerted action of hormones and transcription factors is fundamental to understand cell differentiation and pattern formation during organ development. The root apical meristem of *Arabidopsis thaliana* is a useful model to address this. It has a stem cell niche near its tip conformed of a quiescent organizer and stem or initial cells around it, then a proliferation domain followed by a transition domain, where cells diminish division rate before transiting to the elongation zone; here, cells grow anisotropically prior to their final differentiation towards the plant base. A minimal model of the gene regulatory network that underlies cell-fate specification and patterning at the root stem cell niche was proposed before. In this study, we update and couple such network with both the auxin and cytokinin hormone signaling pathways to address how they collectively give rise to attractors that correspond to the genetic and hormonal activity profiles that are characteristic of different cell types along *A. thaliana* root apical meristem. We used a Boolean model of the genetic-hormonal regulatory network to integrate known and predicted regulatory interactions into alternative models. Our analyses show that, after adding some putative missing interactions, the model includes the necessary and sufficient components and regulatory interactions to recover attractors characteristic of the root cell types, including the auxin and cytokinin activity profiles that correlate with different cellular behaviors along the root apical meristem. Furthermore, the model predicts the existence of activity configurations that could correspond to the transition domain. The model also provides a possible explanation for apparently paradoxical cellular behaviors in the root meristem. For example, how auxin may induce and at the same time inhibit WOX5 expression. According to the model proposed here the hormonal regulation of WOX5 might depend on the cell type. Our results illustrate how non-linear multi-stable qualitative network models can aid at understanding how transcriptional regulators and hormonal signaling pathways are dynamically coupled and may underlie both the acquisition of cell fate and the emergence of hormonal activity profiles that arise during complex organ development.

mx/index.php/impulso-a-la-investigacion/papiit): IN203214, IN203814, IN211516proy. Mónica L García Gómez acknowledges fellowship 379717 from CONACYT for her doctoral studies in the "Programa de Doctorado en Ciencias Biomédicas de la Universidad Nacional Autónoma de México (UNAM)". The funders had no role in study design, data collection and analysis, decision to publish, or preparation of the manuscript.

Competing interests: The authors have declared that no competing interests exist.

Author summary

In multicellular development, signaling molecules are essential for the organization of cells into complex differentiated tissues. It is widely acknowledged that tissue or cell context is instructive for the specificity of cell behavior responses, but the underlying system-level mechanisms remain unresolved. The dynamic analysis of multi-stable regulatory network models grounded on experimental information allows the characterization of necessary and sufficient restrictions to recover the steady state gene/hormone configurations that correlate with different cell types or behaviors. Therefore, it is possible to formally understand how the cellular context, that mediates or biases particular regulatory interactions, is established during development. To this end, we proposed a minimal network model that integrates the regulatory cross-talk among the auxin and cytokinin signaling pathways with the main studied transcriptional regulators operating during the establishment and organization of the *A. thaliana* root apical meristem. We uncovered a regulatory network that represents a system-level mechanism that underlies the acquisition of characteristic activity configurations that correlate with different cell types, and at the same time mediates the readout of a hormone. Our model hence suggests that when a cell acquires a particular cell fate it may also acquire a differential capacity to respond to a particular hormone. This coupling mechanism between cell differentiation and the specificity in the responses to a hormone could be a general system-level mechanism operating in all multicellular eukaryote organisms. The systemic mechanism proposed here could hence contribute at understanding how signaling molecules and gene regulatory networks information processing operate during development. To achieve this understanding, the root meristem proved to be a very useful system.

Introduction

The root apical meristem (RAM) of *A. thaliana* is an important model for understanding the complex mechanisms underlying cell differentiation and morphogenesis during organ development of multicellular organisms [1–8]. The RAM of *A. thaliana* has a relatively simple cellular organization while it shares a general cellular structure and dynamics with stem cell niches (SCN) from both plants and animals [9,10], suggesting an underlying generic system-level mechanism that we may unravel by studying plant meristems, particularly the RAM [8]. In this study we build upon previous studies to further understand such mechanism in the RAM. We particularly aim at exploring how transcriptional regulation is integrated with the auxin and cytokinin (CK) hormonal pathways to regulate the cellular decisions regarding cell fate and behavior at the RAM.

The RAM comprises the SCN, the proliferation domain (PD) and the transition domain (TD) (Fig 1A). The SCN is at the tip of the RAM and is formed by the quiescent center (QC) cells surrounded by the so-called initial cells [11]. The QC cells are stem cells that have very low proliferation rates [12–15], while the initial cells are stem cells that divide at slightly higher rates and are specified as epidermis/lateral root cap, endodermis/cortex, pericycle/pro-vascular tissues and columella initial cells [11]. Upon division, the initial cells self-regenerate and produce a daughter cell that exits the SCN [16]. The progeny of the distal initial cells differentiate immediately into the root cap at the tip of the organ. In contrast, the progeny of the rest of the initial cells divide at higher rates in the PD towards the base of the plant. Eventually, these cells transit to the TD where they divide at slower rates and begin to endoreduplicate [17–19].

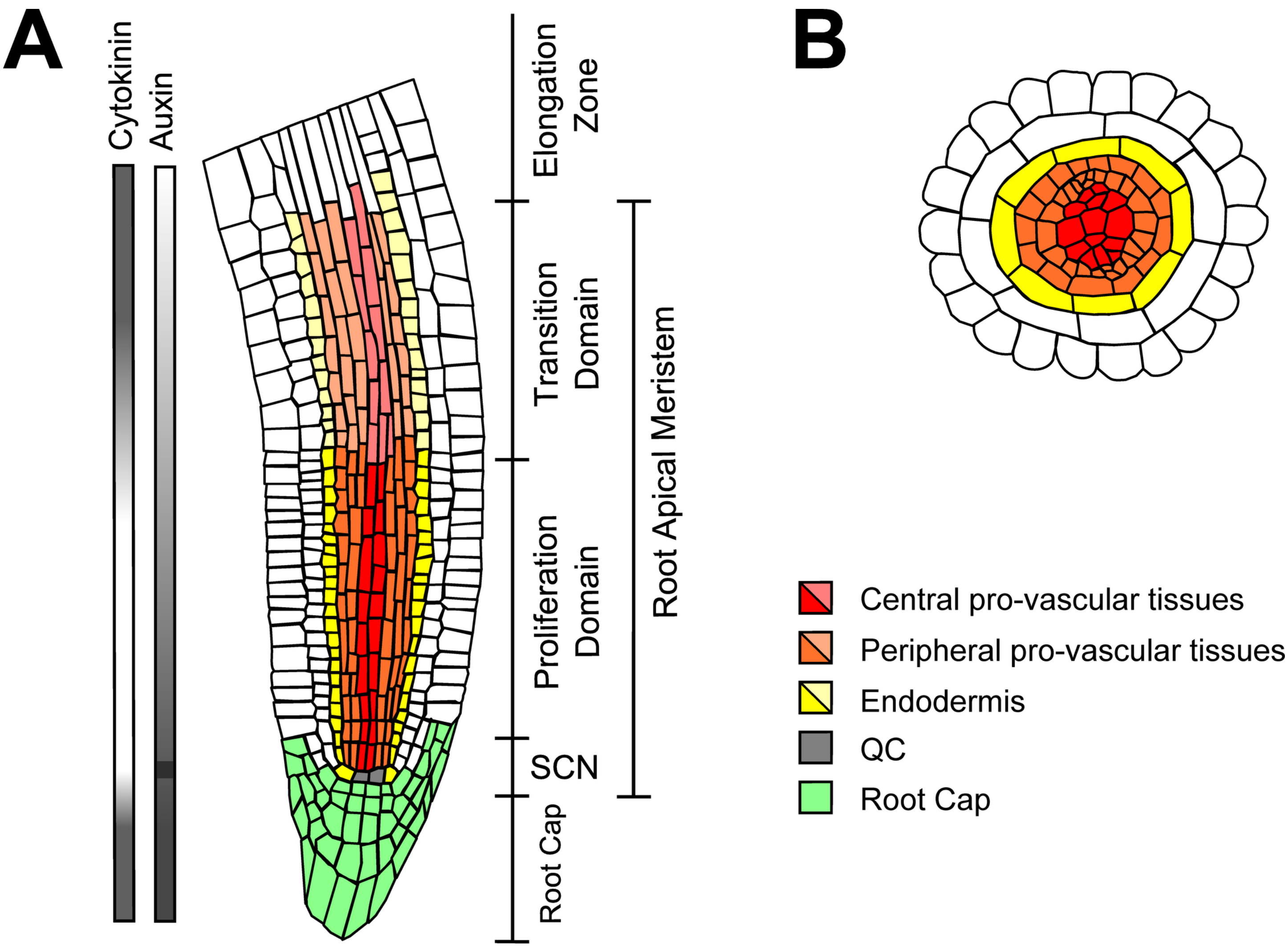

Fig 1. Schematic representation of the RAM of *A. thaliana*. (A) Longitudinal cross-section of the RAM. The different tissues of the RAM that we aimed to describe are indicated with different colors (bright for cells of the PD, and pale for cells of the TD). The distribution of auxin and CK along the longitudinal axis of the RAM is shown at the left. (B) Radial cross-section of the RAM showing the central pro-vascular tissues as the cellular domain that comprises the metaxylem and part of the procambium; and the peripheral pro-vascular tissues that includes the protoxylem, the phloem, part of the procambium and the pericycle. This drawing was made based on a confocal microscopy image of *A. thaliana* root tip.

https://doi.org/10.1371/journal.pcbi.1005488.g001

Afterwards, the cells leave the RAM, conform the Elongation Zone and finally the Differentiation Zone, where they acquire the morphological features of the differentiated tissues that constitute the radial structure of the root [18].

The cross-talk of hormone signaling and metabolism, coupled with the regulatory activity of transcription factors, microRNAs and mobile peptides involved in cell differentiation, is an important component of the system-level mechanisms underlying the organization and the maintenance of the RAM [8,20–22]. Experimental work has uncovered the function of some important genetic and hormonal components involved in RAM patterning [3,4,23–36]. While there are many regulatory mechanisms involved, the role of 1) the GRAS transcription factors and 2) the auxin and CK signaling pathways have been more thoroughly studied due to their importance defining the radial [30,37–39] and the apical-basal patterning of the RAM [23,26,40,41], respectively. High-throughput experiments coupled with bioinformatic analysis

have established the expression patterns of many genes in the RAM [42], have identified the regulatory targets of main regulators of the RAM [43–47], and have made it possible to infer the topology of global gene regulatory networks involved in RAM development [45–47]. But how such networks dynamically underlies the emergence of the expression patterns experimentally described, is not entirely understood. In this sense, systemic and dynamic approaches are recently starting to integrate available experimental data in order to postulate computational dynamical models of regulatory modules following a bottom-up approach, and provide integrative and formal frameworks to tackle the collective action of coupled hormonal and transcriptional regulatory mechanisms [1–4,7,48–51]. This integrative approach may lead to a systemic understanding of how documented patterns of expression emerge, and may also provide novel predictions that can be tested experimentally, leading to a recursive cycle between modeling and experiments. For instance, we previously proposed a minimal model of the gene regulatory network for SCN cell patterning [1,2]. This module integrates the activity of the GRAS transcription factors, the auxin signaling pathway and the *PLETHORA* genes, as well as a mobile peptide, among other key regulators of cell-fate in the root SCN.

Despite the comprehensive experimental and theoretical work done so far, some important questions regarding the genetic-hormonal regulation at the RAM remain unanswered. For instance, it is known that auxin and CK have opposite roles in the RAM as both act upon the cell cycle and alter cell behavior: auxin promotes cell proliferation at the PD [40], while CK promotes cell endoreduplication at the TD [35]. But the co-activity of both signaling pathways in the root cap where cells differentiate immediately [52–54] and the quiescence of the QC cells that have a maximum of auxin concentration [23,55], show that it is not possible to establish linear relations between hormone activity and cellular behavior at the RAM. Instead, another possibility is that the relative concentration of auxin and CK at the RAM or the cellular context is what underlies cell behavior along the root apical-basal axis. Here, we explore this possibility.

The distribution of auxin at the RAM (Fig 1A) emerge, in part, as a consequence of polar auxin transport mechanisms [7,48,49], but a more complete system-level understanding of how hormone signaling and metabolism act in conjunction with other hormones and genetic regulatory networks involved in cell differentiation is still poorly understood. In fact, multiple regulatory interactions between the auxin and CK pathways and the function of transcription factors have been uncovered. For example, a GRAS and a HD-ZIP III transcription factors are known to regulate the expression of enzymes involved in CK metabolism in the RAM [4,44] and of components of the auxin/CK signaling pathways [34,43,56,57]. Additionally, *WUSCHEL-RELATED HOMEOBOX-5* (*WOX5*), a key regulator of QC identity, promotes the accumulation of auxin [58,59]. In turn, auxin signaling both promotes and represses the expression of *WOX5* [27,31,58]. These opposite responses of *WOX5* to auxin could be explained in terms of auxin dosage; with auxin promoting the expression of *WOX5* at intermediate levels, and reducing it at either high or low auxin concentrations [60]. But we still do not understand how such correlations emerge. The complex relations between auxin and CK activity and cellular behavior could be emerging from the concerted action of several genetic and hormonal regulators, which show different activity profiles in different regions of the RAM. A systemic understanding of how the genetic expression configurations of different cells at the RAM are established from the joint activity of auxin/CK and such transcriptional regulators can be studied with the approach of dynamic discrete regulatory networks to uncover a core system-level mechanism that integrates experimentally grounded interactions [8].

In this study we used a Boolean dynamic network to propose a minimal model of the genetic-hormonal regulatory network (GHRN) that integrates the previously reported cross-talk between the auxin and CK signaling pathways, with transcriptional regulators that have been shown to be important in RAM patterning. Alternative GHRN models were proposed to

test the plausibility of two novel hypotheses concerning the regulatory mechanisms underlying cell-fate specification and context-dependent hormone responses at the RAM. Our results show that the hypothetical regulatory interactions we proposed are necessary in the context of the GHRN model to recover attractors with the activity profiles of the genetic components considered and the auxin/CK activity configurations observed for different cell types at the RAM and the root cap. The model presented here is useful to predict the existence of cell activity configurations at the TD that have not been characterized before, and to understand how cells might interpret and regulate auxin responses. Particularly, the model explains that the response of *WOX5* to auxin might be context-dependent. According to our results, the multistability of the GHRN underlies the emergence of different cellular contexts, each with specific auxin responses. This result allowed us to identify a potentially generic system-level mechanism to explain the cells' "competence" to respond to hormones as they acquire different fates. We assessed the robustness of the GHRN by making perturbations in its Boolean functions, and by making a continuous extension of the model. We also validated the GHRN model through the simulation of gain and loss of function mutations (GOF and LOF, respectively). Based on the mutant simulations we detected that one of the proposed hypothetical interactions provides a mechanistic explanation to several mutants that do not express *WOX5* and have a misspecification of the QC cells. We also identified the particular regulatory interaction between cell fate regulators and hormones that explains the co-activity of auxin/CK signaling in the root cap attractor. Therefore, this study extends our understanding of the system-level mechanisms underlying the emergence and maintenance of the cellular activity profiles at the RAM of *A*. *thaliana*. We achieve this with a newly uncovered minimal regulatory module with the necessary and sufficient set of components and interactions to recover the configurations of these, that have been documented experimentally. Additional components and hormone signaling pathways may now be added to the proposed framework in future modelling efforts.

Results

Reported regulatory interactions among transcriptional regulators and the auxin and cytokinin signaling pathways are insufficient to recover the RAM cell types/hormone activity profiles

To build the minimal GHRN of the RAM of *A*. *thaliana*, we extensively reviewed the available experimental information. We decided to include in the minimal GHRN model the genetic components and hormonal regulators that were well characterized at the functional level with detailed molecular genetic experimental studies in WT and various mutant conditions, and that their interactions with other important regulators of the RAM were well described and substantiated with various independent experimental approaches. Furthermore, we were particularly interested in adding solidly documented functional feedback regulatory loops to the network, because their combinations are crucial for the functioning and dynamics of the multi-stable system under study. Understanding this genetic-hormonal multi-stable network was our main aim in the present work. A vast number of putative regulatory interactions have been provided by genome-wide experiments in the context of the development of the RAM, based on various inference methods that generally consider the correlation on transcription levels of genes under various conditions or mutant backgrounds [43–47]. Such interactions could be explored in future extensions of the current model we are presenting in this paper. We summarize below the information used for the GHRN model and more details can be found in Table 1. The GRAS transcription factors SHORTROOT (SHR) and SCARECROW (SCR), and the BIRD transcription factors JACKDAW (JKD)/BALDIBIS(BIB), MAGPIE (MGP)/NUTCRACKER(NUC) among others, regulate the radial patterning of the RAM and

the specification of the QC cells [27,28,36–39,43,45,61,62]. The expression of SHR is regulated by many activators and repressors that in conjunction underlie its pattern of expression in the RAM. In conjunction they delimit its expression to the pro-vascular tissues [47]. SHR protein moves from its site of synthesis in the stele to the adjacent layer where it is retained by forming protein complexes with JKD/BIB and SCR [36,39]. These transcription factors regulate directly and indirectly each other expression. SHR, JKD and SCR are altogether necessary for the expression of SCR in the adjacent layer to the pro-vascular tissues [28,36,38,43,61]. SHR forms a protein complex with SCR that activates the expression of SCR [38,43,61], but it absolutely needs SCR and JKD to be fully located in the nucleus to do so [36]. Then, even though JKD is not a direct regulator of SCR, it is required for its effective expression. Similarly, JKD activation requires SCR and SHR presence [28], as both single mutants have a reduction in JKD expression. Interestingly, it has been shown that in the absence of SHR, JKD can repress the expression of SCR [45], indicating that JKD might have a dual role on SCR depending on the activity state of SHR. SHR and SCR are also necessary for the effective expression of MGP and NUC as their expression is reduced in *shr* and *scr* mutants [28,43]. JKD and BIB are jointly expressed in the ground tissue, the cortex/endodermis initials and the QC [36,63]; and MGP/NUC are highly expressed in the ground tissue and the cortex/endodermis initials [28,43]. The collective activity of all these transcriptional regulators, SHR, SCR, JKD, BIB, MGP and NUC is necessary for the specification of the endodermal cell fate in the RAM [36]. SHR, SCR and JKD are also necessary to specify the QC cells, as mutants in any of them have a misspecification of these cells [24,27,28]. SCR and SHR also promote the expression of the microRNAs MIR165a/6b that are expressed in the endodermis [30]. MIR165a/6b diffuse from its site of synthesis to the neighboring tissues where it promotes the degradation of the mRNA of the HD-ZIP III transcription factor PHABULOSA (PHB) [30], creating complementary MIR165a/6b and PHB activity domains that pattern the stele and the ground tissue of the RAM [30,64]. Low PHB levels are necessary to correctly establish the protoxylem, the pericycle and the ground tissue [[30,64], while high levels specify the metaxylem [30]. In the particular case of MIR166b, it is also expressed in the QC cells but its role in this context has not been conclusively established experimentally [30]. Moreover, it is interesting to notice that PHB feedbacks to the BIRD transcription factors by repressing the expression of JKD [64].

Parallel to the GRAS/BIRD/PHB/MIR165a/6b mechanism, the hormone auxin is an important regulator of cell behavior in the RAM [23,40]. Auxin promotes the degradation of the Aux/IAA proteins that otherwise bind to and repress the transcriptional activity of the ARFs (Auxin response factors) [65,66]. ARFs have been classified as activators or repressors of gene expression depending on their protein domains and the effects in the expression of auxin responsive genes [67]. ARF activators interact with a great variety of Aux/IAA proteins in comparison with the ARF repressors [68,69], but reports have shown that the interactions between the ARF repressors and the Aux/IAA proteins are necessary for certain auxin responses [70,71]. In the RAM, auxin distribution forms a gradient that correlates with the behavior of the cells: a concentration peak coincides with the position of the QC cells, intermediate levels with the PD and the root cap, and low levels with the position of the TD [23,52,55] (Fig 1A). On the other hand, CK responses are relatively high in both the TD and the root cap, as observed by the effect of the activity of CK transcriptional reporters [54,72], CK cell measurements of different cell types of the RAM [53] and local degradation of CK in the TD [26]. The activity of the CK signaling depends on a phosphorylation cascade to activate the ARR type-B transcription factors that regulate the expression of CK target genes [73]. In the TD, the ARR type-B regulators ARR1, ARR2 and ARR12 promote the expression of SHY2, an Aux/IAA protein that is key for the cross-talk between the Auxin and CK pathways in the transition from proliferation to endoreduplication at the TD of the RAM [29,35,74]. The expression of SHY2

Table 1. Regulatory interactions included in the GHRN models.

Interactions	Experimental Evidence	References
PHB → CK	*PHB* promotes the expression of two genes involved in the biosynthesis of CK, *ISOPENTENYL TRANSFERASE (IPT) 3* and *IPT7*.	[4,80]
SHR–| CK	SHR directly promotes the expression of *CKX1* that promotes the degradation of CK in the RAM.	[44]
SCR–| ARR1	SCR negatively regulates *ARR1* expression in the QC and the TD of the RAM.	[34,57]
ARF–| CK	The auxin signaling pathway rapidly inhibits CK biosynthesis in whole seedlings. Although some reports have shown that auxin promotes CK biosynthesis [29,81], the opposite has also been demonstrated [82,83]. Therefore, whole seedlings assays are likely to show the effect of auxin in most cells.	[78]
CK → ARR1	The CK signaling pathway activates the activity of type-B ARR transcriptional regulators, among them *ARR1*, *ARR12* and *ARR2*. In the model, *ARR1* is a representative of these three ARR proteins.	[73]
AUX–| AUXIAA<br>AUX–| SHY2	Auxin promotes the degradation of the Aux/IAA transcriptional repressors, among them *SHY2*.	[65,66]
ARR1 → SHY2	*ARR1*, *ARR2* and *ARR12* promote the expression of *SHY2*.	[29,35,74]
AUXIAA–| ARF<br>AUXIAA–| ARF5<br>SHY2 –| ARF5<br>AUXIAA–| ARF10	The Aux/IAA proteins dimerize with the ARF transcription factors and compromise their ability to regulate gene expression. Many Aux/IAA proteins physically interact with ARF5, including SHY2. SHY2 does not interact with ARF10, but IAA5, IAA17, IAA26, IAA32 and IAA33 do so. These Aux/IAA proteins are expressed in various RAM tissues.	[42,68,77,84,85]
JKD–| ARF10	JKD has been reported to bind the promoter of ARF10, but the effect of this interaction is unknown [45]. We hypothesized that JKD is a repressor of ARF10. This hypothesis was tested in the GHRN1 model.	—
SHR–| ARF10<br>SHR–| ARF5	A bioinformatic analysis of *SHR* regulatory targets predicted *ARF10* and *ARF5* as genes repressed indirectly by SHR.	[43]
MGP–| ARF5	This interaction constitutes a hypothesis tested in the GHRN1 model.	—
AUX → AUX	The polar transport of auxin forms a transport network whose activity underlies a dynamic steady state of auxin distribution in the RAM. To model this non-cellular autonomous role of auxin we included this positive self-regulation, to represent that a cell at a certain position within the RAM would have a constant auxin concentration.	[48,86]
WOX5 → AUX	*WOX5* promotes the expression of the auxin biosynthetic enzyme *YUC1*, and represses the auxin conjugation pathway.	[58,59]
SHR → SCR<br>SCR → SCR	ChIP-PCR analysis demonstrated that *SCR* and *SHR* bind to the promoter of *SCR*. The expression of *SCR* is reduced in *shr* mutants, most notably around the stem cell niche.	[38,43,61]
JKD → SCR	*JKD* is necessary for the nuclear retention of *SHR*, which is necessary for SHR to be able to activate *SCR* expression. Moreover, it has been shown that SCR and SHR by themselves cannot effectively activate SCR, unless JKD is present [36]. This is evident in the lack of SCR activity in the QC cells in *jkd* mutants [28].	[28,36,62]
JKD -| SCR	The amplification of JKD expression in *shr* mutant plants, showed that JKD can repress SCR in the absence of SHR. Thus, there seems to be a multi-stability in the role of JKD over SCR that depends on the state of other regulators.	[45]
SHR → SHR	The expression of *SHR* is not regulated by any of the genes included in the GHRN models. We assumed that if *SHR* is active at the beginning of the simulation, it will remain active henceforth.	—
SCR-> SHR<br>JKD -> SHR	*SCR* and *JKD* promote the nuclear retention of SHR. As SHR protein moves between cells, it might be found outside its expression domain wherever these regulators are present.	[36,61]
SHR → MIR166<br>SCR → MIR166	*SHR* and *SCR* promote the expression of *microRNA165a/6b* in the endodermis; the expression of *microRNA166b* in the QC is reduced in the *shr* mutant background.	[30]
MIR166 –| PHB	*microRNA165a/6b* post-transcriptionally promotes the degradation of *PHB* transcript.	[30]
PHB–| MIR166	In computational simulations, the mutual degradation between MIR165/6 and PHB create sharp boundaries between the MIR165/6 and PHB activity domains.	[50]
CK-|MIR166	Cytokinin treatment strongly represses the expression of MIR165 in the RAM.	[4,79]
PHB–| JKD	The expression of *JKD* is reduced in the *phb-1d* gain of function mutant.	[64]
SHR → JKD<br>SCR → JKD	The expression of *JKD* is reduced post-embryonically in *scr* and *shr* mutants.	[28]
SCR → MGP<br>SHR → MGP	RT-PCR and in situ hybridization analyses indicated that *MGP* expression is diminished in loss of function mutants *scr* and *shr*. *SCR* and *SHR* bind directly to *MGP* promoter.	[28,61]
ARF10 –| WOX5	*ARF10/16* are necessary for auxin-dependent repression of *WOX5*, to promote the differentiation of distal initial cells.	[31]
ARF5 → WOX5	During embryonic development, *WOX5* is not expressed in *arf5* loss of function mutants suggesting a dependence of ARF5 for WOX5 activity. Post-embryonically ARF5 or other ARFs could be mediating the regulation of *WOX5* expression. For example, *ARF6* is phylogenetically close to *ARF5* [82] and its expression is ubiquitous in the RAM [87]. Nevertheless, the fact that Abscisic acid requires *ARF5* and *WOX5* to promote the quiescence of the QC post-embryonically [88], suggests a regulatory link among them.	[27]

(*Continued*)

Table 1. (*Continued*)

Interactions	Experimental Evidence	References
CLE40 -| WOX5	*CLE40* treatment represses *WOX5* expression.	[75,76]
SCR → WOX5<br>SHR → WOX5	The expression of WOX5 is undetected in the *scr* and *shr* single mutants. These hypotheses were only included in the GHRN model.	[27]
WOX5 –| MGP	Prediction based on the complementary expression patterns of *WOX5* and *MGP* in the adjacent layer to the pro-vascular tissues in the RAM.	[1]
SHR–| CLE40	Prediction based on the complementary expression patterns of *SHR* and *CLE40*.	[2]

For each regulatory interaction included in the GHRN models, the experimental evidence and references are indicated.

https://doi.org/10.1371/journal.pcbi.1005488.t001

is particularly high in the pro-vascular tissues of the TD [29]. WOX5 is a transcription factor fundamental for QC identity, and it is widely acknowledged that is regulated by two parallel pathways (i.e., the GRAS transcription factors and auxin signaling [24,27]). SHR and SCR are necessary for WOX5 expression, while auxin promotes the expression of WOX5 through the ARF activator ARF5 (MONOPTEROS) and represses it through the ARF repressor ARF10 [27,31]. Additionally, the expression of WOX5 is negatively regulated by the mobile peptide CLE40 [75,76]. ARF5 activity in the PD is important to maintain cell proliferation [40,77]. Multiple regulatory interactions have been reported among the mentioned regulators: SCR represses the expression of ARR1 [34,57], WOX5 promotes the accumulation of auxin [58,59], auxin signaling and SHR promote the degradation of CK [44,78], CK strongly represses the expression of MIR165a/6b [4,79], PHB promotes the biosynthesis of CK [4] and both ARF10 and ARF5 were predicted to be repressed by SHR in a bioinformatic study [43].

We integrated this reported evidence of the regulatory mechanisms that underlie cell patterning in the RAM into a minimal GHRN model (Fig 2A). From hereon when we refer to hormones we mean auxin and CK that are the ones included in the GHRN model. It has been shown that JKD and BIB, as well as MGP and NUC have the same expression patterns and are putatively redundant in the RAM [36,43]. Thus, we decided to model the role of these regulators with the representative nodes JKD and MGP, respectively. Because there are 23 ARFs and 29 Aux/IAA proteins in *A*. *thaliana*, but there is only specific data about the role of SHY2, ARF5 and ARF10 in the RAM, these genes were modeled independently while all other ARFs and Aux/IAAs were included in a single ARF and AUXIAA node, respectively. Regarding the role of the type-B ARR regulators of CK signaling, it is known that ARR1, ARR2 and ARR12 redundantly promote the expression of SHY2 [29,35,74], and we decided to model their role with the representative node ARR1. Finally, we used MIR166 as a generic node to model the role of MIR165a/6b, as MIR166b is expressed in a broader domain than MIR165a [30]. The resulting network comprises the role of up to date experimental information about the hormonal and genetic regulation of cell fate and cellular behavior in the RAM. The model has two levels of complexity: the first one is revealed by the overall structure of direct/indirect interactions among the components of the network (Fig 2), and the second one consists of the formalization of the experimental information in the form of logical rules or tables of truth that describe how the activity of each node changes depending on the state of its regulators the previous time step. In the model the activity of the genetic components considers regulation at the transcriptional, post-transcriptional and protein activity levels; for hormones this entails metabolic regulation (biosynthesis and degradation). For instance, SCR is a clear example of how we formulated the logical rule of a genetic component that is regulated at different scales (see Model Assumptions). Briefly, its transcription is positively regulated by SHR and SCR [38,43,61]; JKD does not participate directly in the regulation of SCR but instead regulates the

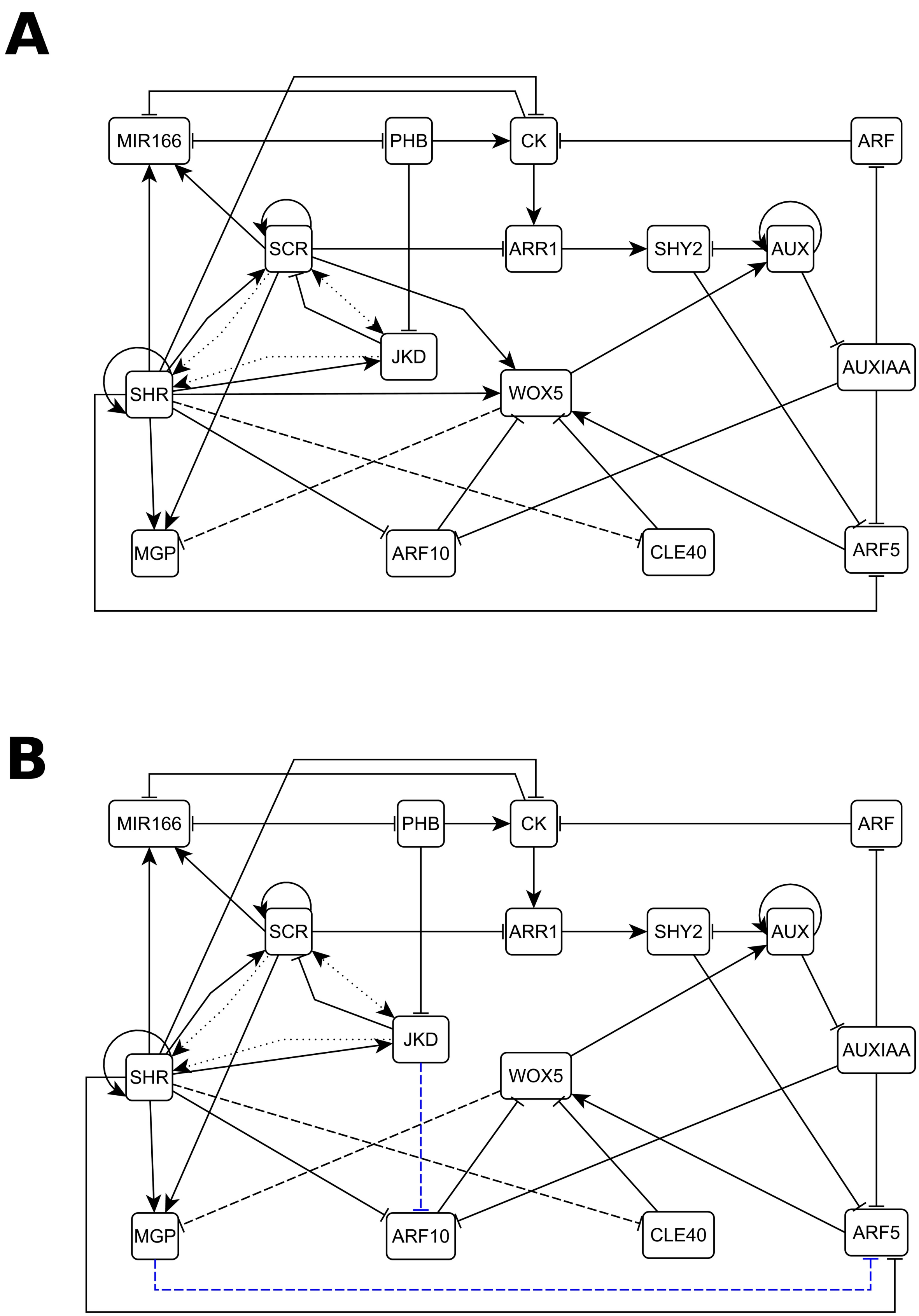
A
MIR166
PHB
CK
ARF
SCR
ARR1
SHY2
AUX
JKD
SHR
WOX5
AUXIAA
MGP
ARF10
CLE40
ARF5
B
MIR166
PHB
CK
ARF
SCR
ARR1
SHY2
AUX
JKD
AUXIAA
SHR
WOX5
MGP
ARF10
CLE40
ARF5

Fig 2. The genetic-hormonal regulatory networks of the RAM of *A. thaliana*. Topologies of the (A) GHRN and (B) GHRN1 models. Activating regulatory interactions are represented with directed arrows and inhibitions with blunt arrows. The interactions that represent regulation of protein movement are indicated with dotted lines. Hypothetical interactions are shown with dashed lines; the blue interactions are the hypotheses proposed in this paper.

https://doi.org/10.1371/journal.pcbi.1005488.g002

cellular localization of SHR [36], making JKD's activity an indirect but necessary condition for SCR activity. This conditional dependency made us include JKD as an activator in the logical rule of SCR, along with SCR and SHR. Hence, we are not explicitly considering the detailed biochemical mechanisms involved, but the overall structure and logic of the documented regulatory interactions are captured in the logical rules. The assumptions of the model are listed in the Methods (including two representative examples of how experimental information was formalized in the logical functions) and the logical functions of the nodes can be found in S1 Appendix. Given the regulators included in the GHRN and based on expression data from the literature, we expected to recover at least 8 attractors combining gene/protein activities and hormone presence, corresponding to the following cell types of the RAM: the QC, endodermis PD and TD, peripheral pro-vascular PD and TD, central pro-vascular PD and TD, and the root cap (Fig 3A). In this context, a cell type is formalized as the activity configuration of the components considered in the model, that have been experimentally documented to correlate with different cells in the RAM. Notice that for the central pro-vascular TD and root cap attractors the value of some nodes (indicated with * in Fig 3A) could be either 1 or 0. Hence, in these cases more than one attractor could represent the expected cell types. For the rest of the attractors we expected to find a unique attractor.

With the logical functions we proposed, based on experimental information, we solved the system to study the dynamic and concerted action of the regulators considered. The GHRN model that only includes experimentally reported interactions did not recover the expected activity profiles of the cells of the RAM. Based on the complementary expression patterns of CLE40 and SHR, and of WOX5 and MGP, repressive interactions were previously proposed between each pair of regulators [1,2] (Table 1). The GHRN model with these hypothetical interactions still did not recover attractors with configurations that have been documented for different cell types at the RAM (S2 Appendix). This is because WOX5 was not active in any attractor, so the model did not recover an attractor corresponding to the QC (S2 Appendix). Moreover, ARF5 and ARF10 activities do not match what is observed experimentally: the attractors corresponding to the central and peripheral pro-vascular PD do not have ARF5 and ARF10 activity contrary with expression data (Fig 4; S1 Fig; S2 Appendix). Therefore, the attractors recovered by the GHRN model are incorrect. To further verify that the known and previously proposed interactions are insufficient to describe the activity configurations of the cells of the RAM, we explored, systematically and exhaustively, if there exist any network that can recover a predefined set of attractors (Fig 3A) given the set of regulatory interactions (Table 1) included in the GHRN model (Methods) [91–92]. Interestingly, we did not find any network that could recover the expected attractors, indicating that it is not possible to obtain the genetic and hormonal activity profiles experimentally described for the cells at the RAM of *A. thaliana* with the regulatory interactions that we integrated in the GHRN model.

A GHRN model with novel predicted regulatory interactions recovers the observed hormone and transcription factor configurations of the RAM cells

The inability of the GHRN model to recover the correct activity configurations described for the cells of the RAM, particularly the QC attractor and the activity profiles of ARF5 and

A

Expected Attractors	CLE40	WOX5	SHR	SCR	MGP	JKD	MIR166	PHB	AUXIN	ARF10	ARF5	ARF	AUXIAA	SHY2	ARR1	CK
QC	0	1	1	1	0	1	1	0	1	0	1	1	0	0	0	0
Endodermis PD	0	0	1	1	1	1	1	0	1	0	0	1	0	0	0	0
P. pro-vascular PD	0	0	1	0	0	0	1	0	1	1	1	1	0	0	0	0
C. pro-vascular PD	0	0	1	0	0	0	0	1	1	1	1	1	0	0	0	0
Endodermis TD	0	0	1	1	1	1	1	0	0	0	0	0	1	0	0	0
P. pro-vascular TD	0	0	1	0	0	0	1	0	0	0	0	0	1	0	0	0
C. pro-vascular TD	*	0	*	0	0	0	0	1	0	0	0	0	1	1	1	1
Root cap	1	0	0	0	0	0	1	0	*	*	*	*	*	*	*	1

B

Recovered Attractors	CLE40	WOX5	SHR	SCR	MGP	JKD	MIR166	PHB	AUXIN	ARF10	ARF5	ARF	AUXIAA	SHY2	ARR1	CK
QC	0	1	1	1	0	1	1	0	1	0	1	1	0	0	0	0
Endodermis PD	0	0	1	1	1	1	1	0	1	0	0	1	0	0	0	0
P. provascular PD	0	0	1	0	0	0	1	0	1	1	1	1	0	0	0	0
C. provascular PD	0	0	1	0	0	0	0	1	1	1	1	1	0	0	0	0
Endodermis TD	0	0	1	1	1	1	1	0	0	0	0	0	1	0	0	0
P. provascular TD	0	0	1	0	0	0	1	0	0	0	0	0	1	0	0	0
C. provascular TD 1	0	0	1	0	0	0	0	1	0	0	0	0	1	1	1	1
C. pro-vascular TD 2	1	0	0	0	0	0	0	1	0	0	0	0	1	1	1	1
C. pro-vascular TD 3	1	0	0	0	0	0	0	1	1	1	1	1	0	0	1	1
Root cap 1	1	0	0	0	0	0	1	0	0	0	0	0	1	1	1	1
Root cap 2	1	0	0	0	0	0	1	0	1	1	1	1	0	0	1	1

Fig 3. Expected and recovered attractors of the cells at the RAM. (A) Expected attractors. Each activity configuration corresponds to the characteristic genetic expression and hormonal activity profiles of the following cells within the RAM: QC [23,24,27,28,30,36,38,55,63,87,89], Endodermis [28,36,37,39,63,87,89], Peripheral pro-vascular tissues [30,38,64], Central pro-vascular tissues [4,26,29,30,38,42,75,90] and Root Cap [7,31,52–55,75]. The color code is as in Fig 1. Asterisks indicate that the activity of a node can be either 1 or 0. (B) The 11 fixed-point attractors recovered by the GHRN1 model are shown. Ten of the eleven recovered attractors match the expected activity configurations, which is not the case for the central pro-vascular TD3 attractor; for this attractor we indicated in blue the activities of the nodes that disagree with their expected activity.

https://doi.org/10.1371/journal.pcbi.1005488.g003

ARF10 in the pro-vascular attractors, suggests that additional constraints concerning the regulation of these ARFs need to be taken into account. ARF10 expression is low in the QC and the ground tissues, while it is relatively higher in the developing xylem and the columella (Fig 4; S1 Fig). On the other hand, ARF5 is expressed in the columella, the QC and the developing xylem, and its expression is lower in the ground tissues (Fig 4; S1 Fig). The expression patterns of ARF5 and ARF10 in the RAM tissues could be underlying a differential regulation of *WOX5* expression by auxin in different regions of the RAM. In fact, the expression analyses that showed opposite *WOX5* responses to auxin were done using different portions of the root, enriched with different tissues of the RAM [31,58]. Since not much is known about the transcriptional regulation of these ARFs, we searched in the literature and found that JKD directly binds to the promoter of ARF10 but the effect of this interaction is unknown [45]. We also noticed that JKD and ARF10 have complementary expression patterns (Fig 4). Given this evidence and because SHR and JKD can form dimers to regulate gene expression [28,62], we first hypothesized that the SHR-JKD dimer represses ARF10 expression. Then, we noticed that

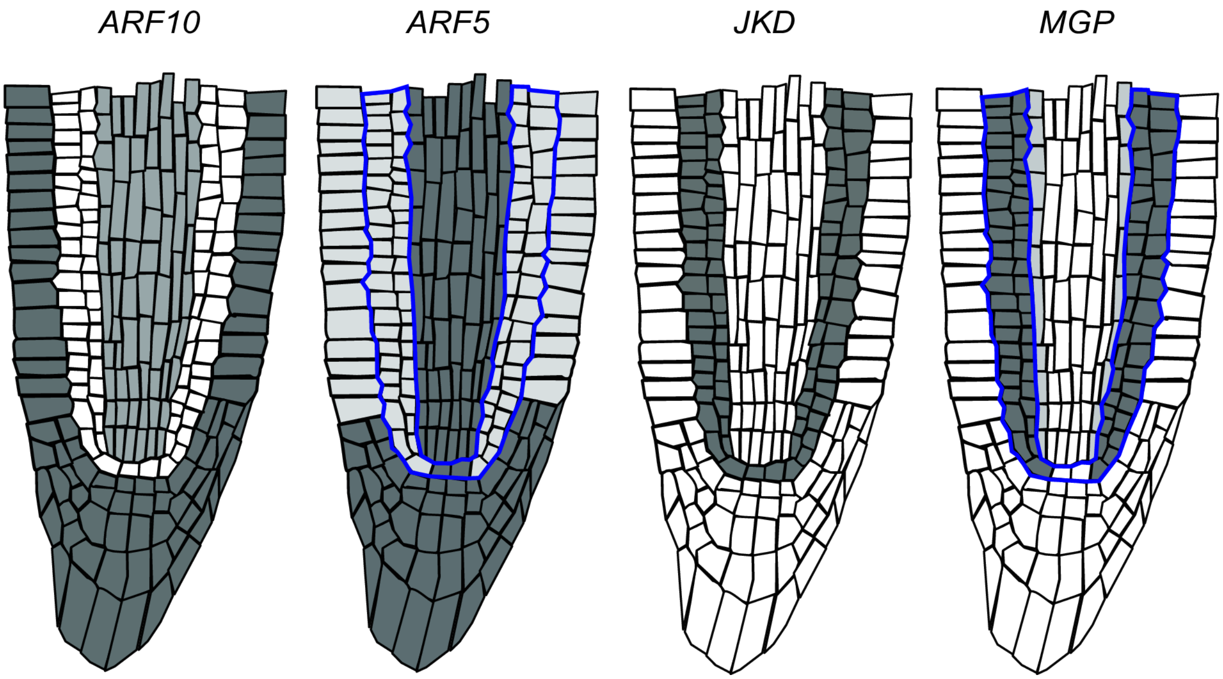

Fig 4. Activity of the ARFs that regulate *WOX5* in the RAM of *A. thaliana*. Inferred expression pattern of *ARF10* [87,89] and *ARF5* [83,87,89] in the RAM. The expression pattern of *JKD* [63] and *MGP* [36] is also shown. Notice the complementary expression patterns of *ARF10* and *JKD* in the RAM, and *ARF5* and *MGP* in the adjacent layer to the stele (delineated in blue).

https://doi.org/10.1371/journal.pcbi.1005488.g004

MGP has a complementary expression pattern to that of ARF5 in the adjacent layer to the provascular tissues (Fig 4). Since SHR can also dimerize with MGP [28,62], as a second hypothesis we proposed that this dimer negatively regulates ARF5 expression. These hypotheses imply that JKD and MGP might provide target specificity to the previously reported repression of ARF10 and ARF5 by SHR [43]. As mentioned previously, the current understanding of WOX5 expression is that two parallel pathways regulate it: the GRAS transcription factors and the auxin pathway. However, it is important to notice that if different protein complexes formed between SHR and other transcription factors regulate the components of the auxin signaling pathway that are involved in WOX5 regulation, as we are proposing here, it will imply that these pathways are not parallel. We integrated these hypotheses and the rest of the regulatory interactions (Table 1) in the GHRN1 model (Fig 2B). The logical rules and Boolean functions of the GHRN1 model can be found in S3 Appendix. We included the two hypotheses in the GHRN1 model because an exhaustive analysis of the dynamic possibilities [91,92] showed that by including separately the hypotheses it was not possible to recover attractors that correspond to the expected configurations of the cells at the RAM. On the contrary, the GHRN1 model recovered 11 identical fixed-point attractors (Fig 3) independently of the updating regime employed (Methods), indicating that these attractors are robust and emerge as a consequence of the topology and of the regulatory interactions integrated in the model, and not of the updating regime used. We will refer to these 11 fixed-point attractors as the original attractors of the model. We recovered 6 additional cyclic attractors when we solved with the synchronous updating regime; these cyclic attractors result from the coexistence of MIR166 and PHB, and of MGP and WOX5 (S2 Appendix). The fact that the cyclic attractors appear only under the synchronous regime suggests that they are an artifact of this updating scheme, as was later confirmed with the continuous version of the model.

The activity configurations of the 11 fixed-point attractors correspond to the expected ones of the cell types of the RAM that we aimed to describe (Fig 3), including the QC attractor and the correct activity profiles of ARF5 and ARF10 (Fig 4). The attractors that correspond to the endodermis, peripheral pro-vascular and central pro-vascular tissues were recovered by duplicate with the nodes Auxin and ARF either active or inactive (Fig 3B). These attractors correspond to cells of the PD (hereafter referred as the PD attractors) when they were active, or TD (TD attractors) when they were inactive. The activity of ARF10 in the PD attractors agrees with what was expected; it is active in the central and peripheral pro-vascular PD attractors and inactive in the endodermis PD and the QC attractors (Figs 3 and 4). On the other hand, ARF5 is active in the QC, the peripheral and central pro-vascular PD attractors, but not in the Endodermis PD attractor, as expected (Figs 3 and 4). These ARFs have different roles in the regulation of cell behavior at the RAM, particularly in the regulation of WOX5. As they are not expressed homogenously, this suggests that the regulatory interactions that underlie their expression patterns regulate how cells will respond to auxin. In the GHRN1 model the proposed regulators of ARF5 and ARF10 are key regulators of cell fate, indicating that the regulation of WOX5 by auxin might be tissue-specific. This is an interesting result because it constitutes a system-level mechanism implying that the regulatory network formed by hormones and transcriptional regulators might link the acquisition of cell identity with the differential capacity to respond to auxin in the RAM.

The TD attractors are characterized by the activity of the node AUXIAA indicating the inactivity of the auxin signaling pathway. The model recovered two central pro-vascular TD attractors (central pro-vascular TD1 and central pro-vascular TD2) that match the expected configurations (Fig 3B). Of the attractors representing the TD of the RAM, the CK signaling pathway was found active only in these central pro-vascular TD1/2 attractors, agreeing with this tissue being the main site of CK signaling in the TD of the RAM [4,26]. The difference between these two central pro-vascular TD1/2 attractors is that the central pro-vascular TD2 attractor shows activity of CLE40 and no activity of SHR. Regarding the validity of this attractor, a translational reporter has shown that the signaling peptide CLE40 is present in the pro-vascular tissues of the TD [75], and *in silico* visualization of root tip expression patterns indicates that *SHR* expression is dramatically decreased near the TD [42,85]. We recovered an additional central pro-vascular TD3 attractor that is similar to the central pro-vascular TD2 attractor but has activity of the nodes of both the CK and the Auxin pathways (indicated with blue in Fig 3B). This activity configuration does not correspond to the activity configuration that has been described for this zone as it is expected to have only CK signaling activity. It is possible that the unexpected activity configuration of the central pro-vascular TD3 attractor could be representing a transitioning state of the central pro-vascular tissues between the PD and the TD of the RAM. In this sense, the activity of CLE40 and the decrease in SHR activity could be important signals preceding the end of cell proliferation in the RAM. Lastly, two attractors can be identified as root cap tissues. These attractors differ in the activity of the nodes Auxin, ARF, ARF5 and ARF10 (Fig 3). As auxin signaling is known to regulate the differentiation of the root cap through ARF10 [31], we interpreted these attractors as cell types of the columella initial cells (Root Cap 1) and differentiated root cap cells (Root Cap 2). Importantly, the model recovers the co-activity of the auxin and CK signaling pathways in the Root Cap 2 attractor, as indeed it is observed in the root cap of *A*. *thaliana* [52–54].

Next we tested what happens to the 11 original attractors if we only consider direct but not the indirect regulation in our model in some particular cases (Fig 2). That is, what happens if we do not consider JKD as a positive regulator of SCR (as it does not regulate directly SCR, but promotes the nuclear localization of its activator, SHR) and inversely, what happens if we do not consider SCR as a positive regulator of JKD (for the same reason). We removed

individually each of these indirect interactions and evaluated the impact of such removals in the original attractors. We found that in the first case, the model with a modified logical rule for SCR still recovered the 11 original attractors, plus a new attractor to which we could not attribute any biological meaning as no cell in the RAM has been identified with such activity configuration (it had activity of CK, AUXIAA, SCR, SHR, PHB and MGP). Hence, the removed restriction is crucial to restrain the dynamics of the network to converge only to the expected attractors, but not strictly necessary to recover the 11 original attractors. In the second simulation we simplified the logical rule of JKD such that it is only regulated by SHR and PHB, and found that the model recovers 11 attractors, 9 of them with a perfect correspondence to the 11 original attractors. The two attractors that do not match the expected activity configurations were those corresponding to the peripheral pro-vascular tissues of the PD and the TD. In such cases JKD is active, which does not match what is experimentally observed; this gene expression is restricted to the ground tissues and the QC [63]. Moreover, in the peripheral pro-vasculature PD attractor ARF10 is not active while WOX5 is; this also contrasts with what has been documented for these tissues. Therefore, in this second simulation the constraint imposed by SCR is necessary to recover the correct activity pattern of JKD in the model. Finally, if we remove both interactions at the same time, we recover a cumulative effect of leaving out the two restrictions: we recovered 9 of the original attractors, two incorrect peripheral pro-vascular attractors, and an attractor with not known biological meaning. In summary, these three simulations show that the two regulatory interactions tested are fundamental for the correct *in silico* description of cell fate acquisition in the context of the GHRN1 model. Also, it further validates the formalism used in the GHRN1 Boolean network to integrate diverse experimental information, and this model itself, to describe the overall structure of interactions among the components considered. It also shows that the restrictions considered in the logical functions grounded on experimental data, are fundamental to understand the documented patterns of expression.

In summary, this analysis shows that the minimal GHRN1 model that includes two novel hypotheses recovers the gene expression and hormone activity configurations described for different cell types of the RAM and the root cap of *A. thaliana*. It also strongly suggests that the regulatory effect of auxin over WOX5 activity depends on the ARFs that are present in each cell type of the RAM, and predicts attractors that might correspond to uncharacterized cell types that according to experimental data are found in the central pro-vascular tissues of the TD of the RAM.

The recovered configurations of the GHRN1 model are robust

We performed two analyses to test the robustness of the GHRN1 model [93]. The first test estimates the frequency of recovering the original attractors in perturbed copies of the GHRN1 model; and the second test evaluates if the emergence of the attractors is independent of the formalism used to model the activity of the nodes (discrete or continuous).

Robustness of the discrete GHRN1 model to perturbations of the Boolean functions. We quantified the frequency of recovery of the original attractors in 100 randomly perturbed copies of the GHRN1 model. The perturbed copies of the GHRN1 were generated with point, but permanent, random modifications of the truth tables (see Methods for details). We compared the results of perturbing the GHRN1 against that of 1,000 topologically equivalent random networks (i.e., random networks with the same number of nodes and input regulators per node as the GHRN1), equally perturbed. The random networks showed a skewed distribution towards recovering a low percentage of their original attractors, with an average of 18.88% (Fig 5A). On the contrary, the GHRN1 model recovered >50% of the original attractors on average,

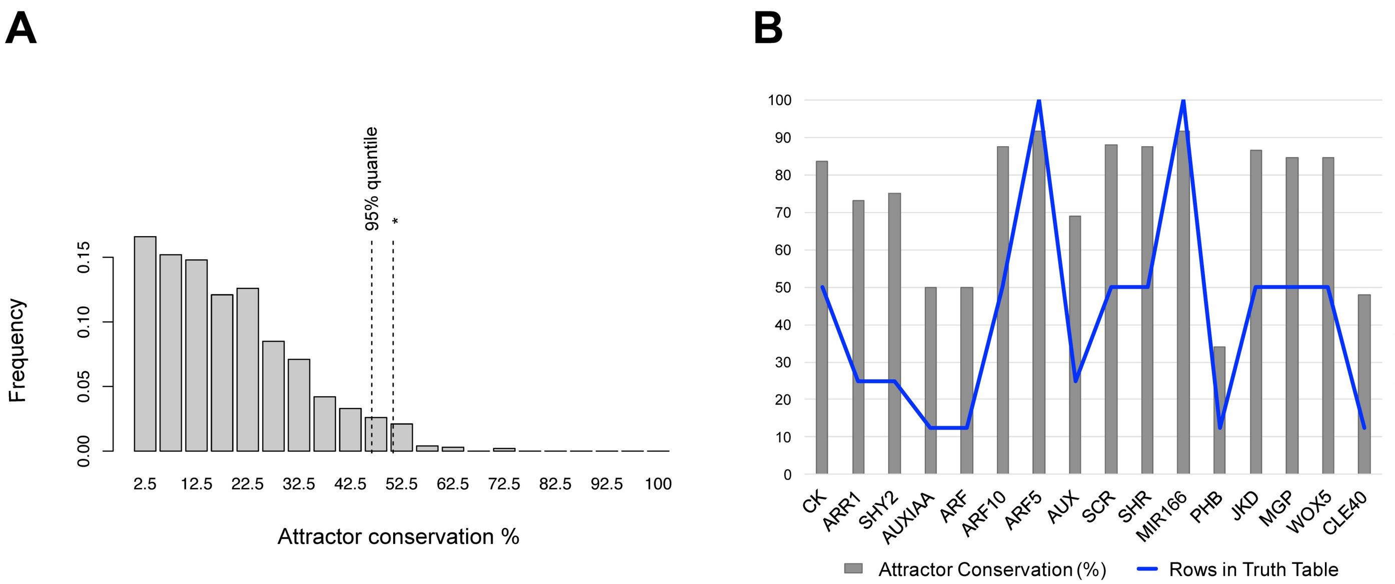

Fig 5. Robustness of the GHRN1 Boolean model to perturbations. A) The histogram shows the frequency of attractor recovery for a population of 1,000 random networks perturbed 100 times. The dashed lines show the 95% quantile and (*) the frequency of recovery for the GHRN1 model with the same number of perturbations. B) For each node it is shown the percentage of conservation of the original attractors in the systematic perturbation of the Boolean function of each node (gray bars), and the number of rows in its truth table (blue line).

https://doi.org/10.1371/journal.pcbi.1005488.g005

which is more than 97% of the random networks. Thus, as expected, this experimentally grounded network is more robust than the random networks. Consequently, this property is not related to the number of nodes and their connectivity in the GHRN1 network but to the specific interactions among the nodes, that were grounded on experimental data. Nevertheless, the robustness of the GHRN1 model is not as high as that reported for other biological networks [94–97]. This could be due to missing components and regulatory interactions.

To get additional insight concerning the robustness of the GHRN1 model, we systematically changed one by one every output value of the truth table of every node in the model (Methods) and found the attractors recovered by each perturbed network. Based on this analysis, we aimed at exploring if some of the original attractors were more prone to be lost as a way of suggesting a lack of regulatory interactions that stabilize them. We found that all of the attractors were lost and conserved to the same extent: all were lost in approximately 20% of the perturbations and conserved in the remaining 80% simulations (S2A Fig). Thus, this first analysis suggests that all the attractors have a similar propensity to be conserved or lost in the GHRN1 model under the simulated perturbations. Next, we analyzed the frequency at which new attractors appeared in the perturbations. We found that 92% of the 237 new attractors were recovered in only one perturbation (S2B Fig). Careful examination of these attractors showed that they constitute variations of the original 11 attractors with ectopic or absent expression of a node. The remaining 8% of the new attractors were recovered no more than three times, and also correspond to activity configurations not observed at the RAM. These results indicate that the new attractors are not easily reachable, but arise due to particular alterations of the Boolean functions.

Finally, using the results of the systematic perturbation analysis we identified the nodes that when perturbed were more prone to lose the original attractors. The perturbation of most nodes truth tables recovered in average more than 50% of the original attractors; this was not the case for AUXIAA, ARF, PHB and CLE40 (Fig 5B). Therefore, as expected from analyses of other networks, the network is more sensitive to alterations in certain nodes than in others.

We found that the higher sensitivity of these four nodes correlated with the size of their truth tables, as they are the only nodes in the GHRN1 network that have only one input regulator (Fig 5B). The impact of altering these nodes may be explained by the fact that they have very small truth tables and a single output alteration implies a relatively larger perturbation. Also, ARF and AUXIAA represent several redundant proteins that were not explicitly and individually considered. So the alteration in them, represents the alteration of a complete set of partially redundant components. This lack of detail about the redundancy in the components of the auxin signaling pathway could be related with the low frequency of attractor recovery for these nodes. Other possibility is that the four identified nodes which perturbations cause larger alterations in the original attractors, indeed hold key positions in the GHRN1. In summary, we were able to identify ARF, AUXIAA, PHB and CLE40 as the most sensitive nodes in the GHRN1 model, which in the case of the auxin signaling components could be related to their redundancy. It is possible that as more evidence is known about how the expression and activity of these four nodes are regulated in the RAM, including this information in the GHRN1 model will potentially increase their robustness in this perturbation analysis.

Robustness of the attractors to changes in the interaction kinetic functions. To get a system of ordinary differential equations (ODE) of the GHRN1 model, we approximated the Boolean step function of each node to a continuous one (Methods). Because we did not estimate the parameters for all the nodes of the GHRN1 model, as an alternative we analyzed 100 sets of parameters to test their effect in the steady states reached by the continuous model. In each set, the parameters controlling the production and degradation of each node were selected at random from predefined ranges (Methods). These 100 different systems of ODEs were solved from 10,000 random initial conditions all of which converged to the same 11 attractors described by the Boolean network (S5 Appendix). This analysis indicates that the 11 fixed-point attractors recovered by the Boolean model are still steady states in the continuous system, suggesting that they emerge independently of the kinetic parameters. Because we did not recover the 6 cyclic attractors found in the synchronic updating scheme of the Boolean model, we were interested in knowing their fate in the continuous system. Because we knew that the system was rather robust to the parameters, we used the same production and degradation parameters for all the nodes and set the initial conditions to each of the activity configurations of the 17 attractors recovered by the Boolean model (S2 Appendix). As expected, we found that the 11 fixed-point attractors were maintained as steady states in the continuous version of the GHRN1 model (S5 Appendix). On the contrary, the cyclic attractors reached one of the following configurations: central pro-vascular TD1, central pro-vascular TD2, central pro-vascular TD3, peripheral pro-vascular TD, central pro-vascular PD, endodermis PD and QC. This analysis supports that cyclic attractors are artifacts of the synchronous updating scheme in the Boolean version of the model. Lastly, we used 100,000 random initial conditions to test if we could find new steady states in the continuous regime. We found that all the initial conditions tested converged to one of the 11 activity configurations (S5 Appendix).

Altogether, these results indicate that the behavior of the GHRN1 network is robust to the modeling framework and to the specific parameters used, given that the 11 fixed-point attractors of the model are steady states under a discrete and a continuous formalism. Also, we did not find any additional steady states in our exploration of the continuous system.

Experimental validation of GHRN1 model: Simulation of gain and loss of function mutants

As a means to validate the GHRN1 model, we simulated the constitutive activation (GOF) and inactivation or loss of function (LOF) of every node of the model. The attractors recovered by

each mutated network were compared with the reported root mutant phenotypes, when data was available. The rest constitute novel predictions in the context of the restrictions considered in the model shown here (see Model Assumptions). The recovered configurations in the mutant simulations were the same for the Boolean and the continuous version of the model (Fig 6), as could have been predicted from the robustness analyses and the exploration of the continuous version of the GHRN1 model. The attractors recovered in the GOF/LOF simulation can be found in S6 Appendix (Methods). To make the comparison between the simulation results and experimental data, we gathered information from the literature about the expression patterns of the components of the GHRN1 in the corresponding mutant backgrounds. With this information we assessed if the attractors corresponded with what was observed experimentally given the components that we included in the model. A summary of the comparison between the *in silico* and the root phenotypes can be found in S7 Appendix. Below, we mentioned some of the simulations with particularly interesting results.

Some mutant simulations lost attractors corresponding to cell types that conform the radial pattern of the RAM as has indeed been observed in several reports. For example, the GOF of MIR166 and PHB [30,64], and the LOF of JKD [28,36], SCR [37], SHR [38], MIR166 [64] and PHB [30,64] are among these cases that result in the loss of the endodermis, peripheral or central pro-vascular tissues (Fig 6 and S7 Appendix). Defects in the specification of the QC cells have been described in some mutants, and the QC attractor was lost in the corresponding simulations. These simulations include the GOF of CLE40 [75,76] and ARF10 [31], and the LOF of ARF5 [27], SCR [27], SHR [27], JKD [28] and WOX5 [27] (Fig 6 and S7 Appendix). It is noteworthy to highlight that the simulations of the LOF of SCR and SHR do not have a QC attractor, consistent with their previous notion as necessary regulators for WOX5 expression in the QC [27]. Even though SHR and SCR do not regulate WOX5 directly in the GHRN1 model, they do regulate directly/indirectly the activity of ARF10 and in the absence of any of them, ARF10 will be active in all the PD attractors. Therefore, these simulations indicate that even though SCR and SHR may not regulate WOX5 directly, they are necessary for its activity as experimentally reported in the RAM [27]. Furthermore, the model predicts that the lack of WOX5 expression in these mutants might be accompanied by a broader expression domain of ARF10 in the RAM.

Three simulations are particularly interesting because the predicted negative regulatory interaction between SHR-JKD and ARF10 provides an explanation to their results. The first simulation is the GOF of PHB that does not have an attractor corresponding to the QC (Fig 6A and S7 Appendix). It has been demonstrated that PHB represses WOX5 expression during embryonic development [98] and this simulation suggests that this could also be happening post-embryonically. Additionally, the model suggests that the repressive action of PHB over WOX5 is constrained by JKD. In the simulation, a GOF of PHB represses the activity of JKD activity [64], which leads to an ectopic activation of ARF10 in all the PD attractors, akin to be active in the entire PD of the RAM (S6 Appendix). Under these conditions WOX5 cannot be active due to the presence of ARF10 in all the PD attractors. Similarly, there is no QC attractor in the simulation of the LOF of MIR166 (Fig 6B) that is a repressor of PHB. Experimentally, the importance of MIR166 in the QC has not been conclusive [30], and our model shows that it might be an important constraint to maintain PHB out of these cells as a necessary condition for WOX5 activity. Another interesting simulation is the LOF of JKD that has no QC attractor (Fig 6B) and in which ARF10 is expressed in all the PD attractors (S6 Appendix). The *jkd* mutants have a misspecification of the QC cells [28], which has been previously explained due to the decrease in SCR expression. Nevertheless, the *jkd scr* double mutants have a more severe phenotype than the *scr* single mutant [28], suggesting that the role of *jkd* in the QC goes beyond the regulation of *SCR* expression. The simulation of the LOF of JKD indicates that in

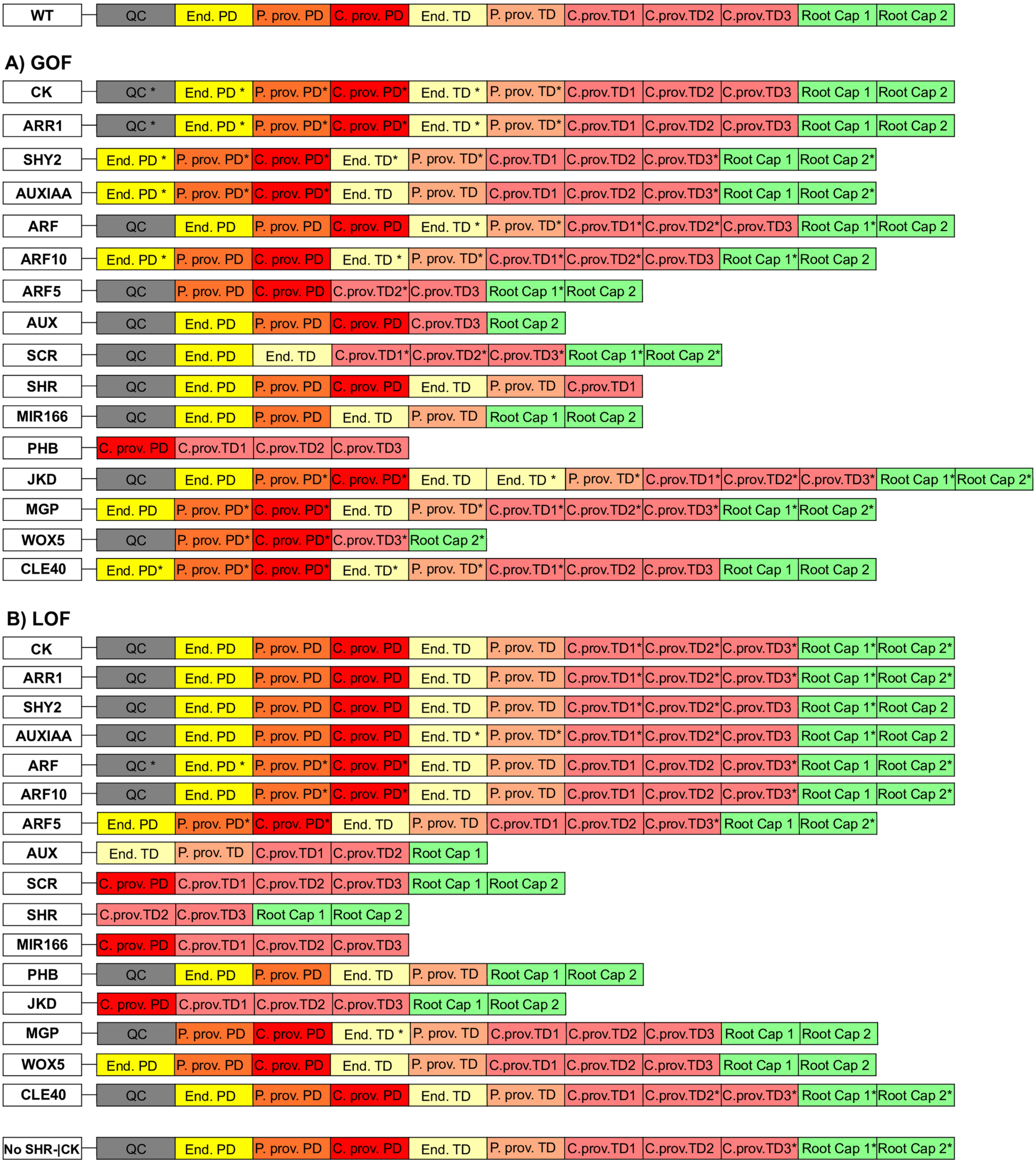

Fig 6. Attractors recovered in the GOF and LOF simulations. The results of the GOF (A) and LOF (B) simulations of all nodes are shown. The attractors that are not identical to the 11 original attractors are indicated with * (see Methods). The color code is as in Fig 1.

https://doi.org/10.1371/journal.pcbi.1005488.g006

addition to JKD being necessary for SCR expression in the QC, it might also be necessary to repress ARF10. Otherwise, WOX5 cannot be active because of the ectopic activity of ARF10, as explained above.

The role of the GRAS transcription factors and auxin pathways had been described as independent of each other at the RAM, but the GHRN1 model presented here shows that there are multiple regulatory interactions among them (Fig 2). Interestingly, in the simulation of the GOF of SHR the activity of the nodes representing the components of the auxin signaling pathway recovered the expected configurations of the PD and TD attractors (S6 Appendix). Conversely, in the GOF simulation of Auxin or ARF the activity profiles of the GRAS transcription factors are unaffected (S6 Appendix). These simulations show that, as it has been observed experimentally, these two pathways appear to be parallel even though multiple regulatory interactions exist among them in the GHRN1.

Some simulations could not be compared directly with experimental data because the chemical fields that underlie the RAM organization cannot be included in the Boolean formalism used here. For example, several mutants have quantitative alterations in the size of the RAM domains (S7 Appendix). As the Boolean network model put forward here is a unicellular model, the comparison between the corresponding simulations and the experimental phenotypes is not completely feasible unless assuming that the loss of an attractor is equal to a partial decrease in the size of that domain. But in principle the simulation results are unicellular and discrete (1 or 0) and in these cases are not comparable with such experimental phenotypes. This is the case for the LOF/GOF simulations of auxin (Fig 6). Another example is the simulation of the LOF of ARF10 (Fig 6B) that predicts that WOX5 will be active in the central and peripheral pro-vascular PD attractors (S6 Appendix). Experimentally, this is not observed as the expression of WOX5 is still confined to its regular position in the *arf10 arf16* double mutant [99]. The expression domain of the QC-specific marker QC25 is slightly expanded in this genetic background [100], but not as much as the simulation suggests. An aspect that is not considered by the Boolean network is the spatial distribution of auxin in the RAM, which is unperturbed in the *arf10 arf16* double mutant [100]. The role of the auxin chemical field or uncharacterized redundancy in the auxin signaling pathway involved in repressing WOX5 could be the reason this phenotype is not observed *in vivo*. Therefore, it is not possible to make a quantitative comparison between some of the simulations and the experimental evidence. But, it remains a possibility to observe the expression of WOX5 in the pro-vascular tissues of the RAM by altering the auxin distribution in the *arf10 arf16* double mutant.

The GHRN1 model that we proposed is a useful framework that can be used to explore the specific role of particular regulatory interactions of a node under study. Thereby, we simulated a mutant where SHR does not inhibit CK, to evaluate the effect of this particular regulatory interaction in the model. In this simulation, the GHRN1 model still recovered 11 steady-state attractors (Fig 6B). The only difference with the original attractors is that the root cap attractors do not have activity of the CK signaling pathway in this simulation (S6 Appendix), showing that the repression of CK biosynthesis by SHR is necessary to explain the co-activity of auxin and CK signaling in the root cap. As the PD and TD attractors were recovered with the expected activity configurations (Fig 6B), this result is consistent with the repression of CK by SHR not being involved in the regulation of the transition from proliferation to differentiation in the RAM [79]. Therefore, the cross-talk of hormones and transcription factors that we integrated in the GHRN1 model provides a possible explanation not only for the paradoxical effects of auxin responses in the RAM, but also for the emergence of the co-activity of auxin and CK pathways in the root cap.

Overall the analysis of mutants showed that the GHRN1 model agrees in most cases with what has been reported experimentally (S7 Appendix), including the simulation of the LOF

mutants of SCR, SHR, JKD and the GOF of PHB that have a misspecification of the QC cells (Fig 6). Moreover, the model elucidated a potential new role of MIR166 in the maintenance of the QC cells, proving that the core system-level module that we uncovered is a valuable theoretical framework that can be used to predict and discern on the regulatory role of a component in the context of the rest of the interactions integrated in the model. Our analysis suggests that WOX5 is not active in any of these simulations because of the broader activity domain of ARF10. These results provide further support to the hypothesis concerning ARF10 regulation proposed here, and show that the GHRN1 model contains the components and interactions necessary and sufficient to recover attractors that correspond to the genetic and auxin/CK activity profiles that have been described for different cell types in the RAM for WT and mutants of *A*. *thaliana*. Moreover, the simulation where SHR does not repress CK is another example where the GHRN1 model proved to be a useful framework to evaluate the role of particular interactions in the regulation of the RAM.

Discussion

In this study we proposed a dynamic regulatory network model that is sufficient to describe how the cross-talk among the auxin/CK signaling pathways and some transcriptional regulators that have been shown to be important in root development, underlies the emergence of these hormones and factors configurations as observed in different types of cells along the *A*. *thaliana* RAM. The uncovered regulatory module provides the first system-level mechanistic explanation for the emergence of coupled transcriptional and hormonal activity configurations along the root longitudinal axis. Importantly, our analyses indicate that the effect of auxins over WOX5 activity might depend on the cellular context that, at the same time is established by the multi-stability of the regulatory network. These results argue that the complex qualitative regulatory network formed by hormones and transcriptional regulators is important for the process of cell-fate specification, as well as to understand how cells "interpret" auxin signals, and how the different profiles of hormonal (auxins and CK) activity emerge along the longitudinal axis of the *A*. *thaliana* RAM.

The fixed-point attractors recovered by the model correspond to the genetic configurations of the transcription factors considered in the minimal GHRN1, that are associated to different cell types of the RAM, in addition to the auxin/CK activities that have been also shown to correlate with the cell behaviors observed in the PD and TD of the RAM and the root cap (Fig 3). Contrary to the cyclic attractors recovered in the synchronous updating scheme in the discrete model, the 11 fixed-point attractors were found to be also attractors when we solved the model with the asynchronous updating schemes and in the continuous version of the GHRN1 model. Therefore, the 11 attractors seem to emerge from the restrictions imposed by the interactions integrated in the model presented here, rather than by an artifact due to the updating scheme. The GHRN1 model predicted the existence of two attractors that according to expression data may correspond to uncharacterized cell types that can be found near the central pro-vascular tissues of the TD at the RAM [42,75,85]. These attractors have activity of CLE40, PHB and the CK pathway, and no activity of SHR (Fig 3B). The recovery of these attractors by the GHRN1 model raises the question of the functional importance of these cell types in the RAM. Since at least one of the CLE40 receptors is expressed in the epidermis from the TD onwards [90], it is possible that this signaling peptide is involved in cell communication from the main source of CK in the central cylinder to the outer tissues of the TD at the RAM. As in the TD, cells of different types stop proliferating at different times [17,18], it is tempting to speculate that this signaling could be involved in cell-cycle/growth synchronization among tissue layers prior to rapid cell growth at the elongation zone. Additionally, the decrease of SHR expression could

cause an increase in CK levels in the TD associated to the transition from proliferation to differentiation. Further experiments are needed to explore both hypotheses.

The GHRN1 model includes previously reported interactions but also new hypotheses regarding the regulation of ARF10 and ARF5 by key regulators of cell fate (Fig 2B and 2C). These hypotheses are based on information from the literature. Once introduced in the model, the uncovered module has the necessary and sufficient set of components and interactions for the minimal GHRN1 model to recover attractors with the activity profiles of these ARFs as observed in the cell types of the RAM (Fig 3A); including the QC attractor where ARF5 is active and ARF10 is not. The emergence of ARF10 and ARF5 activity configurations is a significant result of our model because these ARFs had been reported to regulate WOX5 expression in opposite directions, but we were lacking an explanation for this paradoxical behavior at the RAM. Our model suggests that these particular auxin responses might be context dependent. The fact that different RAM tissues differ in the activity of the ARFs, suggests that there is an inherent correspondence between the cell differentiation process and auxin responsiveness at the RAM of *A*. *thaliana*. Recently, it was reported that ARF10 could be a regulator of SHR as it physically binds its promoter [47], making it possible that ARF10 feedbacks on its regulators by quantitatively modulating the expression of SHR in the pro-vascular tissues, where both are co-expressed. Moreover, PHB promotes ARF5 expression at the RAM [101], suggesting that the regulatory interactions among transcriptional regulators and the components of the hormonal signaling pathways that we explore in this study are a recurrent theme in RAM regulation.

It is important to highlight that the regulation of the RAM depends on many more hormones and genetic regulators than the ones we integrated in the GHRN1 model. In this sense, the GHRN1 model presented here is not a complete one. Many more components are at play during the post-embryonic development of the RAM of *A*. *thaliana*. Nonetheless, the model constitutes a starting dynamic regulatory module into which additional components can be included in future modeling efforts. Such components could be additional transcriptional regulators [43–47,56], hormone signaling/transport/metabolism pathway components [20–22] and cell cycle regulators that are main regulators of cellular patterning at the RAM [3,32,40, 97,102]. High-throughput approaches have postulated putative regulatory interactions of key regulators of the RAM [43–47], as well as described the activity patterns of many genes with cell type resolution [22,42,46,47,85]. Such computational efforts have been incredibly valuable to know how genes are spatially expressed in the RAM under different conditions. In some of these analyses it has been shown that the cellular context is instructive to specify transcriptional responses [22,46], but the underlying mechanisms setting out the context are not entirely understood. The GHRN1 we propose in this paper comprise a robust and well-validated module that offers an opportunity to integrate the role of additional transcription factors as they are functionally characterized. But the model presented here already comprises a first dynamic mechanism to understand the emergence of cell context from the concerted activity of multiple regulators and auxin/CK signaling. Particularly, it will be interesting to study the links between the mechanisms underlying cell differentiation and the specificity in the responses of transcription factors/hormones that clearly have context-specific and important roles in RAM regulation [22,46]. Such is the case of the auxin-responsive *PLETHORA* genes that have been shown to have spatial specific responses. How such responses are established is not entirely understood [46]. Another extension of the model could imply improving the details about how the expression patterns of SHR, JKD and SCR are regulated as several candidate genes for such regulation have been found recently [47]. Understanding how SCR and JKD can be expressed independently of SHR in the context of the GHRN1 model could be useful to explore the dynamic effects of the dual role of JKD over SCR [45]. This surely will also

help connect the GHRN1 core regulatory module with many other pathways or modules. In this way, the resolution of the mechanisms underlying cell-fate specification during RAM development could be improved. The GHRN1 module uncovered here, thus constitutes a building block to further generate systemic and mechanistic understanding of the role of these genes in the context of the regulatory interactions included so far in the GHRN1.

We tested the robustness of the GHRN1 model to random perturbations and compared it with that of topologically equivalent random networks. We found that the attractors of the GHRN1 model are more robust than those recovered from random networks, but not as robust as those of other network models grounded on biological information [94–97]. This is likely to be because some important components and interactions are still missing in the GHRN1 model. For instance, we did not consider the redundancy of the hormone signaling pathways, which is likely to provide additional robustness to the system. In fact, the attractors of the network were more prone to be altered if the AUXIAA and ARF logical rules were altered (Fig 5B). As more information becomes available we will be able to break down the individual role of each ARF and Aux/IAA protein, and directly address the role of their redundancy and specific responses in RAM development.

The models were validated by simulating the GOF/LOF mutants of each component. In most cases the simulated and observed altered configurations or phenotypes agreed (Fig 6 and S7 Appendix). For example, the misspecification of the QC cells in the *scr*, *shr* and *jkd* mutants [24,27,28] was recovered as a loss of the QC attractor when these loss of function mutants were simulated (Fig 6 and S7 Appendix). The results show that these regulators are necessary for WOX5 activity, although they are not direct regulators of it in the GHRN1 model. Instead, they form part of a complex regulatory network and are indirectly involved in the regulation of the QC cell fate. The simulation of LOF of MIR166 also lost the QC attractor. Experimentally, the role of MIR166 in the QC cells had not been conclusive [30], but in the context of our model it becomes clear that it is a necessary restriction to repress PHB to indirectly maintain the QC activity configuration. This simulation is particularly interesting because it is a clear example of how the type of theoretical framework that we put forward in this paper can be used to clarify the role of particular regulators in the context of the collective activity of the other network components during RAM development. In this case, the model suggests that MIR166 at the QC might have a key and previously unknown role in maintaining this cell type. These simulations that do not recover the QC attractor (LOF SCR, SHR, JKD and MIR166), show that additionally to not having WOX5 activity, these mutants might have activity of ARF10 in all the PD cells. The latter could be tested by studying the activity of the ARF10 transcriptional reporter [87] in the corresponding mutant backgrounds. We would expect ARF10 to be expressed all over the meristem. The current understanding of *WOX5* transcriptional regulation states that it is promoted by the joint activity of two parallel pathways: the GRAS transcription factors SCR and SHR, that regulate the radial patterning of the root, and auxin that is involved in root apical-basal patterning [24,27]. Our model is consistent with this idea as SCR, SHR and auxin are indeed positive, but indirect regulators of WOX5 activity. Furthermore, our study complements this idea by showing that the components of these presumably parallel pathways converge in ARF10/ARF5 to regulate WOX5 expression. Then, the effect of these regulators over WOX5 and in the diverse cell behaviors in the RAM needs to be interpreted in the context of the proposed regulatory module formed by transcriptional regulators and the auxin/CK signaling pathways that we integrated in the minimal GHRN1 model.

By using a Boolean approach, we showed that it is possible to explain the opposite effects of auxin on WOX5 expression without assuming dosage-dependent effects. Still, the discrete nature of this approach imposes some limitations when comparing the results of the GOF/LOF simulations with experimental data. A Boolean network renders the possible activity

profiles of a regulatory system, but it does not allow quantitative analyses or the study of how the spatio-temporal patterns of different cell types emerge. Certainly, such behaviors cannot be modeled or predicted with a Boolean model, but complex systems in which many non-linear interactions are involved have dynamics that are mainly driven by the structure of the interactions, rather than the details of the kinetic functions [103]. Such systems are robust to quantitative alterations of the kinetic functions. Hence, important system-level information can be obtained from such qualitative models. Indeed, despite the limitations of using a discrete modeling approach, for most simulated mutants we were able to make a comparison with experimental phenotypes (S7 Appendix). The reasons some simulations could not be validated was either due to the lack of experimental information, due to the redundancy of the components of the hormone signaling pathways, or due to the fact that the comparisons were not feasible. An example of this last case is the LOF simulation of auxin that results in no PD attractors (Fig 6B). As the chemical fields in the RAM are rarely severely perturbed due to the redundancy in the mechanisms underlying hormone metabolism and transport, the results of these simulations cannot be directly compared with experimental data, unless assuming that no PD/TD attractors imply smaller domains in the RAM. Another example is the simulation of the LOF of ARF10 that predicts that WOX5 may be active in the pro-vascular tissues of the PD of the RAM, which does not happen in the *arf10 arf16* genetic background as the auxin gradient is not severely perturbed [99,100]. Thus, even though the GHRN1 model has a significant predictive potential to describe the genetic expression and hormonal activity configurations of cells in the RAM for WT and most mutants, the model is still incomplete. There are additional regulatory constraints that need to be taken into account to have a more complete understanding of the system.

For example, the mechanisms underlying the formation and maintenance of the chemical fields and those responsible for the spatial organization of different cell types of the RAM constitute additional and important developmental constraints. These mechanisms are well described [3,7,36,48,51,86], and a future challenge will imply understanding the activity of the GHRN1 network in a multi-level model that considers the role of cell-cell interactions, physical-chemical fields defined explicitly in spatio-temporal terms, or the intercellular movement of some of the components of the proposed module, to explain the emergence of the positional information for the organization and temporal appearance of the different cell types along the RAM. In this sense it will be of particular interest to study the quantitative and temporal effects of auxin in the organization of the RAM [41] and the regulation of the formative divisions of the ground tissue [3]. The contribution put forward here is a fundamental integrative step to further pursue such spatio-temporal modeling and understanding of RAM patterning.

Generic transcriptional-hormone feedback mechanisms underlie a potentially generic information processing mechanisms in multicellular organisms

Signaling molecules regulate different aspects of multicellular development. Understanding how cells acquire positional information or interpret a signaling molecule is key for understanding how ordered patterns of development, growth and regeneration emerge in multicellular organisms. In this paper we explored with a dynamic model the interactions among transcriptional regulators and two hormones. This model enabled us to explain, among other things, the paradoxical effects of auxin over a key gene for RAM organization: *WOX5*. Our model indicates that auxin readout might depend on which signaling components are present in a cell, which is a result of the differentiation process (Fig 7). The gene regulatory networks are inherently multi-stable, each state corresponding to a configuration characteristic of a cell

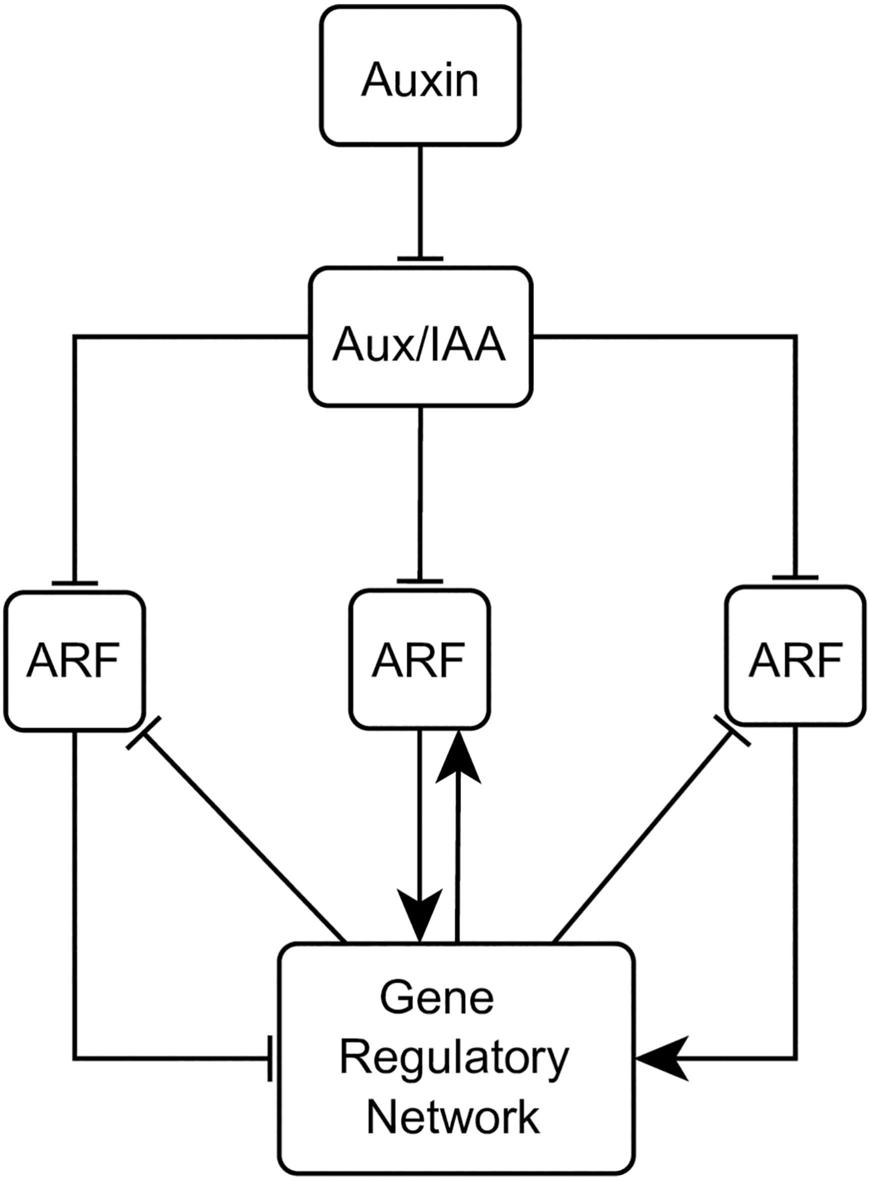

Fig 7. Nonlinear coupling between the auxin signaling pathway and the gene regulatory network. The model suggests that the readout of auxin could be mediated by complex interactions between its signaling pathway and the gene regulatory network.

https://doi.org/10.1371/journal.pcbi.1005488.g007

type, and this feature is translated into the multiplicity of auxin responses. As long as there is redundancy and functional divergence in the components of a signaling pathway, it is possible that the correspondence between the readout of signaling molecules and cell differentiation that we proposed for the RAM of *A*. *thaliana* is a common feature in multicellular development. Such mechanism implies that as cells acquire a cell fate with a particular hormone/regulators configuration, they may also establish a differential capacity to respond to generic signaling molecules. The RAM constitutes a SCN with a generic cellular pattern mostly shared by niches of all multicellular organisms [9,10]. The model provided here bears novel hypotheses that can be tested experimentally and it thus contributes a useful theoretical framework to continue integrating additional genetic and chemical components and interactions that

underlie cell differentiation and patterning in a SCN. Therefore, the GHRN1 model we proposed in this paper is a significant step forward toward understanding the complex interactions among genetic and hormone signaling pathways in multicellular development and patterning of stem cell niches.

Methods

Boolean networks

Boolean networks allow the qualitative study of the dynamics of cell fate acquisition considering the concerted action of several main regulators [104–108]. In Boolean networks the nodes are modeled as discrete variables that have one of two possible activity states: 0 (inactive) and 1 (active). The system is solved in discrete time steps, such that the state of a node will be influenced by the state of its regulators in a previous time step. Each node has a truth table (also known as Boolean function [105–108]) that explicitly states all the activity combinations of the state of its regulators (input), and the state of the node in the next time step (output). These truth tables/Boolean functions can be summarized into equivalent logical functions using the operators AND, OR and NOT and take the general form:

$$x_i(t+1) = F_i(x_1(t), \ldots, x_k(t)) \tag{1}$$

Where x_i $(t + 1)$ is the state of a node x_i at time $t + 1$ and x_1 (t),. . ., x_k (t) are the states of its regulators at time t. For example, for a node x with regulators y and z and with the logical rule $x(t+1) = y(t)$ *AND* $z(t)$, its derived truth table/Boolean function is shown in Table 2.

The system can be solved synchronously if the state of all nodes is updated each time step, or asynchronously if the nodes have different updating schemes. The system can be explored exhaustively from all possible initial conditions (2^number of nodes) by applying the logical functions iteratively until eventually the system reaches an activity configuration that is recovered periodically. Such configurations are known as attractors (steady states) and are interpreted as the phenotypes or cell types of the system under study [104]. Attractors can be fixed-point or cyclic depending if they reached one state or if they oscillate between two or more states. We solved the GHRN models using three updating schemes: synchronous, all nodes are updated each time step; random asynchronous, where at each time step a single node is randomly selected to be updated; and weighed asynchronous, where nodes were classified in two groups of fast or slow and updated accordingly. For the weighed asynchronous updating scheme, we classified the nodes as fast or slow updating nodes based on their molecular nature. In particular, we defined the signaling proteins and some elements known to move between cells as fast updating nodes, whereas transcriptional regulators were considered as slow updating nodes.

Table 2. Truth table for the logical rule *x(t+1) = y(t) AND z(t)*.

y(t)	*z(t)*	*x(t+1)*
0	0	0
0	1	0
1	0	0
1	1	1

https://doi.org/10.1371/journal.pcbi.1005488.t002

For the GOF and LOF mutant simulations we fixed the state of the mutated node to 1 or 0, respectively. This is equivalent to substituting all the output positions to 1/0 in the Boolean function of that node. For this analysis we used the random asynchronous updating scheme. For the WT simulations, to link an activity configuration to an expected attractor we expected a 100% similarity between the expected and recovered attractors (considering that * means the one could be either 1 or 0); in the case of the mutant analysis, an attractor was associated to an expected cell type as long as the activity of 12 nodes was the same between the expected and recovered attractors (0.75% similarity), but making sure that the transcription factors known to underlie cell fate had the correct activity patterns. The analysis of the Boolean networks was performed with the R package, BoolNet [109].

We used Griffin for the exhaustive exploration of the alternative GHRNs models to prove that no model can be recovered without including the two hypotheses we proposed. Griffin is a software that transforms a set of constrains (in our case the set of expected attractors and the topology of the network) into a Boolean sentence. Using symbolic algorithms, Griffin finds assignments of the Boolean variables that make the sentence true. Each assignment contains a set of Boolean functions that recovers the expected attractors. Griffin software was published before, and more information of how it works can be found in [91]. An updated version of Griffin is available upon request.

Robustness analysis of the Boolean networks

The robustness of the attractors of the Boolean networks was estimated by calculating the frequency of recovering the original attractors in perturbed copies of the network. The perturbed networks were constructed by changing an output value of the truth table/Boolean function from 0 to 1 (bitflip), or vice versa. Notice that the effect of fixing the state of a node to 0 or 1, as in the simulation of GOF and LOF mutants, is different from a bitflip in a position of the truth table/Boolean function. In the first instance, the activity of a node is fixed during throughout the simulation, whereas in the second case the logical function of the node is altered.

In the first robustness analysis, we compared the robustness of the GHRN1 against random networks. The perturbations were performed at random 100 times for the GHRN1 network and for 1,000 random networks with the same topological features (same number of nodes with the same number of input regulators). We solved each network from all possible initial conditions, and quantified if the original attractors were recovered in these perturbed networks. A significance threshold was set at 0.05, meaning that the attractor conservation in the GHRN1 network is higher than >95% of the random networks. In the second robustness analysis, we repeated this analysis by systematically altering each position of all truth table/Boolean functions of the GHRN1 model.

Continuous model

For the continuous model we approximated the Boolean functions to continuous sigmoidal functions following the protocol reported in [110]. The activity of each node in the continuous model is described by a differential equation of the form:

$$\frac{dx_i}{dt} = \frac{-e^{0.5h} + e^{-hw_i}}{(1 - e^{0.5h})(1 + e^{-h(w_i - 0.5)})} - \gamma_i x_i \qquad (2)$$

In this equation the first and second terms describe the production and degradation of the node, respectively. This equation adjusts the dynamic of each node to a sigmoidal function. The parameter h determines the strength of the interactions and controls if the activation curve of a node resembles a step function, a logistic function or a straight line; γ is the degradation rate

and *w* is the continuous form of the logical functions using fuzzy logic. The *w*'s used in this analysis can be found in S4 Appendix. We analyzed 100 sets of parameters for each ODEs system. In each set the value of the parameters of each node were selected at random from the ranges (10–50) and (0.5–1) for h and γ, respectively. Each model was solved from 10,000 random initial conditions to recover the steady states at which they converged. The sets of parameters used for this analysis can be found in S5 Appendix.

For the simulation of mutants, we fixed the value of *w* to 1 (GOF) or 0 (LOF) for the perturbed node. The parameters used to analyze the fate of the attractors of the Boolean network in the continuous version and for the mutant analysis were the same for all nodes: $h = 50$, and $\gamma = 1$. To analyze the steady states at which the system converges, we considered a node as active (1) when it had a value of >0.9; if its state was <0.1 then the node was inactive (0).

Expression maps of ARF5, ARF10 and ARF16

The expression maps of *ARF5*, *ARF10* and *ARF16* in the RAM (S1B Fig) integrate information from transcriptional data of specific root tissues from [89], transcriptional reporters from [87], and in a few instances translational reporters from [83,100].

Model assumptions

Boolean logic can qualitatively formalize many sorts of regulatory interactions and dependencies in the form of logical functions, to dynamically study their concerted action. The nodes of the Boolean network models presented here represent hormones, signaling proteins or genes. For hormones, if a node is active (1) it means that its cellular concentration is high enough to activate its signaling pathway. Otherwise, the node is inactive (0). For genes, inactivity means that the gene is not expressed, or could be expressed but not active due to post-translational regulation. Thus, the activity of nodes that represent genes means that they are expressed and active at the protein level. Below, we provide two representative examples of how experimental information at different levels of regulation was formalized into Boolean functions. The first example is SCR. Functionally, SCR expression requires SHR and SCR itself, that form a protein complex that act as transcriptional activators [38,43,61]. Moreover, JKD and SCR form protein complexes with SHR and promote its nuclear localization [36,61], otherwise it will be located in the cytoplasm. Then, JKD is a necessary constraint for SCR expression (even though JKD does not regulate SCR expression directly) given its regulatory role on SHR cellular localization. In the model, cellular compartments are not considered explicitly. Thus, we formalized these documented interactions and dependencies among the nodes in an abstract but valid way, as *SCR(t+1) = SCR(t) and SHR(t) and JKD(t)*. Hence, we can study the overall structure of the documented regulatory interactions beyond biochemical details. Below, we show the truth table derived from the logical function of SCR (Table 3), to show how the output value of each row was decided based on experimental information.

Another example is ARR1. It has been reported that SCR represses the expression of ARR1 in the QC and the RAM [34,57]. Moreover, ARR1 at the protein level has to be activated by the phosphorylation cascade initiated by CK. Therefore, the logical rule of ARR1 is *ARR1(t+1) = not SCR(t) and CK(t)*. This logical rule considers that one condition for ARR1 to be active is that SCR has to be absent. Additionally, it is necessary to have activity of CK. The truth table of ARR1 is shown below (Table 4).

These two examples (SCR and ARR1) show how the activity of a node depends on various regulatory processes that may act at different levels of molecular regulation, and how this regulation can be integrated in the form of a logical function. This means that for a node to be

Table 3. Truth table of SCR with the rationale behind each output value.

SCR (t)	SHR (t)	JKD (t)	SCR (t+1)	Rationale
0	0	0	0	SCR is expressed in the shootward part of the meristem of *shr* mutants [38], indicating that it can be expressed independently of SHR. There are several candidate regulators of SCR [47] which remain to be validated and functionally characterized in order to include them in the model. Thus, given the regulators considered, SCR = 0 when all its regulators are inactive the previous time step.
0	0	1	0	The amplification of JKD expression in *shr* mutants showed that JKD represses SCR expression in the absence of SHR [45]. Then, when only JKD is active, the next time step SCR will be inactive.
0	1	0	0	For SHR to be able to activate SCR it needs to be located in the nucleus, which is regulated by SCR and JKD [36,61].
0	1	1	0	When JKD and SHR are active, the next time step SCR will be inactive. This is because even though JKD promotes SHR nuclear localization at a certain extent, the maximum effect over SCR expression is observed when SCR is also present [61]. It is important to remember that our model does not describe quantitative variations in activity; instead when a node is active it means maximum levels of activity. Importantly, if we relax this criterion and allow SCR activity in this context, the model still recovers 11 attractors.
1	0	0	0	SCR cannot self-activate its promoter by itself.
1	0	1	0	In the absence of SHR, JKD does not allow SCR activity the next time step. Again, corresponding to a repressive action of JKD over SCR [45].
1	1	0	0	It has been shown that SCR and SHR are not sufficient to effectively activate SCR promoter in protoplasts [61]. We followed this evidence given that in the Boolean model we aim at understanding how maximal levels of activity are attained (instead of quantitative variations). In the RAM, this requirement for JKD is evident in the *jkd* mutant that shows a lack of SCR activity in the QC [28]. If we relax this criterion, we still recover the 11 original attractors plus a new attractor with no biological meaning.
1	1	1	1	In the model SCR activity requires the activity of SCR, SHR and JKD the previous time step. This is supported by the requirement of these three proteins to activate effectively SCR promoter [61], and for full SHR nuclear retention [61].

https://doi.org/10.1371/journal.pcbi.1005488.t003

active, several conditions likely to be acting in different regulatory processes need to be satisfied.

In the model we consider only regulatory interactions that modify the activity of a node, and not regulatory interactions that modulate the magnitude of activity of a node. For example, we did not consider the activation of Aux/IAA expression by the ARFs because it does not compromise auxin responses [111]. Moreover, we did not consider the repression of PHB by CK, because it has been proposed not to compromise its activity but to modulate it quantitatively [4]. The logical functions of the nodes AUXIAA and ARF only consider post-translational regulation because the transcriptional regulators that control their expression are unknown for most of them. Thus, we assumed that these nodes have a basal transcription rate. MIR166 moves from its site of synthesis in the adjacent layer to the pro-vascular tissues, where it promotes the degradation of PHB. A mutual degradation between MIR166 and PHB has been suggested to form sharp boundaries of activity [50]. We followed this assumption such that the non-cell autonomous role of microRNA MIR166 was modeled considering that is active either in its site of synthesis (where SHR and SCR are active) or if PHB is not present. The same assumption regarding movement was made for SHR; it is active either in its site of synthesis (SHR) or where JKD and SCR are present as SHR move between cells and these

Table 4. Truth table of ARR1.

SCR(t)	*CK(t)*	*ARR1(t+1)*
0	0	0
0	1	1
1	0	0
1	1	0

https://doi.org/10.1371/journal.pcbi.1005488.t004

proteins promote its nuclear retention outside its site of synthesis. For the second instances to be valid, at least one attractor must represent its site of synthesis.

ARF10 and ARF16 expression patterns are different at the RAM (S1A Fig) suggesting that they are differentially regulated there. However, their expression patterns overlap and they redundantly promote the differentiation of the root cap [89,100]. Therefore, we considered them as a single node. For the validation analysis, the ARF10 GOF simulation was compared with a resistant line that overexpresses ARF16 (Pro_{35S}:mARF16), and in the LOF analysis of ARF10 the comparison was made with the double mutant *arf10 arf16* (S7 Appendix).

Supporting information

S1 Fig. ARF5 and ARF10 expression in various RAM tissues. Expression of *ARF5* and *ARF10* in the columella, QC, developing xylem, endodermis and cortex cells (data taken from [89]). Bars represent the standard error.
(TIF)

S2 Fig. Attractor robustness and new attractors recovered in the systematic perturbation of the Boolean functions of the GHRN1 model. (A) The frequency at which each attractor was recovered or lost in the systematic perturbation of all Boolean functions is shown. (B) The graph shows the percentage of the simulations at which the new attractors are recovered.
(TIF)

S1 Appendix. Logical rules of the GHRN model.
(TXT)

S2 Appendix. Attractors recovered by the GHRN models. The wild-type attractors recovered by the GHRN and GHRN1 models are shown. The steady state attractors were recovered when solved with three different updating schemes (Methods). The cyclic attractors are recovered only in the synchronic update.
(XLSX)

S3 Appendix. Logical rules and Boolean functions of the GHRN1 model.
(TXT)

S4 Appendix. Continuous version of the logical functions of the GHRN1 model. The *w*'s function of each node of the GHRN1 model is shown.
(TXT)

S5 Appendix. Analysis of the continuous version of the GHRN1 model. The 11 attractors of the discrete GHRN1 model are maintained as steady states in the continuous extensions of the models. Moreover, the cyclic attractors and 100,000 random initial conditions converged only to one of these 11 steady states. The sets of random parameters used for the parameter analysis are also shown.
(PDF)

S6 Appendix. Attractors recovered in the simulation of GOF and LOF mutants in the Boolean and the continuous version of the GHRN1 model. The attractors recovered in the *in silico* simulations of GOF and LOF mutants for the GHRN1 model are shown. The nodes that change activity in comparison with the wild-type simulations are indicated in red.
(XLSX)

S7 Appendix. Comparison between the *in silico* phenotypes and experimental evidence. Tables 1 and 2 show the comparison for the GOF and LOF simulations, respectively.
(PDF)

Acknowledgments

This work is presented in partial fulfillment towards Mónica L García Goméz' doctoral degree in the "Programa de Doctorado en Ciencias Biomédicas de la Universidad Nacional Autónoma de México (UNAM)". This work greatly benefited from the input of Prof. Joseph G. Dubrovsky. We acknowledge the "Academic Writing" team of the "Centro de Estudios de Posgrado, UNAM" for their help with the drafting of this manuscript. We acknowledge Elizabeth Ortíz-Gutiérrez for sharing programming codes, Yamel Sonia Ugartechea Chirino for sharing root images, and the help from Diana Romo with various logistical tasks.

Author Contributions

Conceptualization: MLGG ERAB.

Investigation: MLGG.

Methodology: MLGG EA ERAB.

Resources: MLGG EA.

Supervision: ERAB.

Writing – original draft: MLGG EA ERAB.

References

1. Azpeitia E, Benítez M, Vega I, Villarreal C, Alvarez-Buylla ER. Single-cell and coupled GRN models of cell patterning in the Arabidopsis thaliana root stem cell niche. BMC Syst Biol. 2010 Oct 5; 4:134. https://doi.org/10.1186/1752-0509-4-134 PMID: 20920363
2. Azpeitia E, Weinstein N, Benítez M, Mendoza L, Alvarez-Buylla ER. Finding missing interactions of the Arabidopsis thaliana root stem cell niche gene regulatory network. Front Plant Sci. 2013 Apr 30; 4:110. eCollection 2013. https://doi.org/10.3389/fpls.2013.00110 PMID: 23658556
3. Cruz-Ramírez A, Díaz-Triviño S, Blilou I, Grieneisen VA, Sozzani R, Zamioudis C, et al. A bistable circuit involving SCARECROW-RETINOBLASTOMA integrates cues to inform asymmetric stem cell division. Cell. 2012 Aug 31; 150(5):1002–15. Epub 2012 Aug 23. https://doi.org/10.1016/j.cell.2012.07.017 PMID: 22921914
4. Ioio RD, Galinha C, Fletcher AG, Grigg SP, Molnar A, Willemsen V, et al. A PHABULOSA/cytokinin feedback loop controls root growth in Arabidopsis. Curr Biol. 2012 Sep 25; 22(18):1699–704. Epub 2012 Aug 16. https://doi.org/10.1016/j.cub.2012.07.005 PMID: 22902752
5. Barrio RA, Romero-Arias JR, Noguez MA, Azpeitia E, Ortiz-Gutiérrez E, Hernández-Hernández V, et al. Cell patterns emerge from coupled chemical and physical fields with cell proliferation dynamics: the Arabidopsis thaliana root as a study system. PLoS Comput Biol. 2013 May 2; 9(5):e1003026. Epub 2013 May 2. https://doi.org/10.1371/journal.pcbi.1003026 PMID: 23658505
6. Dyson RJ, Vizcay-Barrena G, Band LR, Fernandes AN, French AP, Fozard JA, et al. Mechanical modelling quantifies the functional importance of outer tissue layers during root elongation and bending. New Phytol. 2014 Jun 1; 202(4):1212–22. Epub 2014 Mar 18. https://doi.org/10.1111/nph.12764 PMID: 24641449
7. Band LR, Wells DM, Fozard JA, Ghetiu T, French AP, Pound MP, et al. Systems analysis of auxin transport in the Arabidopsis root apex. The Plant Cell. 2014 Mar; 26(3):862–75. Epub 2014 Mar 14. https://doi.org/10.1105/tpc.113.119495 PMID: 24632533
8. Álvarez-Buylla ER, Dávila-Velderrain J, Martínez-García JC. Systems Biology Approaches to Development beyond Bioinformatics: Nonlinear Mechanistic Models Using Plant Systems. BioScience. 2016 Apr 6:biw027.
9. Sablowski R. Plant and animal stem cells: conceptually similar, molecularly distinct?. Trends Cell Biology. 2004 Nov 30; 14(11):605–11.
10. Scheres B. Stem-cell niches: nursery rhymes across kingdoms. Nat Rev Mol Cell Biol. 2007 May 1; 8 (5):345–54. https://doi.org/10.1038/nrm2164 PMID: 17450175
11. Dolan L, Janmaat K, Willemsen V, Linstead P, Poethig S, Roberts K, et al. Cellular organisation of the Arabidopsis thaliana root. Development. 1993 Sep 1; 119(1):71–84. PMID: 8275865

12. Barlow PW. The concept of the stem cell in the context of plant growth and development. Stem cells and tissue homeostasis. 1978:87–113.
13. Barlow PW. Stem cells and founder zones in plants, particularly their roots. Stem cells. 1997:29–57.
14. Kidner C, Sundaresan V, Roberts K, Dolan L. Clonal analysis of the Arabidopsis root confirms that position, not lineage, determines cell fate. Planta. 2000 Jul 1; 211(2):191–9. https://doi.org/10.1007/s004250000284 PMID: 10945213
15. Cruz-Ramírez A, Díaz-Triviño S, Wachsman G, Du Y, Arteága-Vázquez M, Zhang H, et al. A SCARECROW-RETINOBLASTOMA protein network controls protective quiescence in the Arabidopsis root stem cell organizer. PLoS Biol. 2013 Nov 26; 11(11):e1001724. Epub 2013 Nov 26. https://doi.org/10.1371/journal.pbio.1001724 PMID: 24302889
16. Campilho A, Garcia B, Wijk HV, Campilho A, Scheres B. Time-lapse analysis of stem-cell divisions in the Arabidopsis thaliana root meristem. Plant J. 2006 Nov 1; 48(4):619–27. https://doi.org/10.1111/j.1365-313X.2006.02892.x PMID: 17087761
17. Baluška F, Kubica Š, Hauskrecht M. Postmitotic 'isodiametric'cell growth in the maize root apex. Planta. 1990 Jun 1; 181(3):269–74. https://doi.org/10.1007/BF00195876 PMID: 24196802
18. Ivanov VB, Dubrovsky JG. Longitudinal zonation pattern in plant roots: conflicts and solutions. Trends Plant Sci. 2013 May 31; 18(5):237–43. Epub 2012 Nov 2. https://doi.org/10.1016/j.tplants.2012.10.002 PMID: 23123304
19. Hayashi K, Hasegawa J, Matsunaga S. The boundary of the meristematic and elongation zones in roots: endoreduplication precedes rapid cell expansion. Sci Rep. 2013 Oct 14; 3:2723. https://doi.org/10.1038/srep02723 PMID: 24121463
20. Garay-Arroyo A, De La Paz Sánchez M, García-Ponce B, Azpeitia E, Álvarez-Buylla ER. Hormone symphony during root growth and development. Dev Dyn. 2012 Dec 1; 241(12):1867–85. Epub 2012 Oct 25. https://doi.org/10.1002/dvdy.23878 PMID: 23027524
21. Ubeda-Tomás S, Beemster GT, Bennett MJ. Hormonal regulation of root growth: integrating local activities into global behaviour. Trends in plant science. 2012 Jun 30; 17(6):326–31. https://doi.org/10.1016/j.tplants.2012.02.002 PMID: 22401844
22. Iyer-Pascuzzi AS, Jackson T, Cui H, Petricka JJ, Busch W, Tsukagoshi H, Benfey PN. Cell identity regulators link development and stress responses in the Arabidopsis root. Developmental cell. 2011 Oct 18; 21(4):770–82. https://doi.org/10.1016/j.devcel.2011.09.009 PMID: 22014526
23. Sabatini S, Beis D, Wolkenfelt H, Murfett J, Guilfoyle T, Malamy J, et al. An auxin-dependent distal organizer of pattern and polarity in the Arabidopsis root. Cell. 1999 Nov 24; 99(5):463–72. PMID: 10589675
24. Sabatini S, Heidstra R, Wildwater M, Scheres B. SCARECROW is involved in positioning the stem cell niche in the Arabidopsis root meristem. Genes Dev. 2003 Feb 1; 17(3):354–8. https://doi.org/10.1101/gad.252503 PMID: 12569126
25. Aida M, Beis D, Heidstra R, Willemsen V, Blilou I, Galinha C, et al. The PLETHORA genes mediate patterning of the Arabidopsis root stem cell niche. Cell. 2004 Oct 1; 119(1):109–20. https://doi.org/10.1016/j.cell.2004.09.018 PMID: 15454085
26. Ioio RD, Linhares FS, Scacchi E, Casamitjana-Martinez E, Heidstra R, Costantino P, et al. Cytokinins determine Arabidopsis root-meristem size by controlling cell differentiation. Curr Biol. 2007 Apr 17; 17(8):678–82. Epub 2007 Mar 15. https://doi.org/10.1016/j.cub.2007.02.047 PMID: 17363254
27. Sarkar AK, Luijten M, Miyashima S, Lenhard M, Hashimoto T, Nakajima K, et al. Conserved factors regulate signalling in Arabidopsis thaliana shoot and root stem cell organizers. Nature. 2007 Apr 12; 446(7137):811–4. https://doi.org/10.1038/nature05703 PMID: 17429400
28. Welch D, Hassan H, Blilou I, Immink R, Heidstra R, Scheres B. Arabidopsis JACKDAW and MAGPIE zinc finger proteins delimit asymmetric cell division and stabilize tissue boundaries by restricting SHORT-ROOT action. Genes Dev. 2007 Sep 1; 21(17):2196–204. https://doi.org/10.1101/gad.440307 PMID: 17785527
29. Ioio RD, Nakamura K, Moubayidin L, Perilli S, Taniguchi M, Morita MT, et al. A genetic framework for the control of cell division and differentiation in the root meristem. Science. 2008 Nov 28; 322(5906):1380–4. https://doi.org/10.1126/science.1164147 PMID: 19039136
30. Carlsbecker A, Lee JY, Roberts CJ, Dettmer J, Lehesranta S, Zhou J, et al. Cell signalling by microRNA165/6 directs gene dose-dependent root cell fate. Nature. 2010 May 20; 465(7296):316–21. Epub 2010 Apr 21. https://doi.org/10.1038/nature08977 PMID: 20410882
31. Ding Z, Friml J. Auxin regulates distal stem cell differentiation in Arabidopsis roots. Proc Natl Acad Sci U S A. 2010 Jun 29; 107(26):12046–51. Epub 2010 Jun 11. https://doi.org/10.1073/pnas.1000672107 PMID: 20543136

32. Sozzani R, Cui H, Moreno-Risueno MA, Busch W, Van Norman JM, Vernoux T, Brady SM, Dewitte W, Murray JA, Benfey PN. Spatiotemporal regulation of cell-cycle genes by SHORTROOT links patterning and growth. Nature. 2010 Jul 1; 466(7302):128–32. https://doi.org/10.1038/nature09143 PMID: 20596025

33. Garay-Arroyo A, Ortiz-Moreno E, de la Paz Sánchez M, Murphy AS, García-Ponce B, Marsch-Martínez N, de Folter S, Corvera-Poiré A, Jaimes-Miranda F, Pacheco-Escobedo MA, Dubrovsky JG. The MADS transcription factor XAL2/AGL14 modulates auxin transport during Arabidopsis root development by regulating PIN expression. The EMBO journal. 2013 Oct 30; 32(21):2884–95. https://doi.org/10.1038/emboj.2013.216 PMID: 24121311

34. Moubayidin L, Di Mambro R, Sozzani R, Pacifici E, Salvi E, Terpstra I, et al. Spatial coordination between stem cell activity and cell differentiation in the root meristem. Dev Cell. 2013 Aug 26; 26 (4):405–15. https://doi.org/10.1016/j.devcel.2013.06.025 PMID: 23987513

35. Takahashi N, Kajihara T, Okamura C, Kim Y, Katagiri Y, Okushima Y, et al. Cytokinins control endocycle onset by promoting the expression of an APC/C activator in Arabidopsis roots. Curr Biol. 2013 Sep 23; 23(18):1812–7. Epub 2013 Sep 12. https://doi.org/10.1016/j.cub.2013.07.051 PMID: 24035544

36. Long Y, Smet W, Cruz-Ramírez A, Castelijns B, de Jonge W, Mähönen AP, et al. Arabidopsis BIRD zinc finger proteins jointly stabilize tissue boundaries by confining the cell fate regulator SHORT-ROOT and contributing to fate specification. Plant Cell. 2015 Apr 1; 27(4):1185–99. Epub 2015 Mar 31. https://doi.org/10.1105/tpc.114.132407 PMID: 25829440

37. Di Laurenzio L, Wysocka-Diller J, Malamy JE, Pysh L, Helariutta Y, Freshour G, et al. The SCARECROW gene regulates an asymmetric cell division that is essential for generating the radial organization of the Arabidopsis root. Cell. 1996 Aug 9; 86(3):423–33. PMID: 8756724

38. Helariutta Y, Fukaki H, Wysocka-Diller J, Nakajima K, Jung J, Sena G, et al. The SHORT-ROOT gene controls radial patterning of the Arabidopsis root through radial signaling. Cell. 2000 May 26; 101 (5):555–67. PMID: 10850497

39. Nakajima K, Sena G, Nawy T, Benfey PN. Intercellular movement of the putative transcription factor SHR in root patterning. Nature. 2001 Sep 20; 413(6853):307–11. https://doi.org/10.1038/35095061 PMID: 11565032

40. Ishida T, Adachi S, Yoshimura M, Shimizu K, Umeda M, Sugimoto K. Auxin modulates the transition from the mitotic cycle to the endocycle in Arabidopsis. Development. 2010 Jan 1; 137(1):63–71. https://doi.org/10.1242/dev.035840 PMID: 20023161

41. Mähönen AP, ten Tusscher K, Siligato R, Smetana O, Díaz-Triviño S, Salojärvi J, et al. PLETHORA gradient formation mechanism separates auxin responses. Nature. 2014 Nov 6; 515(7525):125–9. Epub 2014 Aug 24. https://doi.org/10.1038/nature13663 PMID: 25156253

42. Brady SM, Orlando DA, Lee JY, Wang JY, Koch J, Dinneny JR, et al. A high-resolution root spatiotemporal map reveals dominant expression patterns. Science. 2007 Nov 2; 318(5851):801–6. https://doi.org/10.1126/science.1146265 PMID: 17975066

43. Levesque MP, Vernoux T, Busch W, Cui H, Wang JY, Blilou I, et al. Whole-genome analysis of the SHORT-ROOT developmental pathway in Arabidopsis. PLoS Biol. 2006 May 2; 4(5):e143. Epub 2006 May 2. https://doi.org/10.1371/journal.pbio.0040143 PMID: 16640459

44. Cui H, Hao Y, Kovtun M, Stolc V, Deng XW, Sakakibara H, et al. Genome-wide direct target analysis reveals a role for SHORT-ROOT in root vascular patterning through cytokinin homeostasis. Plant Physiol. 2011 Nov 1; 157(3):1221–31. Epub 2011 Sep 27. https://doi.org/10.1104/pp.111.183178 PMID: 21951467

45. Moreno-Risueno MA, Sozzani R, Yardımcı GG, Petricka JJ, Vernoux T, Blilou I, Alonso J, Winter CM, Ohler U, Scheres B, Benfey PN. Transcriptional control of tissue formation throughout root development. Science. 2015 Oct 23; 350(6259):426–30. https://doi.org/10.1126/science.aad1171 PMID: 26494755

46. Santuari L, Sanchez-Perez G, Luijten M, Rutjens B, Terpstra I, Berke L, Gorte M, Prasad K, Bao D, Timmermans-Hereijgers JL, Maeo K. The PLETHORA gene regulatory network guides growth and cell differentiation in Arabidopsis roots. The Plant Cell. 2016 Dec 5:tpc-00656.

47. Sparks EE, Drapek C, Gaudinier A, Li S, Ansariola M, Shen N, Hennacy JH, Zhang J, Turco G, Petricka JJ, Foret J. Establishment of Expression in the SHORTROOT-SCARECROW Transcriptional Cascade through Opposing Activities of Both Activators and Repressors. Developmental Cell. 2016 Oct 27.

48. Grieneisen VA, Xu J, Marée AF, Hogeweg P, Scheres B. Auxin transport is sufficient to generate a maximum and gradient guiding root growth. Nature. 2007 Oct 25; 449(7165):1008–13. https://doi.org/10.1038/nature06215 PMID: 17960234

49. Mironova VV, Omelyanchuk NA, Novoselova ES, Doroshkov AV, Kazantsev FV, Kochetov AV, et al. Combined in silico/in vivo analysis of mechanisms providing for root apical meristem self-organization and maintenance. Ann Bot. 2012 Jul 1; 110(2):349–60. Epub 2012 Apr 16. https://doi.org/10.1093/aob/mcs069 PMID: 22510326

50. Muraro D, Mellor N, Pound MP, Lucas M, Chopard J, Byrne HM, et al. Integration of hormonal signaling networks and mobile microRNAs is required for vascular patterning in Arabidopsis roots. Proc Natl Acad Sci U S A. 2014 Jan 14; 111(2):857–62. Epub 2013 Dec 31. https://doi.org/10.1073/pnas.1221766111 PMID: 24381155

51. Muraro D, Larrieu A, Lucas M, Chopard J, Byrne H, Godin C, et al. A multi-scale model of the interplay between cell signalling and hormone transport in specifying the root meristem of Arabidopsis thaliana. J Theor Biol. 2016 May 6. Epub 2016 May 6.

52. Brunoud G, Wells DM, Oliva M, Larrieu A, Mirabet V, Burrow AH, et al. A novel sensor to map auxin response and distribution at high spatio-temporal resolution. Nature. 2012 Feb 2; 482(7383):103–6. https://doi.org/10.1038/nature10791 PMID: 22246322

53. Zürcher E, Tavor-Deslex D, Lituiev D, Enkerli K, Tarr PT, Müller B. A robust and sensitive synthetic sensor to monitor the transcriptional output of the cytokinin signaling network in planta. Plant Physiol. 2013 Mar 1; 161(3):1066–75. Epub 2013 Jan 25. https://doi.org/10.1104/pp.112.211763 PMID: 23355633

54. Antoniadi I, Plačková L, Simonovik B, Doležal K, Turnbull C, Ljung K, et al. Cell-type-specific cytokinin distribution within the Arabidopsis primary root apex. Plant Cell. 2015 Jul 1; 27(7):1955–67. Epub 2015 Jul 7. https://doi.org/10.1105/tpc.15.00176 PMID: 26152699

55. Petersson SV, Johansson AI, Kowalczyk M, Makoveychuk A, Wang JY, Moritz T, et al. An auxin gradient and maximum in the Arabidopsis root apex shown by high-resolution cell-specific analysis of IAA distribution and synthesis. Plant Cell. 2009 Jun 1; 21(6):1659–68. Epub 2009 Jun 2. https://doi.org/10.1105/tpc.109.066480 PMID: 19491238

56. Cui H, Hao Y, Kong D. SCARECROW has a SHORT-ROOT-independent role in modulating the sugar response1. Plant physiology. 2012 Apr 1; 158(4):1769–78. https://doi.org/10.1104/pp.111.191502 PMID: 22312006

57. Moubayidin L, Salvi E, Giustini L, Terpstra I, Heidstra R, Costantino P, et al. A SCARECROW-based regulatory circuit controls Arabidopsis thaliana meristem size from the root endodermis. Planta. 2016 Feb 5:1–0. Epub 2016 Feb 5.

58. Gonzali S, Novi G, Loreti E, Paolicchi F, Poggi A, Alpi A, et al. A turanose-insensitive mutant suggests a role for WOX5 in auxin homeostasis in Arabidopsis thaliana. Plant J. 2005 Nov 1; 44(4):633–45. https://doi.org/10.1111/j.1365-313X.2005.02555.x PMID: 16262712

59. Tian H, Wabnik K, Niu T, Li H, Yu Q, Pollmann S, et al. WOX5–IAA17 feedback circuit-mediated cellular auxin response is crucial for the patterning of root stem cell niches in Arabidopsis. Mol Plant. 2014 Feb 28; 7(2):277–89. Epub 2013 Aug 12. https://doi.org/10.1093/mp/sst118 PMID: 23939433

60. Zhang W, Swarup R, Bennett M, Schaller GE, Kieber JJ. Cytokinin induces cell division in the quiescent center of the Arabidopsis root apical meristem. Curr Biol. 2013 Oct 21; 23(20):1979–89. Epub 2013 Oct 10. https://doi.org/10.1016/j.cub.2013.08.008 PMID: 24120642

61. Cui H, Levesque MP, Vernoux T, Jung JW, Paquette AJ, Gallagher KL, et al. An evolutionarily conserved mechanism delimiting SHR movement defines a single layer of endodermis in plants. Science. 2007 Apr 20; 316(5823):421–5. https://doi.org/10.1126/science.1139531 PMID: 17446396

62. Ogasawara H, Kaimi R, Colasanti J, Kozaki A. Activity of transcription factor JACKDAW is essential for SHR/SCR-dependent activation of SCARECROW and MAGPIE and is modulated by reciprocal interactions with MAGPIE, SCARECROW and SHORT ROOT. Plant Mol Biol. 2011 Nov 1; 77(4–5):489–99. Epub 2011 Sep 21. https://doi.org/10.1007/s11103-011-9826-5 PMID: 21935722

63. Hassan H, Scheres B, Blilou I. JACKDAW controls epidermal patterning in the Arabidopsis root meristem through a non-cell-autonomous mechanism. Development. 2010 May 1; 137(9):1523–9. Epub 2010 Mar 31. https://doi.org/10.1242/dev.048777 PMID: 20356954

64. Miyashima S, Koi S, Hashimoto T, Nakajima K. Non-cell-autonomous microRNA165 acts in a dose-dependent manner to regulate multiple differentiation status in the Arabidopsis root. Development. 2011 Jun 1; 138(11):2303–13. https://doi.org/10.1242/dev.060491 PMID: 21558378

65. Woodward AW, Bartel B. Auxin: regulation, action, and interaction. Ann Bot. 2005 Apr 1; 95(5):707–35. Epub 2005 Mar 4. https://doi.org/10.1093/aob/mci083 PMID: 15749753

66. Guilfoyle TJ, Hagen G. Auxin response factors. Curr Opin Plant Biol. 2007 Oct 31; 10(5):453–60. Epub 2007 Sep 27. https://doi.org/10.1016/j.pbi.2007.08.014 PMID: 17900969

67. Tiwari SB, Hagen G, Guilfoyle T. The roles of auxin response factor domains in auxin-responsive transcription. Plant Cell. 2003 Feb 1; 15(2):533–43. https://doi.org/10.1105/tpc.008417 PMID: 12566590

68. Vernoux T, Brunoud G, Farcot E, Morin V, Van den Daele H, Legrand J, et al. The auxin signalling network translates dynamic input into robust patterning at the shoot apex. Mol Syst Biol. 2011 Jan 1; 7(1).

69. Piya S, Shrestha SK, Binder B, Stewart CN, Hewezi T. Protein-protein interaction and gene co-expression maps of ARFs and Aux/IAAs in Arabidopsis. Front Plant Sci. 2014 Dec 23; 5:744. https://doi.org/10.3389/fpls.2014.00744 PMID: 25566309

70. Lee DJ, Park JW, Lee HW, Kim J. Genome-wide analysis of the auxin-responsive transcriptome downstream of iaa1 and its expression analysis reveal the diversity and complexity of auxin-regulated gene expression. J Exp Bot. 2009:erp230. Epub 2009 Aug 4.

71. Rademacher EH, Lokerse AS, Schlereth A, Llavata-Peris CI, Bayer M, Kientz M, et al. Different auxin response machineries control distinct cell fates in the early plant embryo. Dev Cell. 2012 Jan 17; 22 (1):211–22. https://doi.org/10.1016/j.devcel.2011.10.026 PMID: 22264733

72. Bishopp A, Lehesranta S, Vatén A, Help H, El-Showk S, Scheres B, Helariutta K, Mähönen AP, Sakakibara H, Helariutta Y. Phloem-transported cytokinin regulates polar auxin transport and maintains vascular pattern in the root meristem. Current Biology. 2011 Jun 7; 21(11):927–32. https://doi.org/10.1016/j.cub.2011.04.049 PMID: 21620705

73. Santner A, Calderon-Villalobos LI, Estelle M. Plant hormones are versatile chemical regulators of plant growth. Nat Chem Biol. 2009 May 1; 5(5):301–7. https://doi.org/10.1038/nchembio.165 PMID: 19377456

74. Moubayidin L, Perilli S, Ioio RD, Di Mambro R, Costantino P, Sabatini S. The rate of cell differentiation controls the Arabidopsis root meristem growth phase. Curr Biol. 2010 Jun 22; 20(12):1138–43. Epub 2010 Jun 3. https://doi.org/10.1016/j.cub.2010.05.035 PMID: 20605455

75. Stahl Y, Wink RH, Ingram GC, Simon R. A signaling module controlling the stem cell niche in Arabidopsis root meristems. Curr Biol. 2009 Jun 9; 19(11):909–14. Epub 2009 Apr 23. https://doi.org/10.1016/j.cub.2009.03.060 PMID: 19398337

76. Chu H, Liang W, Li J, Hong F, Wu Y, Wang L, et al. A CLE–WOX signalling module regulates root meristem maintenance and vascular tissue development in rice. J Exp Bot. 2013 Dec 1; 64(17):5359–69. Epub 2013 Sep 16. https://doi.org/10.1093/jxb/ert301 PMID: 24043854

77. Scacchi E, Salinas P, Gujas B, Santuari L, Krogan N, Ragni L, et al. Spatio-temporal sequence of cross-regulatory events in root meristem growth. Proc Natl Acad Sci U S A. 2010 Dec 28; 107 (52):22734–9. Epub 2010 Dec 13. https://doi.org/10.1073/pnas.1014716108 PMID: 21149702

78. Nordström A, Tarkowski P, Tarkowska D, Norbaek R, Åstot C, Dolezal K, et al. Auxin regulation of cytokinin biosynthesis in Arabidopsis thaliana: a factor of potential importance for auxin–cytokinin-regulated development. Proc Natl Acad Sci U S A. 2004 May 25; 101(21):8039–44. Epub 2004 May 14. https://doi.org/10.1073/pnas.0402504101 PMID: 15146070

79. Hao Y, Cui H. SHORT-ROOT regulates vascular patterning, but not apical meristematic activity in the Arabidopsis root through cytokinin homeostasis. Plant Signal Behav. 2012 Mar 1; 7(3):314–7. Epub 2012 Mar 1. https://doi.org/10.4161/psb.19118 PMID: 22476466

80. Sebastian J, Ryu KH, Zhou J, Tarkowská D, Tarkowski P, Cho YH, et al. PHABULOSA controls the quiescent center-independent root meristem activities in Arabidopsis thaliana. PLoS Genet. 2015 Mar 2; 11(3):e1004973. https://doi.org/10.1371/journal.pgen.1004973 PMID: 25730098

81. De Rybel B, Adibi M, Breda AS, Wendrich JR, Smit ME, Novák O, et al. Integration of growth and patterning during vascular tissue formation in Arabidopsis. Science. 2014 Aug 8; 345(6197):1255215. https://doi.org/10.1126/science.1255215 PMID: 25104393

82. Okushima Y, Overvoorde PJ, Arima K, Alonso JM, Chan A, Chang C, et al. Functional genomic analysis of the AUXIN RESPONSE FACTOR gene family members in Arabidopsis thaliana: unique and overlapping functions of ARF7 and ARF19. Plant Cell. 2005 Feb 1; 17(2):444–63. Epub 2005 Jan 19. https://doi.org/10.1105/tpc.104.028316 PMID: 15659631

83. Schlereth A, Möller B, Liu W, Kientz M, Flipse J, Rademacher EH, et al. MONOPTEROS controls embryonic root initiation by regulating a mobile transcription factor. Nature. 2010 Apr 8; 464 (7290):913–6. Epub 2010 Mar 10. https://doi.org/10.1038/nature08836 PMID: 20220754

84. Ulmasov T, Murfett J, Hagen G, Guilfoyle TJ. Aux/IAA proteins repress expression of reporter genes containing natural and highly active synthetic auxin response elements. Plant Cell. 1997 Nov 1; 9 (11):1963–71. https://doi.org/10.1105/tpc.9.11.1963 PMID: 9401121

85. Winter D, Vinegar B, Nahal H, Ammar R, Wilson GV, Provart NJ. An "Electronic Fluorescent Pictograph" browser for exploring and analyzing large-scale biological data sets. PloS one. 2007 Aug 8; 2 (8):e718. Arabidopsis eFP Browser. Available: http://bar.utoronto.ca/efp/cgi-bin/efpWeb.cgi?dataSource=Root. https://doi.org/10.1371/journal.pone.0000718 PMID: 17684564

86. Blilou I, Xu J, Wildwater M, Willemsen V, Paponov I, Friml J, et al. The PIN auxin efflux facilitator network controls growth and patterning in Arabidopsis roots. Nature. 2005 Jan 6; 433(7021):39–44. https://doi.org/10.1038/nature03184 PMID: 15635403

87. Rademacher EH, Möller B, Lokerse AS, Llavata-Peris CI, van den Berg W, Weijers D. A cellular expression map of the Arabidopsis AUXIN RESPONSE FACTOR gene family. Plant J. 2011 Nov 1; 68 (4):597–606. Epub 2011 Aug 30. https://doi.org/10.1111/j.1365-313X.2011.04710.x PMID: 21831209

88. Zhang H, Han W, De Smet I, Talboys P, Loya R, Hassan A, et al. ABA promotes quiescence of the quiescent centre and suppresses stem cell differentiation in the Arabidopsis primary root meristem. Plant J. 2010 Dec 1; 64(5):764–74. Epub 2010 Oct 26. https://doi.org/10.1111/j.1365-313X.2010.04367.x PMID: 21105924

89. Bargmann BO, Vanneste S, Krouk G, Nawy T, Efroni I, Shani E, et al. A map of cell type-specific auxin responses. Mol Syst Biol. 2013 Jan 1; 9(1):688.

90. Gifford ML, Dean S, Ingram GC. The Arabidopsis ACR4 gene plays a role in cell layer organisation during ovule integument and sepal margin development. Development. 2003 Sep 15; 130(18):4249–58. PMID: 12900442

91. Rosenblueth DA, Muñoz S, Carrillo M, Azpeitia E. Inference of Boolean networks from gene interaction graphs using a SAT solver. InInternational Conference on Algorithms for Computational Biology 2014 Jul 1 (pp. 235–246). Springer International Publishing.

92. Azpeitia E, Munoz S, Gonzalez-Tokman D, Martinez-Sanchez ME, Weinstein N, Naldi A, Alvarez-Buylla EE, Rosenblueth DA, Mendoza L. The combination of the functionalities of feedback circuits is determinant for the number and size of attractors of molecular networks. bioRxiv. 2016 Jan 1:060608.

93. Chaves M, Sontag ED, Albert R. Methods of robustness analysis for Boolean models of gene control networks. Syst Biol. 2006 Jul; 153(4):154–67.

94. Espinosa-Soto C, Padilla-Longoria P, Alvarez-Buylla ER. A gene regulatory network model for cell-fate determination during Arabidopsis thaliana flower development that is robust and recovers experimental gene expression profiles. Plant Cell. 2004 Nov 1; 16(11):2923–39. Epub 2004 Oct 14. https://doi.org/10.1105/tpc.104.021725 PMID: 15486106

95. Balleza E, Alvarez-Buylla ER, Chaos A, Kauffman S, Shmulevich I, Aldana M. Critical dynamics in genetic regulatory networks: examples from four kingdoms. PLoS One. 2008 Jun 18; 3(6):e2456. https://doi.org/10.1371/journal.pone.0002456 PMID: 18560561

96. Benítez M, Espinosa-Soto C, Padilla-Longoria P, Alvarez-Buylla ER. Interlinked nonlinear subnetworks underlie the formation of robust cellular patterns in Arabidopsis epidermis: a dynamic spatial model. BMC Syst Biol. 2008 Nov 17; 2(1):98.

97. Ortiz-Gutiérrez E, García-Cruz K, Azpeitia E, Castillo A, de la Paz Sánchez M, Álvarez-Buylla ER. A dynamic gene regulatory network model that recovers the cyclic behavior of Arabidopsis thaliana cell cycle. PLoS Comput Biol. 2015 Sep 4; 11(9):e1004486. https://doi.org/10.1371/journal.pcbi.1004486 PMID: 26340681

98. Grigg SP, Galinha C, Kornet N, Canales C, Scheres B, Tsiantis M. Repression of apical homeobox genes is required for embryonic root development in Arabidopsis. Curr Biol. 2009 Sep 15; 19(17):1485–90. Epub 2009 Jul 30. https://doi.org/10.1016/j.cub.2009.06.070 PMID: 19646874

99. Bennett T, van den Toorn A, Willemsen V, Scheres B. Precise control of plant stem cell activity through parallel regulatory inputs. Development. 2014 Nov 1; 141(21):4055–64. Epub 2014 Sep 25. https://doi.org/10.1242/dev.110148 PMID: 25256342

100. Wang JW, Wang LJ, Mao YB, Cai WJ, Xue HW, Chen XY. Control of root cap formation by microRNA-targeted auxin response factors in Arabidopsis. Plant Cell. 2005 Aug 1; 17(8):2204–16. Epub 2005 Jul 8. https://doi.org/10.1105/tpc.105.033076 PMID: 16006581

101. Müller CJ, Valdés AE, Guodong WA, Ramachandran P, Beste L, Uddenberg D, et al. PHABULOSA mediates an auxin signaling loop to regulate vascular patterning in Arabidopsis. Plant physiol. 2015 Dec 4:pp-01204. Epub 2015 Dec 4.

102. Forzani C, Aichinger E, Sornay E, Willemsen V, Laux T, Dewitte W, Murray JA. WOX5 suppresses CYCLIN D activity to establish quiescence at the center of the root stem cell niche. Current Biology. 2014 Aug 18; 24(16):1939–44. https://doi.org/10.1016/j.cub.2014.07.019 PMID: 25127220

103. Albert R, Othmer HG. The topology of the regulatory interactions predicts the expression pattern of the segment polarity genes in Drosophila melanogaster. J Theor Biol. 2003 Jul 7; 223(1):1–8. PMID: 12782112

104. Kauffman SA. Metabolic stability and epigenesis in randomly constructed genetic nets. J Theor Biol. 1969 Mar 31; 22(3):437–67. PMID: 5803332

105. Alvarez-Buylla ER, Benítez M, Davila EB, Chaos A, Espinosa-Soto C, Padilla-Longoria P. Gene regulatory network models for plant development. Curr Opin Plant Biol. 2007 Feb 28; 10(1):83–91. Epub 2006 Dec 4. https://doi.org/10.1016/j.pbi.2006.11.008 PMID: 17142086

106. Alvarez-Buylla ER, Azpeitia E, Barrio R, Benítez M, Padilla-Longoria P. From ABC genes to regulatory networks, epigenetic landscapes and flower morphogenesis: making biological sense of theoretical

approaches. InSeminars in cell & developmental biology 2010 Feb 28 (Vol. 21, No. 1, pp. 108–117). Academic Press.

107. Albert R, Wang RS. Discrete dynamic modeling of cellular signaling networks. Methods in enzymology. 2009 Dec 31; 467:281–306. https://doi.org/10.1016/S0076-6879(09)67011-7 PMID: 19897097

108. Alvarez-Buylla ER, Azpeitia E, Barrio R, Benítez M, Padilla-Longoria P. From ABC genes to regulatory networks, epigenetic landscapes and flower morphogenesis: making biological sense of theoretical approaches. InSeminars in cell & developmental biology 2010 Feb 28 (Vol. 21, No. 1, pp. 108–117). Academic Press.

109. Müssel C, Hopfensitz M, Kestler HA. BoolNet—an R package for generation, reconstruction and analysis of Boolean networks. Bioinformatics. 2010 May 15; 26(10):1378–80. Epub 2010 Apr 7. https://doi.org/10.1093/bioinformatics/btq124 PMID: 20378558

110. Sanchez-Corrales YE, Alvarez-Buylla ER, Mendoza L. The Arabidopsis thaliana flower organ specification gene regulatory network determines a robust differentiation process. J Theor Biol. 2010 Jun 7; 264(3):971–83. Epub 2010 Mar 18. https://doi.org/10.1016/j.jtbi.2010.03.006 PMID: 20303988

111. Middleton AM, King JR, Bennett MJ, Owen MR. Mathematical modelling of the Aux/IAA negative feedback loop. Bull Math Biol. 2010 Aug 1; 72(6):1383–407. Epub 2010 Feb 5. https://doi.org/10.1007/s11538-009-9497-4 PMID: 20135237
